# Methylation of rRNA as a host defense against rampant group II intron retrotransposition

**DOI:** 10.1186/s13100-021-00237-z

**Published:** 2021-03-07

**Authors:** Justin M. Waldern, Dorie Smith, Carol Lyn Piazza, E. Jake Bailey, Nicholas J. Schiraldi, Reza Nemati, Dan Fabris, Marlene Belfort, Olga Novikova

**Affiliations:** 1 Department of Biological Sciences and RNA Institute, University at Albany, 1400 Washington Avenue, Albany, NY 12222 USA; 2 Current address: Department of Biology, University of North Carolina, 270 Bell Tower Drive, Chapel Hill, NC 27599 USA; 3 Academic and Research Computing Center, Information Technology Services, University at Albany, 1400 Washington Avenue, Albany, NY 12222 USA; 4 Department of Chemistry, University at Albany, 1400 Washington Avenue, Albany, NY 12222 USA; 5 Current address: Biogen, 125 Broadway, Cambridge, MA 02142 USA; 6 Current address: Department of Chemistry, University of Connecticut, 55 N. Eagleville Road, Storrs, CT 06268 USA; 7 Department of Biomedical Sciences, School of Public Health, University at Albany, 1400 Washington Avenue, Albany, NY 12222 USA; 8 Current address: Biology Department, SUNY Buffalo State College, 1300 Elmwood Avenue, Buffalo, NY 14222 USA

**Keywords:** Mobile genetic elements, Retrotransposons, Ribosomes, RNA splicing

## Abstract

**Background:**

Group II introns are mobile retroelements, capable of invading new sites in DNA. They are self-splicing ribozymes that complex with an intron-encoded protein to form a ribonucleoprotein that targets DNA after splicing. These molecules can invade DNA site-specifically, through a process known as retrohoming, or can invade ectopic sites through retrotransposition. Retrotransposition, in particular, can be strongly influenced by both environmental and cellular factors.

**Results:**

To investigate host factors that influence retrotransposition, we performed random insertional mutagenesis using the IS*S1* transposon to generate a library of over 1000 mutants in *Lactococcus lactis*, the native host of the Ll.LtrB group II intron. By screening this library, we identified 92 mutants with increased retrotransposition frequencies (RTP-ups). We found that mutations in amino acid transport and metabolism tended to have increased retrotransposition frequencies. We further explored a subset of these RTP-up mutants, the most striking of which is a mutant in the ribosomal RNA methyltransferase *rlmH,* which exhibited a reproducible 20-fold increase in retrotransposition frequency. In vitro and in vivo experiments revealed that ribosomes in the *rlmH* mutant were defective in the m3Ψ modification and exhibited reduced binding to the intron RNA.

**Conclusions:**

Taken together, our results reinforce the importance of the native host organism in regulating group II intron retrotransposition. In particular, the evidence from the *rlmH* mutant suggests a role for ribosome modification in limiting rampant retrotransposition.

**Supplementary Information:**

The online version contains supplementary material available at 10.1186/s13100-021-00237-z.

## Background

Group II introns are self-splicing mobile genetic elements found in archaeal, bacterial and organellar genomes, and are believed to be the ancestors of eukaryotic spliceosomal introns and retrotransposons [1–4]. The catalytically active intron RNA, together with its intron-encoded protein (IEP), form a ribonucleoprotein (RNP) complex where the IEP assists with splicing and mobility of the intron [2, 5]. Splicing occurs via two reversible transesterification reactions, and results in the formation of the group II intron lariat and ligated exons [2]. After splicing, the intron can reverse splice into target DNA with the help of the IEP, either through a process called retrohoming, where the intron specifically invades a target DNA homing site, or through a less efficient process called retrotransposition (RTP), where it invades ectopic sites, frequently at replication forks in the chromosome [1–3, 6–8].

Group II introns within RNPs are highly structured RNA molecules. The RNA structure is essential for activity and consists of a highly conserved fold comprising six domains (DI-DVI) [2, 9]. Domain I (DI) contains the exon binding sites (EBS), which interact with the intron binding sites (IBS) of the exons to target splicing and reverse-splicing [2]. Domain IV (DIV) contains the open reading frame (ORF) that encodes the IEP, as well as the ribosome binding site (RBS) required for IEP translation, and the IEP binding site, needed for splicing and reverse splicing. After translation, the IEP binds in DIV and occludes the RBS, which represses further translation of the IEP [10]. The spliced and fully-formed RNP can then act as a retroelement capable of invading DNA.

Both retrohoming and retrotransposition have been carefully dissected using the Ll.LtrB group II intron from *Lactococcus lactis* in the model organism, *Escherichia coli,* which has revealed the importance of various host factors in both inhibiting and facilitating retromobility [6, 11–16]. For example, one study revealed that RNase E downregulates group II intron retromobility by degrading the intron RNA [15], while further work demonstrated how ribosomes can bind to the intron and block this effect [16]. Yet, these effects can change based upon the host background, even to the extent that retrotransposition can proceed through different mechanistic pathways in different host organisms [13]. In its native host, *L. lactis*, the Ll.LtrB intron undergoes retrotransposition mainly into single-stranded DNA utilizing replication forks with Okazaki fragments serving as primers for reverse transcription, predominantly through an endonuclease-independent pathway [6, 7]. However, in *E. coli* the intron undergoes retrotransposition mainly through an endonuclease-dependent pathway, drawing many similarities to site-specific retrohoming [13]. Taken together, these studies have shown that retrotransposition is sensitive to the host environment and that host genes play an important role in regulating retromobility.

**Fig. 1:**
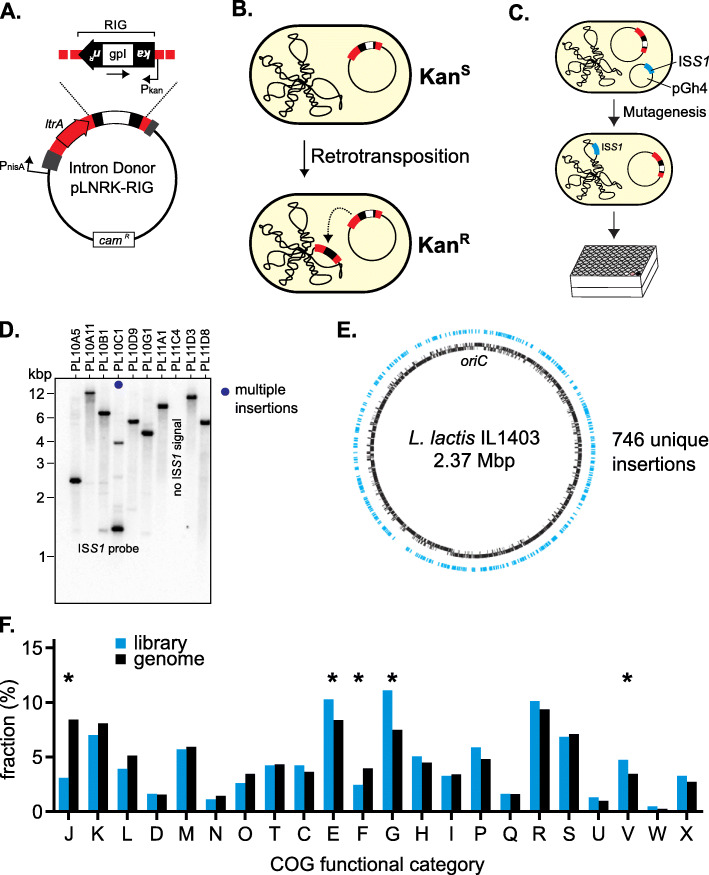
Mutant library generation and characterization. **a.** Intron-donor plasmid pLNRK-RIG. The retrotransposition indicator gene (RIG) is located within the Ll.LtrB group II intron (red), which is under the control of the nisin promotor (P_nisA_). RIG contains a kanamycin resistance gene (black boxed arrow) with its own promoter (P_kan_ opposite to the direction of transcription), interrupted by a group I intron (gpI, white box). The Ll.LtrB intron is flanked by partial native exons and contains its own intron-encoded protein, LtrA. The pLNRK backbone contains a chloramphenicol resistance gene (*cam*^*R*^) [21]. **b.** Retrotransposition assays identify retrotransposition events through the acquisition of kanamycin resistance (Kan^R^) after the group I intron is spliced out. **c.** IS*S1*-mediated mutagenesis schematic. Cells containing the intron donor plasmid, pLNRK-RIG, were transformed with pGh4:IS*S1* and selected for erythromycin resistance. Cultures containing both plasmids were grown to allow for transposition of the IS*S1* transposon, followed by a temperature shift to remove the temperature sensitive pGh4:IS*S1*, screened for the loss of erythromycin resistance, and storage in 96-well plates alongside various controls. For details, see Additional File 1: Fig. S1 **d.** Southern blot analysis of the library for IS*S1* copy number. A random subset of 50 IS*S1* mutants were probed for IS*S1* insertions using Southern blotting. Dark blue dot indicates a strain had multiple insertions. For all data, see Additional File 1: Fig. S2. **e.** Distribution of the IS*S1* mutant library insertions (blue) around the *L. lactis* IL1403 chromosome (black). Sequencing and analysis of the library resulted in the identification of 746 unique IS*S1* insertions. **f.** Comparison of functional coverage of the IS*S1* mutant library (blue) against the *L. lactis* genome (black), based on relative abundance of COG categories. Asterisks represent a significant difference (*p* < 0.05) as determined by a hypergeometric test. A key of all COG categories is in Additional File 1: Table S3

Due to the differences in retrotransposition across hosts, efforts have been undertaken to clarify the relationship between the Ll.LtrB intron and its native host, *Lactococcus lactis*. The Ll.LtrB intron of *L. lactis* is found on the conjugative plasmid pRS01 and is capable of dissemination through conjugation [17–20]. Since retrotransposition is a low-frequency event, it is often studied with a genetic reporter intron donor plasmid, pLNRK-RIG (Fig. 1a and Table 1). This plasmid contains a retrotransposition indicator gene (RIG), which allows the quantification of retrohoming and retrotransposition frequency based on acquisition of kanamycin resistance [6, 21] (Fig. 1b). Using this reporter, it has been shown that the relaxase in which the Ll.LtrB intron natively resides not only nicks its own pRS01 plasmid DNA to initiate conjugation, but also nicks off-target DNA sites, promoting retrotransposition [21].

**Table 1:** Bacterial strains and plasmids

Bacteria	Relevant characteristics; comments	Reference
*L. lactis* IL1403	plasmid free; recipient strain, strain for all RTP assays; sequenced genome is available (GenBank: NC_002662)	[22]
*E. coli* DH5α	F^−^ *endAI recAl hsdRl7* (rK^−^ mK^−^) *deoR supE44 thi*^*−*^*J gyrA96 relA*	Gibco-BRL
**Plasmids**	**Relevant characteristics; comments**	**Reference**
pLNRK-RIG	*L. lactis*/*E. coli* shuttle vector contains nisin-inducible promoter, *nisR nisK*, Cam^R^, RIG cassette, intron donor	[21]
pGh4:IS*S1*	pG + host replicon, thermosensitive (replicates at 28 °C but lost above 37 °C), Erm^R^	[23]
pRS01	co-integrant of conjugative plasmid pRS01 and pTRK28, Erm^R^	[24]
pLNRK-GFP	Derived from pLNRK-RIG with green fluorescent protein (GFP) replacing the entire intron and RIG cassette. Cam^R^	This study
pMN1343	pMN1343 Spc^R^, contains intron homing site	[25]
pGh5: P_nisA_	pG + host replicon, Erm^R^, backbone of pGh5 from [23], with IS*S1* removed, P_nisA_ inserted. Used as empty vector control in the wild-type background in complementation assays.	[23] and this study
pGh5: P_nisA_ *rlmH*	pGh5:P_nisA_ with the *rlmH* gene inserted under control of the P_nisA_ promoter. Used for complementation assays.	[23] and this study
pLNRK-SA	pLNRK smEx::Ll.LtrB ΔORF (SA)-nLIC; expression construct for Ll.LtrB intron with streptavidin aptamer in domain IVb, Cam^R^	[26]

To further investigate the relationship between the Ll.LtrB intron and host functions, we created and screened a mutant library in the intron’s native host *L. lactis*. Mutants that showed up-regulation of retrotransposition frequencies relative to the wild type (RTP-ups) were further characterized in an interaction network. Then, 12 mutants, from varying functional categories, were chosen for further analysis. From these mutants, the *rlmH*::IS*S1* mutant, defective in ribosomal RNA (rRNA) methylation, consistently demonstrated very high retrotransposition frequencies, despite having no effect on site-specific retrohoming. Ribosomes from the *rlmH*::IS*S1* mutant were defective in their ability to bind intron RNA both in vitro and in vivo, suggesting that normal ribosome-intron interactions reduce retrotransposition frequency, leading us to the hypothesis that ribosomes function to protect the genome against rampant group II intron retrotransposition in the native host.

## Results

### Mutant library construction and initial characterization

To identify host genes affecting Ll.LtrB group II intron retrotransposition efficiency, transposon insertion mutants were generated in the *L. lactis* IL1403 strain carrying the intron donor plasmid (pLNRK-RIG, Fig. 1a) and a pG^+^host plasmid carrying the insertion element IS*S1* (pGh4:IS*S1*) [23] (Fig. 1c and Table 1; Additional File 1: Fig. S1). In total, 1006 individual *L. lactis* IL1403 IS*S1* mutant strains were produced and arrayed into 11 96-well plates, PL1-PL11 (PL – plate). Southern blot analysis of 50 IS*S1* strains randomly chosen from the library confirmed the presence of IS*S1* insertions and their random distribution in the bacterial chromosome, with 20% of these strains (9 out of 45) harboring more than one IS*S1* insertion (Fig. 1d; Additional File 1: Fig. S2A and Table S1), which is consistent with the originally reported occurrence for multicopy transposition [23]. We also established the stability and vertical transmission of the IS*S1* insertions by tracing them through 12 bacterial generations (Additional File 1: Fig. S2B). Overall, these results demonstrate that we produced a library of stable mutants, consisting largely of single insertions.

To further characterize the library of *L. lactis* IL1403 IS*S1* mutant strains we used targeted high-throughput sequencing to identify IS*S1* insertion sites (Additional File 1: Fig. S3). To minimize the number of sequencing reactions, we utilized the heuristic Straight Three strategy for orthogonal pooling and mapping [27] (Additional File 1: Fig. S3). After sequencing, initial data processing, and filtering, we identified 757 IS*S1* insertion sites, 746 of which were uniquely mapped in the *L. lactis* IL1403 genome (Fig. 1e; Additional File 2). Comparison of the IS*S1* flanking regions showed no significant consensus among sequences, although nucleotides 15 and 16 bases both upstream and downstream of the IS*S1* insertion site were found to be adenine and thymine at somewhat higher frequencies than expected (Additional File 1: Fig. S4). Nevertheless, the largely random insertion by IS*S1* in *L. lactis* IL1403 confirms previous reports [23, 28].

Next, we wanted to determine if there were any functional biases in the mutant library. To compare gene functionality on a broad scale, we performed a Clusters of Orthologous Groups (COG) analysis, which clusters genes into groups based on protein function [29]. We first compared the COG distribution of the IS*S1* mutant library against that of *L. lactis* IL1403 (Fig. 1f; Additional File 1: Table S2) and made statistical comparisons using a hypergeometric test. Several categories were significantly enriched in the library (*p* < 0.05), including E, G, and V. COG categories E and G represent functionalities related to transport and metabolism of amino acids and carbohydrates respectively (Additional File 1: Tables S2-S3). COG category V consists of proteins involved in host defense mechanisms, such as restriction-modification systems or drug efflux pumps (Additional File 1: Table S3). We observed two categories significantly underrepresented in the library (p < 0.05): F and J (Fig. 1f; Additional File 1: Table S3). Category F consists of proteins with functions related to nucleotide transport and metabolism, whereas the functionalities in category J relate to translation, ribosome structure and ribosome biogenesis (Additional File 1: Table S3). Despite these statistically significant differences, most functional categories have very similar representation between our mutant library and the genomic background of *L. lactis* IL1403.

### Identifying mutants with elevated intron retrotransposition frequencies

**Fig. 2:**
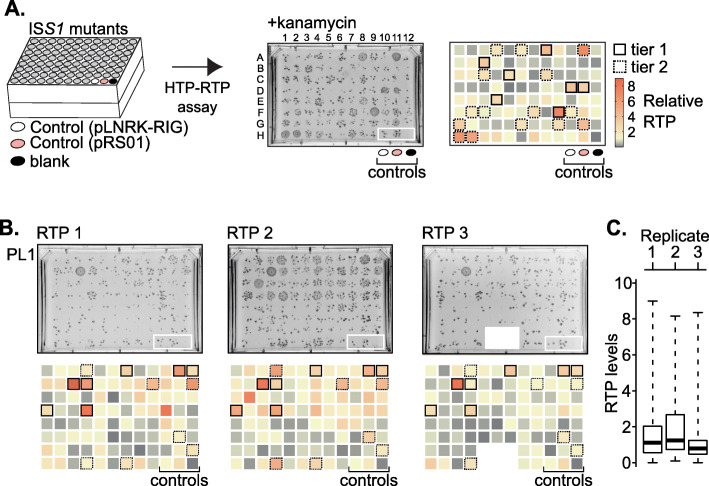
RTP-up mutant identification. **a.** High-throughput RTP (HTP-RTP) assay. All IS*S1* mutant and control strains were grown, induced and plated on kanamycin to identify mutants with a putative RTP-up phenotype. Spot size on selective media was quantified relative to the wild-type pLNRK-RIG control (white oval) and visualized as double gradient heatmaps (min, 0; max, 9; baseline, 1). Additional controls included the same strain with pRS01 with an “up” phenotype [21] (pink oval) and a blank well containing only media (black oval). Mutants were ranked into tiers based on the boxplot analysis of retrotransposition frequencies within each plate (Additional File 1: Fig. S5 and Table S4). A box with a solid black outline identifies mutants in the first tier, whereas a dotted outline represents the second tier. **b.** Results of three independent HTP-RTP assays (RTP 1 – RTP 3) for plate 1 (PL1). Heatmaps for each plate are shown. A contaminated area of the RTP3 plate was excluded from analysis (white area in PL1 RTP 3). Results of the HTP-RTP assays for all plates are in Additional File 1: Fig. S5 and Table S4. **c.** Boxplot analysis of PL1. RTP levels are reported relative to the wild type. Box and whisker plots outline the quartiles, with the bold line within each box as the median. All boxplots are in Additional File 1: Fig. S5

To identify potential host mutants with altered intron retromobility activity, retrotransposition (RTP) assays were performed in a high-throughput (HTP) 96-well plate format followed by spot plating on rectangular GM17 agar plates containing 160 μg/mL kanamycin (Fig. 2a; Additional File 1: Fig. S5). HTP-RTP assays were performed for plates PL1 through PL11 simultaneously and repeated independently three times using a liquid handler to ensure uniformity of spotting. Although some mutants producing a relatively high number of colonies on HTP-RTP assay plates could be identified by eye, we utilized SGAtools for image analysis and spot size quantification [30] (Fig. 2b; Additional File 1: Fig. S5 and Table S4). For each plate, spot size scores were calculated with SGAtools and normalized to the spot size score produced by the wild-type pLNRK-RIG strain from the same plate. Thus, normalized colony size scores from plates selecting for retrotransposition events represent relative intron retrotransposition levels.

Next, we used a boxplot analysis (Fig. 2c), which allowed us to investigate the relative intron retrotransposition value distribution among datasets, and to select mutants showing consistently higher relative levels. We limited ourselves to mutants with increased retrotransposition frequencies (RTP-up mutants) because those that yielded reduced frequencies produced inconsistent phenotypes. The mutants were divided into tiers based on the intensity and consistency of their retrotransposition phenotype. The first tier of mutants was defined as those with relative retrotransposition levels between the third quartile (Q3) and fourth quartile (Q4, maximum) in all three HTP-RTP assays based on the boxplot analysis; mutants were in the second tier if their retrotransposition levels were between Q3 and Q4 in two HTP-RTP assays but between the median (Q2) and Q3 once (Fig. 2; Additional File 1: Fig. S5 and Table S4). Mutants from the first and second tier were designated as RTP-up mutants and selected for further analysis.

In total, 155 strains were identified as *L. lactis* IL1403 IS*S1* mutant strains exhibiting consistently elevated levels of retrotransposition. These RTP-up mutants were checked for the presence of multicopy IS*S1* using Southern blotting as described above, and mutants with more than a single IS*S1* per chromosome were purged from the list (Additional File 1: Fig. S6 and Table S4). The IS*S1* insertion was verified by a two-stage process of inverse PCR coupled with individual sequencing, followed by a verification PCR with primers flanking the insertion site (Additional File 1: Fig. S7-S8 and Table S5). Any mutants that could not be verified through this process were omitted from subsequent investigation, leaving 92 mutants for further analysis (Additional File 3).

**Fig. 3:**
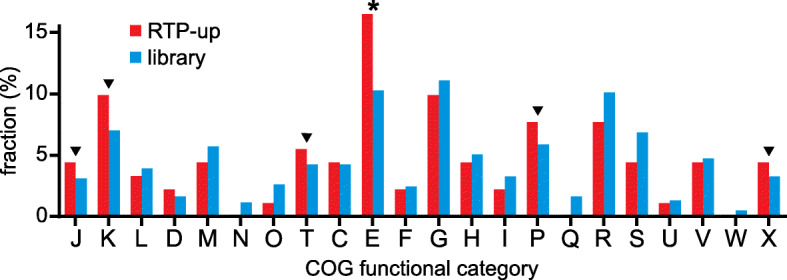
Functional category comparison of RTP-up mutants. Comparison of the COG distribution of RTP-up mutants against the mutant library. An asterisk represents a statistically significant difference (*p* < 0.05) using a hypergeometric test, whereas triangles point to categories that are enriched in RTP-up mutants, but not statistically different. A significant difference is observed in COG category E, which corresponds to amino acid transport and metabolism. Enrichment of RTP-ups is also observed in several categories: J, translation, ribosomal structure and biogenesis; K, transcription; T, signal transduction; P, inorganic ion transport and metabolism; and X, mobile genetic elements. A key of all COG categories is in Additional File 1: Table S3

To identify functional enrichment within the validated RTP-up mutants, we compared the distribution of COG categories of the RTP-up mutants against that of the whole IS*S1* mutant library. We observed a dramatic and statistically significant enrichment of RTP-up mutants in genes relating to amino acid transport and metabolism (COG category E) (Fig. 3; Additional File 1: Tables S2-S3). Several other COG categories had an elevated relative abundance of RTP-up mutants, despite not being statistically significant, including categories related to translation (J), transcription (K), signal transduction (T), inorganic ion transport and metabolism (P), and mobile genetic elements (X) (Fig. 3; Additional File 1: Tables S2-S3).

To probe a possible master regulator of retrotransposition, we generated a protein interaction network of the RTP-up mutants and the mutant library background using Cytoscape and the STRING database [31, 32]. We submitted all the protein sequences from mutants in the IS*S1* mutant library and generated a network consisting of 503 nodes, representing unique protein sequences. Within this network, 52 nodes showed no interactions. We grouped the network by COG category and colored RTP-up mutants red, enabling visualization of the proportion of RTP-up mutants within each COG category (Additional File 1: Fig. S9). Despite numerous interactions identified within the STRING database, no master regulator connecting many RTP-up mutants emerged from the network that could explain the RTP-up phenotypes.

### Mutants selected for further analysis

We next focused on 12 individual mutants from the list of 92 that showed elevated retrotransposition in the HTP-RTP assays and featured particularly interesting gene functionality (Table 2). For example, we selected *spoT*::IS*S1* because previous work has shown *relA* and *spoT* to have an effect on retrotransposition of the Ll.LtrB intron in *E. coli* [33]. We selected *hisH*::IS*S1* and *rpoE*::IS*S1* as their gene functionality is relevant to central metabolism [34, 35], and they represent COG categories E and K respectively, which were both overrepresented in the RTP-up mutants relative to the library background (Fig. 3). The *coiA* gene, which codes for a competence protein [36], was selected because it appeared three times within the IS*S1* mutant library with three distinct insertion sites at different locations within the *coiA* gene, with varying RTP frequencies. We also selected an insertion into the origin of replication, *oriC*, since retrotransposition has previously been shown to be linked to DNA replication [6, 7]. Similarly, we selected *recT*::IS*S1* because of its function in DNA repair [37]. One cluster of mutants of particular interest were those related to translation (COG category J). Despite COG category J being underrepresented in the library (Fig. 1F), four mutants in this category fit the criteria for consistently high results in the HTP-RTP assay: *ybaK*::IS*S1*, *rsmB*::IS*S1*, *rsmE*::IS*S1*, and *rlmH*::IS*S1*. The *ybaK* gene codes for an aminoacyl-tRNA deacylase [38], whereas the other three code for ribosomal RNA methylases [39] (Table 2).

**Table 2:** Top-12 *L. lactis* IL1403 mutants with increased RTP frequencies

	IS***S1*** Insertion Position	Gene	Library tag	Gene product	Protein Accession	Relative RTP Frequency	COG Category
**1**	1,959,088	*rsmB*	PL1B3	16S rRNA cytosine methyltransferase (m5C) at position 967	WP_010906185.1	1.3+/−0.7	J, K
**2**	108,704	*spoT*	PL5A3	Bifunctional (p)ppGpp synthetase/guanosine-3′5’-bis(diphosphate) 3′ - pyrophosphohydrolase	WP_010905119.1	4.4+/−1.6	K, T
**3**	1,785,586	*coiA*	PL5A12	Competence protein, contains a predicted nuclease domain	WP_010906110.1	5.7+/−3.2	R
**4**	623,717	*rpoE*	PL6A8	DNA-directed RNA polymerase subunit delta	WP_003129551.1	9.3+/−6.6	K
**5**	105,498	*rsmE*	PL6B3	16S rRNA uracil methyltransferase (m3U) at position 1498	WP_010905117.1	1.7+/−1.3	J
**6**	1,785,385	*coiA*	PL6F9	Competence protein, contains a predicted nuclease domain	WP_010906110.1	6.8+/−3.6	R
**7**	2,202,641	*rlmH*	PL7B7	23S rRNA pseudouridine methyltransferase (m3Ψ) at position 1915	WP_003130585.1	20.1+/−1.5	J
**8**	1,235,064	*hisH*	PL7D6	Imidazole glycerol phosphate synthase subunit	WP_010905832.1	81.3+/−75.3	E
**9**	453,621	*recT*	PL7E1	Recombinational DNA repair protein	WP_010905348.1	10.5+/−4.3	L
**10**	62	*oriC*	PL8C4	Origin of replication, intergenic		5.3+/−5.2	
**11**	315,800	*ybaK*	PL9C11	Aminoacyl-tRNA deacylase	WP_003131680.1	3.8+/−3.4	J
**12**	1,785,006	*coiA*	PL11D6	Competence protein, contains a predicted nuclease domain	WP_010906110.1	31.1+/−34.4	R

**Fig. 4:**
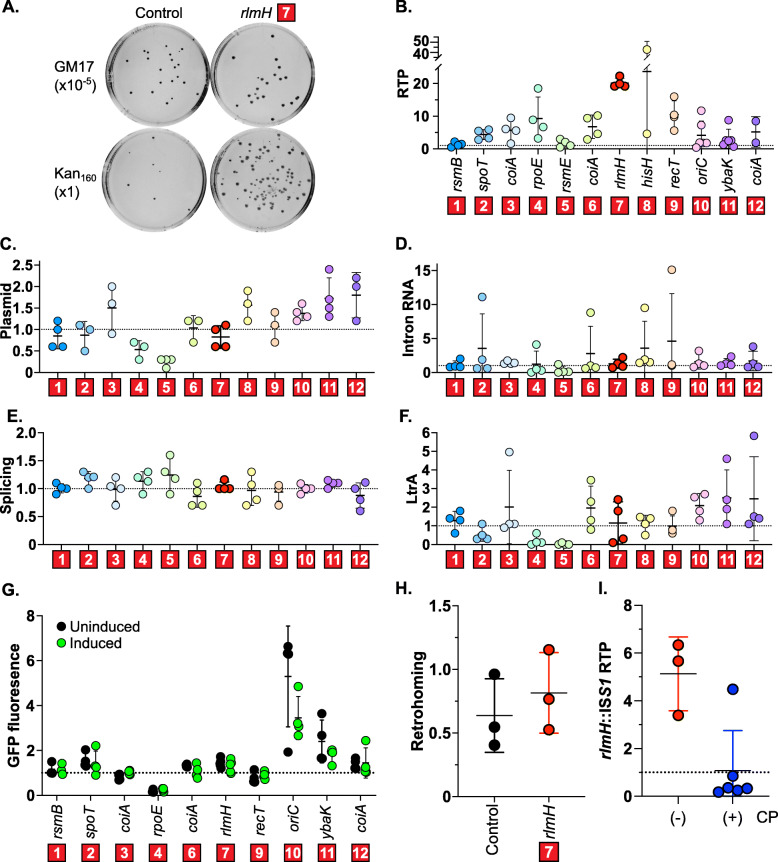
Characterization of top-12 mutants relative to control. **a.** Retrotransposition analysis. RTP assays were performed in low-throughput for each mutant to quantify retrotransposition frequencies. Representative plates are shown for the *rlmH*::IS*S1* mutant and the isogenic wild-type control strain. Retrotransposition frequencies were calculated by dividing the CFU on selective media by that on nonselective media. **b.** Retrotransposition frequencies. Each mutant was evaluated relative to wild-type IL1403 containing pLNRK-RIG, omitting extreme outliers. Individual data points are shown, with a horizontal bar at the mean with standard deviation. For all data points, see Additional File 1: Fig. S10. **c.** DNA dot blots. Dot blots were performed to measure the amount of donor plasmid (pLNRK-RIG). For data, see Additional File 1: Fig. S11. **d.** Northern blots. Intron RNA level (precursor and spliced intron) was normalized against 16S rRNA. For gels, see Additional File 1: Fig. S12. **e.** Primer extension analysis. Splicing efficiency was calculated as the amount of spliced intron divided by the sum of spliced intron plus precursor. For gels, see Additional File 1: Fig. S13. **f.** Western blots. The amount of intron-encoded protein, LtrA, was normalized to total protein. For blots, see Additional File 1: Fig. S14. **g.** Induction from the P_nisA_ promoter. The plasmid pLNRK-GFP reported GFP fluorescence. Values are normalized GFP of each mutant relative to the wild type after 3 h of induction (green, induced; black, uninduced). For all data, see Additional File 1: Fig. S15. **h.** Retrohoming analysis. Assays were performed with wild type and *rlmH*::IS*S1* containing pLNRK-RIG (intron donor) and pMN1343 (homing site). No significant difference was observed between *rlmH*::IS*S1* and control (*p* = 0.51). **i.** Complementation assays. Assays were performed with (blue) or without (red) complementation plasmids (CP) and reported relative to the respective wild-type control. Complementation significantly reduced the RTP frequency of *rlmH*::IS*S1* (*p* < 0.05)

First, we performed a more quantitative RTP assay in larger cultures and plated on GM17 with and without kanamycin to calculate retrotransposition frequencies for each mutant, dividing the number of colony-forming units (CFU) of retrotransposition events on plates containing kanamycin, by total CFU on nonselective GM17 (Fig. 4a-b and Table 2; Additional File 1: Fig. S10). Some retrotransposition phenotypes varied between the HTP-RTP assay and the individual characterization, which could be due to differing growth conditions or to individual RTP assays accounting for cell growth, whereas the initial HTP-RTP assay measured strictly survival on kanamycin. For example, of the three rRNA modification mutants, two, *rsmB*::IS*S1* and *rsmE*::IS*S1,* had consistently elevated retrotransposition in the HTP-RTP assay but had background levels of retrotransposition in the individual characterization (Fig. 4b; Additional File 1: Fig. S10). In contrast, *rlmH*::IS*S1* was repeatedly greatly elevated in retrotransposition in both assays. All other mutants had elevated, albeit variable retrotransposition frequencies, with *rlmH*::IS*S1* as the clear “winner” among the 12 selected mutants, displaying consistently 20-fold elevated retrotransposition frequencies (Fig. 4b and Table 2; Additional File 1: Fig. S10).

Next, we sought to eliminate the possibility that over-production of the intron could explain the increase in retrotransposition frequency. Therefore, we examined the amount of intron donor plasmid, intron RNA levels, splicing, the amount of intron-encoded protein (LtrA), and reporter inducibility in each mutant (Fig. 4c-g; Additional File 1: Fig. S11-S14). Thus, we used dot blots to measure plasmid copy number (Fig. 4c; Additional File 1: Fig. S11), Northern blots to assess intron RNA levels (Fig. 4d; Additional File 1: Fig. S12), primer-extension analysis to assay splicing efficiency (Fig. 4e; Additional File 1: Fig. S13), and Western blots to measure LtrA levels (Fig. 4f; Additional File 1: Fig. S14). Finally, to analyze how well the mutants express the reporter construct, we replaced the group II intron with the green fluorescent protein (GFP) gene and measured GFP fluorescence under nisin induction (Fig. 4g and Table 1; Additional File 1: Fig. S15). Although RNA splicing was uniform across the mutants, several mutants had elevated levels of donor plasmid, intron RNA, or LtrA (*hisH*::IS*S1*, *oriC*::IS*S1*, *ybaK*::IS*S1*, and *coiA*::IS*S1*), and two mutants had low levels across the board of assays (*rpoE*::IS*S1* and *rsmE*::IS*S1*). When measuring induction with GFP fluorescence, most mutants behaved similarly to the control, however the *oriC*::IS*S1* and *ybaK*::IS*S1* mutants had elevated expression of the reporter construct, regardless of induction, compared to the control (Fig. 4g; Additional File 1: Fig. S15). In all aspects, the *rlmH*::IS*S1* mutant, with a 20-fold enhancement of retrotransposition levels, had plasmid copy number, intron RNA levels, LtrA, and induction levels comparable to its wild-type control counterpart (Fig. 4a-g).

### Methylation of rRNA affects RTP frequency

We observed a cluster of rRNA methylation mutants with increased retrotransposition in the HTP-RTP screening assay: *rlmH*::IS*S1, rsmB*::IS*S1*, and *rsmE*::IS*S1.* However, in the more quantitative individual RTP assays, *rlmH*::IS*S1* exhibited dramatically increased levels of retrotransposition relative to the control (20.1 +/− 1.5), whereas *rsmB*::IS*S1* and *rsmE*::IS*S1* were within error of the control (1.3 +/− 0.7 and 1.7 +/− 1.3 respectively) (Fig. 4b and Table 2). Therefore, we decided to hone in on how the *rlmH*::IS*S1* mutant affects retrotransposition. First, we were interested if the effect of *rlmH*::IS*S1* was specific to retrotransposition or if it affected retrohoming as well, because that might provide some insight into mechanism of action of RlmH on the retromobility process. We transformed *rlmH*::IS*S1* pLNRK-RIG and the control wild-type pLNRK-RIG strains with a second plasmid containing the homing site, pMN1343 (Table 1) and measured retrohoming in a similar manner to the individual RTP assays. The presence of the homing site makes retrohoming the preferred process by orders of magnitude and the contribution of retrotransposition is negligible by comparison. The retrohoming frequencies of *rlmH*::IS*S1* and the control were not significantly different (Fig. 4h), suggesting that the effect of the *rlmH*::IS*S1* mutant is specific to retrotransposition.

Furthermore, to verify that the effect on retrotransposition is specifically due to *rlmH* and not a pleiotropic effect of the IS*S1* insertion, we performed RTP assays with complementation by adding back the *rlmH* gene (Fig. 4i). To alleviate the stress of maintaining multiple plasmids, we used reduced antibiotic concentrations, which may explain the less dramatic, albeit still substantially increased retrotransposition frequency of *rlmH*::IS*S1* without the complementation plasmid (5.1 +/− 1.5). Upon introduction of the *rlmH* complementation plasmid (Table 1), relative retrotransposition frequencies dropped to control levels (1.1 +/− 1.7) (Fig. 4i). The reduction of retrotransposition frequency upon reintroduction of the *rlmH* gene demonstrates that the mutation in *rlmH* is directly responsible for the retrotransposition phenotype.

**Fig. 5:**
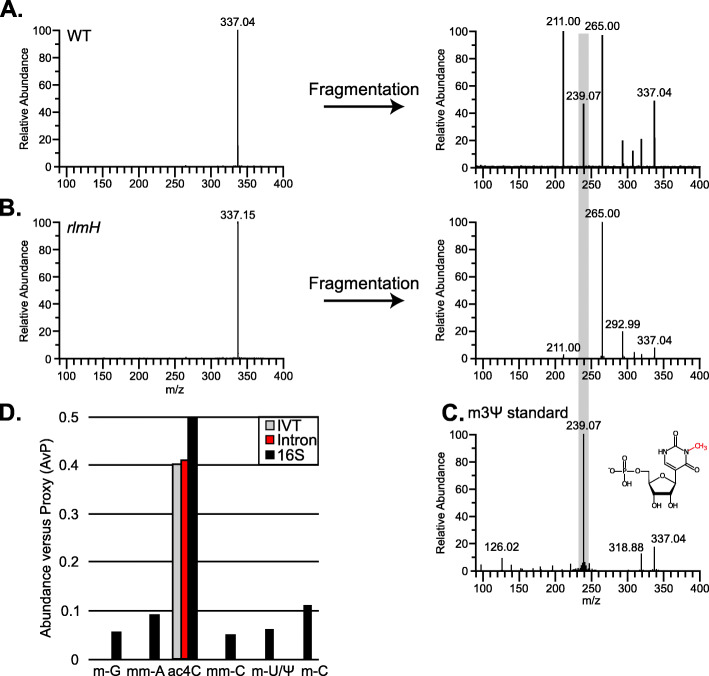
Mass spectrometry validation of *rlmH*::IS*S1* ribosomes and modification profile of the intron RNA. **a.** Isolation spectrum of the signal at 337 m/z from wild-type (WT) rRNA. The isolated precursor ion was submitted to collisional activation, and the observed fragmentation pattern was compared with those recorded for the standards (Additional File 1: Fig. S16). Gray highlighting indicates the presence of the unique 239 m/z signal corresponding to m3Ψ (panel C and Additional File 1: Fig. S16). Other prominent signals, such as 211 and 265 m/z correspond to characteristic fragments generated by methylated uridine/pseudouridine isobars (Additional File 1: Fig. S16). **b.** Isolation and fragmentation spectra of the signal at 337 m/z from RNA isolated from *rlmH*::IS*S1* ribosomes. Gray highlighting indicates where the 239 m/z signal would fall, were the m3Ψ species present in the mutant rRNA. **c.** Fragmentation spectrum obtained from the m3Ψ standard, showing the prominent diagnostic signal at 239 m/z (gray), which is absent in the spectra of the other isobars (Additional File 1: Fig. S16). **d.** Relative abundance (AvP) of ribonucleotide variants detected in intron and/or 16S rRNA. Modifications detected in purified intron RNA (red), 16S rRNA (black), and in vitro-transcribed intron RNA (IVT, gray) are shown. Abbreviations: m-G = methylated guanosine, mm-A = dimethyl adenosine, ac4C = acetyl cytidine, mm-C = dimethyl cytidine, m-U/Ψ = methylated uridine or methylated pseudouridine, m-C = methylated cytidine

Next, we experimentally validated the functionality of the *rlmH* gene in *L. lactis* IL1403 and its knockout in *rlmH*::IS*S1*. In *E. coli*, RlmH converts the pseudouridine at position 1915 of the large rRNA subunit to 3-methylpseudorudine (m3Ψ) on fully assembled 70S ribosomes [40–42], the presence of which could be readily detected by mass spectrometric (MS) analysis. Our strategy entailed extracting 70S rRNA from either wild-type or *rlmH*::IS*S1* mutant cells, performing exonuclease digestion to reduce the RNA to its mononucleotide components, and then analyzing the products by direct infusion nanoflow electrospray (nanospray) MS [43]. To enable the unambiguous discrimination of m3Ψ from a series of natural variants that share the same elemental composition, and thus mass (i.e., Um, m5U, m1Ψ, and m3U), we first analyzed corresponding synthetic standards by tandem mass spectrometry (MS/MS). The data revealed that m3Ψ produced a unique diagnostic fragment at 239 m/z, which was not obtained from any of the other isomers (Additional File 1: Fig. S16). When the 70S samples were examined, both digestion mixtures exhibited abundant signals at 337 m/z, which could in principle correspond to any possible combination of the above isomers (Fig. 5a and b, left panels). When submitted to MS/MS, the 337 m/z precursor ion from the wild-type sample produced a series of fragments corresponding to any possible combination of isomers, including the 239 m/z signal characteristic of m3Ψ (Fig. 5a, right panel and Fig. 5c). In contrast, such a fragment was conspicuously absent from the MS/MS spectrum of the 337 m/z precursor afforded by the *rlmH*::IS*S1* sample (Fig. 5b, right panel). The absence of m3Ψ from the 70S ribosomes of *rlmH*::IS*S1* confirmed that this mutant was a complete knockout of RlmH function, as its 70S ribosomes did not display m3Ψ, whereas wild-type 70S ribosomes did.

In seeking to explain the increased retrotransposition frequency in *rlmH*::IS*S1*, we hypothesized that functional RlmH could be promiscuously modifying the intron RNA in wild-type cells, and thereby limiting retrotransposition. To test this, we purified intron RNA from intron RNPs from wild-type cells for MS analysis (Additional File 1: Fig. S17). Additionally, we purified 16S rRNA, which copurifies with the intron as a positive control, and in vitro-transcribed intron RNA as a negative control. Following RNA isolation, the intron RNA was separated from 16S rRNA using native agarose gel electrophoresis and individual bands representing intron RNA or 16S rRNA were extracted for analysis. All samples, including the negative control, showed the presence of acetylated cytidine (ac4C), which is likely an artifact of the purification method (Fig. 5d). Regardless, in contrast to 16S rRNA, which was clearly methylated, we observed no methylations on the intron RNA (Fig. 5d). Thus, the intron is unmodified in the wild-type background, and the absence of methylations on the intron itself cannot be responsible for changes in retrotransposition frequency of *rlmH*::IS*S1*.

### Ribosome binding to intron RNA is diminished in the rlmH mutant

**Fig. 6:**
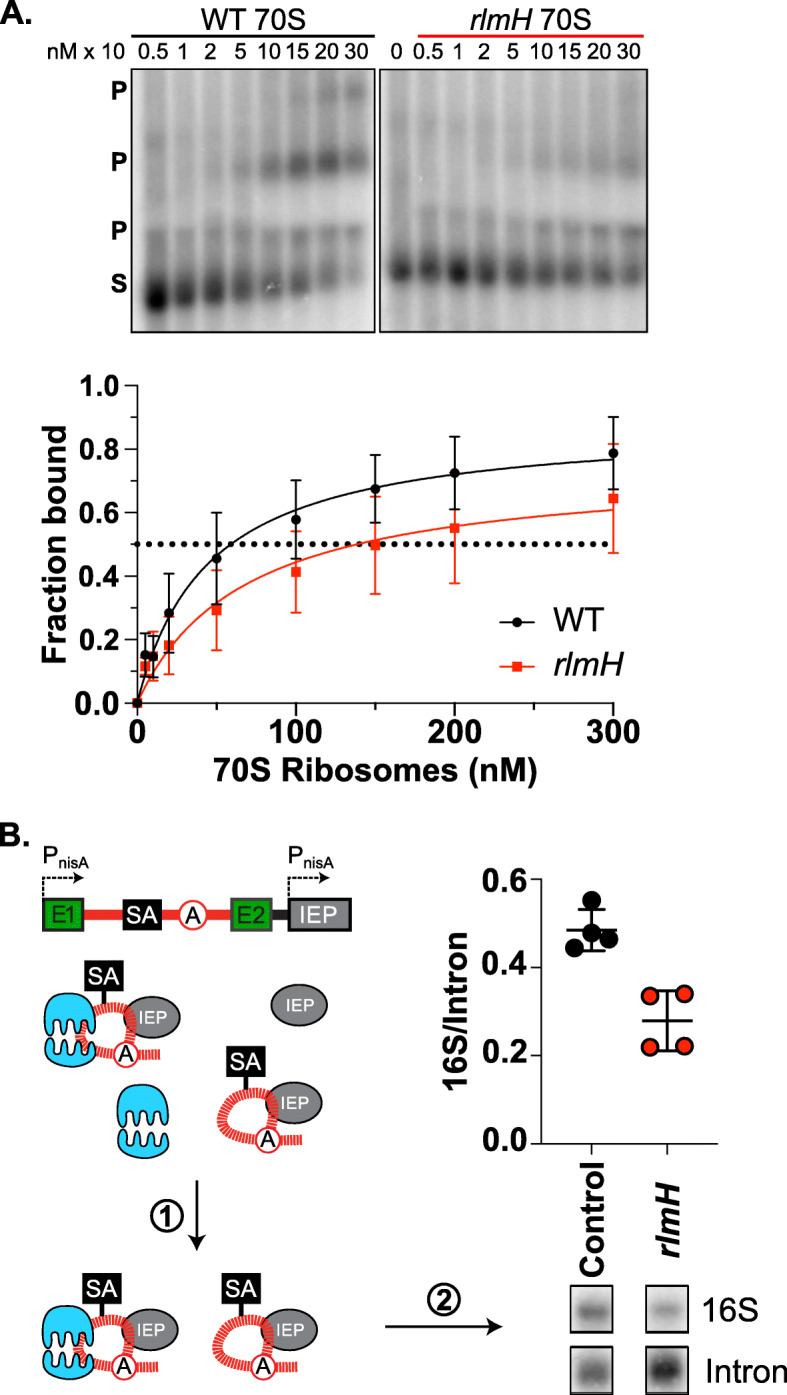
The *rlmH*::IS*S1* ribosomes bind poorly to intron RNA. **a.** In vitro gel-shift assays show that *rlmH*::IS*S1* mutant 70S ribosomes are defective in binding to intron RNA. Gels show wild-type (WT) and *rlmH*::IS*S1* ribosome-intron binding, where substrate (S) is radiolabeled in vitro-transcribed intron substrate, with bound product (P) represented higher in the gel. Increasing concentrations of ribosomes are shown with values across the top. Multiple bound products likely represent various ribosome-bound intron RNA conformations [16]. Below is a binding curve based on quantifying the intensity of the bands in these gels, with the average fraction bound plotted against the concentration of ribosomes. **b.** In vivo intron pull-down is consistent with *rlmH*::IS*S1* ribosome defect in binding intron RNA. The pull-down construct is expressed within small exons (E1 and E2, green), with the intron encoded protein (IEP, gray) expressed in *trans*. Intron RNA containing a streptavidin aptamer (SA, black) was captured using streptavidin resin (**1**), and the RNA was subjected to a Northern blot (**2**), probing for intron RNA and 16S rRNA. The ratio of rRNA per intron RNA was plotted, with individual replicates as data points, the mean as a horizontal line, and standard deviation as whiskers. Significantly more rRNA (p < 0.05) copurified with intron RNA in the control strain than the *rlmH*::IS*S1* mutant strain. Full blot is in Additional File 1: Fig. S18

Since ribosomes have been shown to interact with group II introns [16] and RlmH acts on fully assembled ribosomes [41], we probed the interactions between intron RNA and 70S ribosomes from both wild-type and *rlmH*::IS*S1* mutant strains. To this end, we measured binding between refolded in vitro-transcribed intron RNA and purified 70S ribosomes from *rlmH*::IS*S1* and wild-type cells using a gel-shift assay [16]. From these gels, we observed weaker binding with the *rlmH*::IS*S1* mutant ribosomes than the wild-type ribosomes, as indicated by less bound product relative to the unbound substrate at equivalent concentrations of ribosomes (Fig. 6a).

**Fig. 7:**
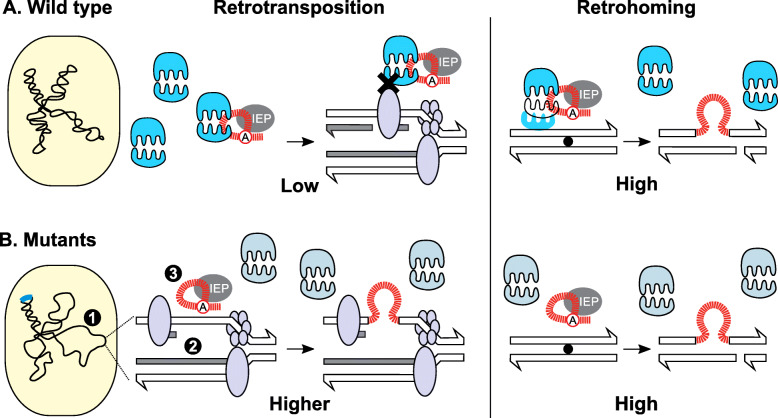
Model for protection of the genome against rampant retrotransposition. **a.** Wild-type host background. Under normal cellular conditions, the bacterial nucleoid is condensed, DNA replication is tightly controlled, and intron RNPs (red RNA lariat bound by gray intron-encoded protein) are bound by ribosomes (blue), which function to limit retrotransposition. Ribosome-bound RNPs encounter steric clashes (black X) with proteins at crowded DNA replication forks, inhibiting retrotransposition, with limited access to primers for cDNA synthesis (Low). In contrast, retrohoming into the homing site (black dot) in double-stranded DNA is unaffected by the presence of ribosomes with retrohoming being efficient, consistent with freedom from crowded replication forks (High). **b.** Mutant host backgrounds. Mutating certain host genes (blue insertion on the chromosome) increases retrotransposition frequency. (**1**) Disrupted amino acid biosynthesis pathways or misregulated stringent response (*spoT*::IS*S1*, *rpoE*::IS*S1*, *hisH*::IS*S1*) leads to restructuring of the nucleoid into a conformation more favorable for retrotransposition. (**2**) Stalled replication forks (increased single-stranded gaps) due to the activation of the stringent response or disruption of DNA recombination and repair functionalities (*coiA*::IS*S1*, *recT*::IS*S1*) become prime targets for retrotransposition. (**3**) Dysfunctional ribosome binding (*rlmH*::IS*S1*) allows intron RNPs to more freely invade DNA replication forks (Higher), whereas retrohoming remains unchanged (High). For illustrative purposes only, various effects of mutants (numbered **1**, **2**, and **3**) are shown together on the same figure

To further explore the role of ribosome-intron interactions in the *rlmH*::IS*S1* mutant, we performed a pull-down experiment to measure in vivo binding. We transformed plasmid-free *rlmH*::IS*S1* and wild-type *L. lactis* IL1403 with a plasmid containing the intron with a streptavidin-binding aptamer in the intron RNA sequence (Table 1) [26]. After growth, induction, and pull-down with streptavidin agarose resin, we isolated the RNA and performed a Northern blot probing for both intron RNA and ribosomal RNA (Fig. 6b; Additional File 1: Fig. S18). After normalizing the amount of copurifying 16S rRNA by the amount of pulled down intron RNA, we determined that the *rlmH*::IS*S1* mutant had significantly less rRNA per intron (0.28) compared to the control (0.48) on average (*p* < 0.05) (Fig. 6b). Thus, ribosomes from *rlmH*::IS*S1* do not bind intron RNA as well as wild-type ribosomes in vitro or in vivo. Taken together, the correlation of poor ribosome-intron binding with the specific increase in retrotransposition, but not retrohoming frequencies, suggests that ribosome binding can function to limit group II intron retrotransposition at replication forks (Fig. 7a).

## Discussion

By creating a library of mutants in *Lactococcus lactis* IL1403, the native host of the Ll.LtrB intron, we have underscored the importance of the genetic background of the host organism to group II intron retrotransposition. We narrowed down a list of 92 IS*S1* mutants that have elevated retrotransposition frequencies relative to the control, and further investigated a short list of 12 mutants on an individual basis (Fig. 4 and Table 2). From these 12 mutants involved in either amino acid biosynthesis, the stringent response, DNA recombination functions, and ribosome modifications, the *rlmH*::IS*S1* mutant, deficient in rRNA methylation, stood out as having the most dramatic and consistent retrotransposition phenotype. Additionally, the *rlmH::*IS*S1* mutant exhibited a defect in ribosome binding to the intron that leads us to the proposal that in vivo ribosome binding plays an important role in limiting intron retrotransposition in the native host.

### Take-home messages from the L. lactis mutant library

Although the mutant library is extensive, it is not comprehensive as we did not obtain complete coverage of the *L. lactis* genome. However, we did obtain a random, representative distribution (Fig. 1e). Based on our COG analysis, the functional distribution of our mutants is quite similar to the *L. lactis* genome, albeit functionalities related to translation (J) and nucleotide metabolism (F) are underrepresented in the library (Fig. 1f). Since IS*S1* transposition is random (Fig. 1E; Additional File 1: Fig. S4) [23, 28], we suspect that underrepresentation in the mutant library is a result of gene essentiality. In *Bacillus subtilis*, many genes involved in protein synthesis (J), as well as genes involved in nucleic acid metabolism (F) are known to be essential [44]. Following the same logic, we suspect the overrepresentation we observe in amino acid metabolism (E), carbohydrate metabolism (G), and host defense mechanisms (V) is due to the non-essential or redundant nature of some of these genes under laboratory growth conditions in rich media. Although the STRING protein interaction network analysis failed to reveal a master regulator of retrotransposition (Additional File 1: Fig. S9), the existence of such a regulator cannot be entirely ruled out due to the incomplete nature of our library.

When we compared mutants that exhibited increased retrotransposition against the entire library, we observed RTP-up mutants more frequently occurred in COG category E (Fig. 3), which contains genes with functions related to amino acid transport and metabolism [29]. The relationship between amino acid metabolism, specifically starvation and the stringent response, and retrotransposition has been previously described in *E. coli* [33]. Indeed, among the top-12 mutants of interest, a handful have functionalities involved in the stringent response and amino acid metabolism, namely *spoT*::IS*S1*, *hisH*::IS*S1*, *rpoE*::IS*S1*. In the *E. coli* work, ∆*relA* and ∆*spoT* mutants allowed us to conclude that the ppGpp-mediated stress response stimulates retrotransposition [33]. Since ppGpp is a key signaling molecule for starvation and the stringent response, it is unsurprising that other genes with functionality related to amino acid biosynthesis (E) also affect retrotransposition. Among the top-12 mutants, *hisH* is essential for biosynthesis of histidine [34]. The regulation of histidine biosynthesis extends to another mutant in our library, *rpoE*::IS*S1*, as RpoE is responsible for proper regulation of *hisH* [35]. Considering that these three top-12 mutants, *spoT*::IS*S1*, *hisH*::IS*S1*, and *rpoE*::IS*S1*, relate to amino acid starvation or regulation, we suspect that they all function to affect retrotransposition through a similar ppGpp-mediated mechanism to that reported in *E. coli* [33]. The research in *E. coli* posited that the stringent response, following amino acid starvation, stimulates retrotransposition by promoting a nucleoid structure that favors retrotransposition and increases the occurrence of stalled replication forks [33]. Despite the differences between host organisms, our data suggest that the importance of amino acid metabolism for regulation of retrotransposition is conserved in the native host (Fig. 7b).

Others of the top-12 RTP-up mutants, *oriC*::IS*S1*, *recT*::IS*S1*, and *coiA*::IS*S1*, disrupt genes or regions important for DNA replication, recombination, and repair. The *oriC*::IS*S1* insertion is at the origin of replication of the *L. lactis* chromosome, which is the location of initiation of DNA replication. We observed an increase in retrotransposition frequencies in *oriC*::IS*S1* (Fig. 4b), which could be attributed to stalled replication forks because of the IS*S1* insertion, but since the GFP assay indicated that this mutant exhibits dramatically increased expression regardless of induction (Fig. 4g; Additional File 1: Fig. S15), it may be that the retrotransposition increase is an indirect effect of improved expression of the intron construct. In contrast, *recT*::IS*S1* exhibits elevated retrotransposition with a normal expression profile compared to the control (Fig. 4b and g; Additional File 1: Fig. S10 and S15). The RecT protein is important for DNA double-strand break repair [37] and disrupting DNA repair may provide more opportunities for retrotransposition into damaged DNA or stalled replication forks, since group II introns interact directly with DNA replication fork machinery [45]. Although CoiA, which is represented by three independent insertion mutants, is matched to COG category R (general function prediction only), a closer search revealed that the CoiA protein plays a role in DNA processing following transformation by promoting recombination [36]. Similar to *recT*, it is possible that expression of *coiA* promotes recombination and inhibits retrotransposition by stimulating proper cellular repair pathways. Overall, these functions related to DNA replication, recombination, and repair, fall in line with group II intron retrotransposition pathways, where stalled replication forks and impaired repair or recombination functionality create single-stranded DNA foci that facilitate reverse splicing and second-strand DNA synthesis [6, 7, 46] (Fig. 7b).

### Ribosome methylation as a protective measure against rampant retrotransposition

The strong and consistent phenotype of the *rlmH*::IS*S1* mutant with 20-fold increased retrotransposition required our verifying the rRNA methylation defect in the mutant and eliminating the possibility that *rlmH*::IS*S1* acts directly by modifying intron RNA (Fig. 5d). Also, using genetic complementation, we showed that the phenotype is due directly to the *rlmH* mutation rather than to a pleiotropic effect (Fig. 4i). We therefore conclude that the rRNA methylation defect directly inhibits ribosome binding to intron RNA, which in turn relieves an inhibitory effect on retrotransposition (Fig. 6). These results are consistent with our previous studies that demonstrated strong and specific binding of 70S ribosomes to intron RNA in vitro and in vivo in *L. lactis* [16]. However, in *E. coli* ribosome binding actually accounts for an increase in retrotransposition, rather than protecting the genome from group II intron invasion [15, 16]. This difference is accounted for by *E. coli* ribosomes guarding group II introns from RNase E degradation, whereas *L. lactis* does not have a homolog for RNase E. Rather, *L. lactis* is more similar to *B. subtilis* in this respect and likely uses an entirely different degradosome than that used by *E. coli* [47]. Therefore, it appears that ribosome-intron interactions are playing a different role in the native host.

Importantly, our finding that retrohoming of *rlmH*::IS*S1* is *not* elevated, in contrast to retrotransposition, is consistent with the inhibitory effect of ribosome binding being exerted at the level of the DNA substrate, which is different for these two retromobility processes [6, 7, 11–13]. Retrotransposition occurs most frequently into crowded replication forks, whereas retrohoming occurs at a specific homing site that is free of complex molecular machinery and appears to have evolved for efficient endonuclease-mediated intron integration [6, 7, 11–13]. Thus, we propose that ribosome occupancy of group II introns would contribute to the steric clashes at the crowded replication fork that could hinder retrotransposition in the native host (Fig. 7a).

As rampant retrotransposition is dangerous for the host organism, ribosome-intron binding may have evolved to limit retrotransposition and thereby protect the native *L. lactis* host. Organisms from bacteria to mammals have evolved a wide arsenal of mechanisms to reduce the burden of retrotransposons and other mobile elements [48–51]. Expression of group II introns and their corresponding retrotransposition activity has a cost on both bacterial growth rate and survival [52]. To combat these defects, group II intron retrotransposition in *E. coli* is limited by the activity of RNase E [15]. Yet, *L. lactis* lacks any homologs of RNase E and has to cope with a higher retrotransposition frequency of the Ll.LtrB intron. Our results argue strongly that one mechanism for *L. lactis* to handle rampant retrotransposition is through ribosome-intron binding to inhibit retrotransposition at the crowded replication fork (Fig. 7).

Mobile elements are often a source of conflict within a genome, with various evolutionary pathways of resolution [50]. Although, we describe an example of how interactions between ribosomes and group II introns can limit retrotransposition to protect the host, these introns are not the only retrotransposons whose transposition is modulated by ribosomes: Alu elements, non-autonomous retrotransposons that are dependent on the protein activity of the autonomous LINE retrotransposons, can hijack and stall ribosomes translating LINEs in order to steal the ORF2 protein needed for Alu mobility [53, 54]. The intersection of these two retrotransposons at the level of the ribosome sets the stage for additional conflicts and/or control by the host organism. The interactions between retrotransposons and the ribosome have yet to be fully explored, but in light of our results with group II introns, we predict that ribosomes serve as a more general layer of regulation for retrotransposons.

## Conclusions

As demonstrated with a library of IS*S1* mutants, the biology of the native host is key for controlling group II intron retrotransposition. Some interactions appear to be conserved across organisms, such as the correlation between mutations in amino acid transport and metabolic functions with increased retrotransposition, suggesting that the activation of the stringent response may broadly stimulate retrotransposition. In contrast, group II intron-ribosome interactions behave distinctly in the native host, such that disruption of ribosome binding leads to a dramatic increase in retrotransposition frequency, without affecting retrohoming. Therefore, we have uncovered a novel role for ribosomes as guardians of the host genome, by limiting retrotransposition at replication forks, which may be broadly applicable to other retrotransposons.

## Materials and methods

### Bacterial growth conditions

Strains utilized in this study are listed in Table 1. All strains were grown in the conditions described as follows unless stated otherwise. *L. lactis* IL1403 strains were grown in GM17 media (M17 broth supplemented with 0.5% (w/v) glucose) in tightly capped tubes or bottles at 30 °C without aeration. Where appropriate, the media contained chloramphenicol at either 5 or 10 μg/mL (Cam_5_ or Cam_10_), kanamycin at 160 μg/mL (Kan_160_), erythromycin at 2 or 0.5 μg/mL (Erm_2_ or Erm_0.5_), or spectinomycin at 50 μg/mL (Spc_50_). *E. coli* strains were grown in Luria Broth (LB) media at 37 °C with aeration. Where appropriate, the media contained ampicillin at 100 μg/mL (Amp_100_), chloramphenicol at 25 μg/mL (Cam_25_), or erythromycin at 150 μg/mL (Erm_150_).

### Plasmid methodology, enzymes and oligonucleotides

Plasmids utilized in this study are listed in Table 1. Plasmid DNA was isolated and purified using EZNA Plasmid Mini kit (Omega). All oligonucleotides are listed in Additional File 1: Table S5; they were designed either by manual inspection or with NEBuilder (nebuilder.neb.com) and were synthesized by Integrated DNA Technologies (IDT). PCR fragments were amplified using either CloneAmp HiFi PCR Premix (Clontech) or Taq polymerase 2x Master Mix (Thermo Fisher Scientific). All cloning was performed in *E. coli*, before transformation into *L. lactis* strains.

To cure *L. lactis* IL1403 IS*S1* mutants, fresh 1 mL subcultures without antibiotic were started with 50 μL of inoculum and varying amounts of 50% ascorbic acid (0, 100 μL, 200 μL), and grown for 18 h. Cultures were diluted 10^2^- and 10^3^-fold, plated on GM17, and grown for 18 h. Colonies were patched on GM17 and GM17 Cam_10_ to identify sensitive (Cam^S^) colonies, which were further PCR-verified for the loss of plasmid (IDT5546 and IDT5547).

### Construction of the mutant library

The library of IS*S1* mutants was generated as described in [23]. In brief, *L. lactis* IL1403 carrying Ll.LtrB intron donor plasmid pLNRK-RIG was freshly transformed with pGh4:IS*S1* plasmid by electroporation. A colony of cotransformants was selected after overnight growth at 28 °C on GM17 Cam_10_ (selection for pLNRK-RIG) and Erm_5_ (selection for pGh4:IS*S1*) agar. To induce transposition of IS*S1* and integration of the plasmid into the bacterial chromosome, fresh cultures were incubated for 2.5 h at 28 °C followed by shift to 37.5 °C for another 2.5 h. Mutants with transposition events were selected by overnight growth at 37 °C on GM17 Cam_5_ and Erm_2_. Individual mutants were then grown in 96-well plates overnight at 37 °C in 1 mL of GM17 Cam_10_. To cure cells of pGh4:IS*S1*, overnight cultures were diluted 10^3^-fold in fresh GM17 Cam_10_ and incubated for 18 h at 28 °C. Then, cultures were streaked on GM17 Cam_5_ agar (selection for pLNRK-RIG) without Erm (no selection for pGh4:IS*S1*) and subjected to the final temperature shift to 37 °C for 18 h. Individual colonies were patched onto selective (Erm_2_) and non-selective plates (Cam_5_) and incubated at 37 °C. Sensitivity to Erm indicated loss of pGh4:IS*S1* plasmid.

### Hybridization probe preparation

The IS*S1*-specific probe was amplified with primer pair IDT3705 and IDT3706 using highly diluted pGh4:IS*S1* plasmid DNA as template (expected product 267 bp), and the RIG-specific probe was amplified with IDT374 and IDT375 using pLNRK-RIG as template (expected product 1545 bp). Resulting PCR products were purified by gel extraction and labeled with α-[^32^P]-dCTP (PerkinElmer) using the Random Primers DNA Labeling System (Invitrogen).

All oligonucleotide probes were labeled with 1–2 U of T4 polynucleotide kinase and 25 μM γ-[^32^P]-ATP (PerkinElmer) for 1 h at 37 °C followed by purification with Illustra MicroSpin G-50 columns (GE Healthcare). All experiments using ^32^P were imaged using the Typhoon Trio phosphor-imager (GE Healthcare).

### Colony blot hybridization

Hybond XL Membranes (GE Healthcare) were laid on top of agar plates with putative mutant colonies for 5 min for colony transfer. Following transfer, colonies were lysed by soaking the membranes in the following solutions: twice in 20 mg/mL lysozyme for 15 min at 37 °C, twice in 0.5 M NaOH for 3 min at 25 °C, twice in 1 M Tris-HCl (pH 7.4) for 5 min at 25 °C, and once in a buffer containing 1.5 M NaCl and 0.5 M Tris-HCl (pH 7.4) for 5 min at 25 °C. Membranes were crosslinked with a Stratalinker UV 1800 (Stratagene). Crosslinked membranes were prehybridized in Rapid Hybridization Buffer (GE Healthcare) for 15 min at 65 °C, and then incubated with denatured PCR probes for IS*S1* or RIG for 2 h at 65 °C. The blots were washed twice with 6X SSC for 15 min at 65 °C, followed by a final wash with 2X SSC for 15 min at 65 °C. The membranes were exposed to Storage Phosphor Screens for 1 day at 25 °C.

### Southern blotting and hybridization

Genomic DNA of selected IS*S1* mutants was isolated and purified using nexttec 1-Step DNA Isolation Kit for Bacteria (nexttec Biotechnolgie GmbH). Up to 3 μg of genomic DNA was digested with *Hind*III-HF (NEB). Approximately 400 ng of digested DNA was loaded on a 0.7% agarose gel in 0.5X TBE and run for 8 h at 25 °C. The gels were electroblotted for 30 min at 12 V onto Biodyne B-charged nylon membranes (Pall) using a GENIE electroblotter (Idea Scientific). The immobilized DNA was denatured by soaking the membranes in 0.4 N NaOH for 10 min, washed with 2X·SSC for 10 min and crosslinked as above. Prehybridization was conducted for 1 h at 65 °C in Rapid Hybridization Buffer (GE Healthcare). The [^32^P]-labeled IS*S1*-specific probe (PCR product of primers IDT3705 and IDT3706) was denatured with 0.1 vol 2 N NaOH for 8 min and then neutralized with 0.1 vol 1 M Tris–HCl, pH 7.4. The prehybridization buffer was replaced with 5 mL of fresh hybridization buffer with probe. Hybridization was conducted for 24 h at 65 °C. The blots were washed twice with 2X·SSC for 30 min at 65 °C, and once with 0.1X SSC and 0.1% SDS for 30 min at 65 °C. The membranes were then imaged as described above.

### Mapping of ISS1 insertion sites with next-generation sequencing

To minimize number of sequencing reactions, we used the Straight Three strategy for orthogonal pooling and mapping [27]. Briefly, all arrayed IS*S1* mutant strains were grown in 1 mL GM17 Cam_10_ until saturation. The collection of 11 plates was divided into two sets, PL1–6 and PL7–11. Pools were generated by combining cultures from common rows (A to H) and columns (1 to 12) across all plates in a set. All IS*S1* mutant strains from a plate were pooled to create plate pools, 6 plates in the PL1–6 set and 5 plates in PL7–11 set.

For each pool, genomic DNA was isolated using DNeasy Blood & Tissue Kit (Qiagen) following the manufacturer’s protocol for Gram-positive bacteria. DNA was assessed using electrophoresis on an agarose gel and NanoDrop (Thermo Fisher Scientific). The total DNA (2 μg in 100 μL final volume of TE buffer) was fragmented using a Bioruptor Standard Sonication System (Diagenode) with the following parameters: 30 min of 30 s on/off cycles at 4 °C. The sheared DNA was visualized on a 0.7% (w/v) agarose gel stained with ethidium bromide and fragments in the range of 200 bp - 800 bp were isolated from the gel with a QIAquick Gel Extraction Kit (Qiagen). Preparation of genomic DNA was followed by library construction using the NEB Next DNA Library Prep Kit (NEB). Purification of the fragments when necessary was performed with QIAquick PCR Purification Kit (Qiagen). Samples were submitted for Illumina sequencing at the Center for Functional Genomics at UAlbany for high output (75 cycles, 400 M reads) sequencing.

The adapter and IS*S1* sequences were removed from reads using a custom biopython script (available on request). The script allows precise detection of the 3′ end of the IS*S1* fragment within the generated reads followed by the trimming of the IS*S1* and the upstream sequence leaving the flanking fragment. The 5 bp at the 3′ end of the reads appeared to be of low quality and were removed as well. The resulting 25-bp reads were analyzed further using the Galaxy server (usegalaxy.org) [55]. Bowtie software was used for mapping of the reads under custom settings [56], as follows: ‘Maximum number of mismatches permitted in the seed’ was set as ‘0’ (parameter -n), and ‘Whether or not to try as hard as possible to find valid alignments when they exist’ was set to ‘Try hard’ (parameter -y). Additionally, the setting was to report the ‘best’ singleton alignments in terms of stratum (the number of mismatches) and in terms of the quality values at the mismatched positions (parameter –best). FASTQ Groomer [57], SAMtools [58], deepTools [59], and BEDTools [60] were also used for analysis among other bioinformatic tools implemented in the Galaxy server (usegalaxy.org) [55].

### Clusters of orthologous genes (COG) analysis

All annotated proteins from *L. lactis* IL1403 (ASM686v1) were used as a query in a local blastp [61] search against the COG database [29] (last accessed July 10, 2018), where the best match for each query was recorded, based on the E-value scores. If a protein/COG family hit was represented in multiple categories, each category was counted once. The relative abundance of categories was used for comparisons. For statistical comparisons, we performed a hypergeometric test to compare our test sample against background levels (as performed in [62, 63]). To calculate *p*-values, we used the phyper function in the statistical software R and adjusted the values to correspond to a two-tailed test in order to be appropriately conservative for the comparisons made. This same analysis was performed to compare RTP-up mutants against the library.

### Retrotransposition and retrohoming assays

Retrotransposition assays in *L. lactis* IL1403 were performed with the intron donor plasmid pLNRK-RIG in both high-throughput and a more quantitative low-throughput manner. High-throughput retrotransposition (HTP-RTP) assays were performed on all 1006 mutants simultaneously in 96-deepwell plates. Overnight cultures from the original mutant stocks were grown in 1 mL of GM17 Cam_10_ then subcultured 1∶10 in fresh GM17 Cam_10_ until the control strain IL1403 pLNRK-RIG reached OD_600_ of 0.2. Intron expression was induced by addition of 10 ng/mL nisin and cultures were grown for an additional 3 h until spot plating (5 μL) on rectangular GM17 Kan_160_ plates using a robotic liquid handler JANUS G3 (PerkinElmer) equipped with an 8-tip Varispan arm (PerkinElmer). Plates were incubated at 30 °C for 2 days.

Robustness of growth (spot size) on GM17 Kan_160_ for each strain was analyzed with SGAtools (sgatools.ccbr.utoronto.ca) [30]. Spot-size scores for each plate were normalized to those calculated for control IL1403 pLNRK-RIG from the same plate, resulting in relative intron retrotransposition levels for each strain. To identify mutant strains showing consistently higher relative retrotransposition, box plot analysis was performed using BoxPlotR (shiny.chemgrid.org/boxplotr) followed by sorting with custom Python scripts (available by request). Mutants of interest were sorted into two tiers. Strains exhibiting relative retrotransposition levels between the third quartile (Q3) and fourth quartile (Q4 or maximum) in all three HTP-RTP assays were assigned to the first tier. Strains that placed between Q3 and Q4 twice and between the median (Q2) and Q3 once were identified as second tier.

Quantitative low-throughput RTP assays were performed in selected strains as previously described [6, 21]. In brief, cultures were grown in 40 mL GM17 Cam_10_ with a 1:33 dilution to an OD_600_ of 0.2. Intron expression was induced with 10 ng/mL nisin for 3 h and plated on GM17 and GM17 Kan_160_. Remaining cultures were pelleted in 10 mL increments and stored at − 80 °C for further analyses (see below).

Retrohoming assays, using pMN1343 to provide the homing site, were performed as in [6], except that the pLNRK-RIG intron donor plasmid was used and induction was performed for 3 h, consistent with the rest of this study. Retrohoming frequency was calculated as for retrotransposition frequency and significance was assessed with a t-test.

### Mapping ISS1 insertion sites of RTP-up mutants with inverse PCR

Genomic DNA of selected IS*S1* mutants was isolated and purified using nexttec 1-Step DNA Isolation Kit for Bacteria (nexttec Biotechnolgie GmbH). DNA (1 μg) was digested with *Sau*3AI and circularized by ligation with T4 DNA ligase (NEB), according to manufacturer’s protocols. The DNA fragments were amplified with primers IDT3858 and IDT3859 or with primers IDT3860 and IDT3861 to obtain products for 5′- or 3′- IS*S1* flanking regions, respectively. Resulting PCR fragments were visualized by electrophoresis on 1% (w/v) agarose gels, gel purified and sequenced (EtonBio). Sequences were mapped to the genome of *L. lactis* IL1403 [22] using BLAST (blastn) [64] accessed through the National Center for Biotechnology Information (NCBI; www.ncbi.nlm.nih.gov). The accession number of the reference genome nucleotide sequence is NC_002662. Mutant identification was verified by PCR with gene-specific primers (Table S5).

### Protein interaction network

Unique protein sequences were retrieved from NCBI for all nonredundant mutants in the library (510 sequences). These sequences were queried against the STRING database (string-db.org) [32] and 505 of the submitted 510 sequences were mapped to the STRING database. The remaining 5 sequences not identified in the STRING database are likely new annotations in the *L. lactis* genome, which was reannotated on March 22, 2020. Additionally, two sequences belonging to transposases were identical, despite having different names, and therefore were consolidated by STRING, resulting in 503 sequences. The mapped STRING identifiers were used to generate a network with the STRING app (version 1.5.1) [65] in Cytoscape (version 3.7.2) [66]. COG categories were added to each mutant and the network was restructured using a grouped layout based on COG category. When a protein belonged to multiple COG categories, a single category was chosen arbitrarily for grouping purposes. The final network contained 503 nodes, 52 of which had no interactions given the default parameters cutoff confidence score (0.4).

### Dot-blot hybridization for plasmid quantification

To measure plasmid donor (pLNRK-RIG) copy number, cell pellets generated during the low-throughput RTP assay were re-suspended in 2 mL of GM17 and diluted to OD_600_ of 0.4. For each sample, 2 μL was spotted onto Hybond XL membrane (GE Healthcare) and air dried. The membrane was treated as described above for colony blot hybridization. After crosslinking, membranes were prehybridized in Rapid Hybridization Buffer (GE Healthcare) for 15 min at 42 °C, and then incubated with a 5′-labeled [^32^P]- pLNRK-RIG specific oligonucleotide probe (IDT5059), for 1 h at 42 °C. The membranes were washed three times with 6X SSC for 15 min at 42 °C, and Phosphor images generated, as described above, were analyzed using ImageJ (imagej.nih.gov/ij) [67].

### RNA quantification with northern blotting

Total RNA was isolated from cell pellets with a phenol/chloroform isoamyl alcohol (PCIA) extraction, ethanol precipitation and DNase treatment (RQ1 DNase, Promega), followed by an additional PCIA extraction and a final ethanol precipitation. Northern blotting was performed as previously described [16, 26]. Hybridization was with 5′-labeled [^32^P]-intron-specific oligonucleotide probe (IDT1073) for 3 h at 42 °C. To normalize, blots were stripped and hybridized with the 5′-labeled [^32^P]-16S rRNA-specific oligonucleotide probe (IDT861) as described above.

### Measuring splicing efficiency by primer-extension analysis

Primer extension assays were performed as previously described [5, 26, 68]. Reverse transcription of 3 μg of DNase-treated total RNA was performed using SuperScript III (Invitrogen) and 0.4 pmol of 5′-labeled [^32^P]-intron-specific oligonucleotide (IDT1073). Reactions were separated on an 8% urea-polyacrylamide gel (National Diagnostics) and exposed on a phosphor screen. Analysis was performed using ImageQuant.

### Protein quantification by Western blotting

For LtrA analysis, Western blotting was carried out as described [16, 26] with modifications. Total cell lysates from low-throughput RTP assay cell pellets were run on a pre-cast 12% polyacrylamide mini-PROTEAN TGX gel (BioRad) at 300 V for 30 min and transferred onto PVDF membrane utilizing BioRad’s Trans-Blot Turbo Transfer System. The membrane was blocked with 10% dry milk, washed, and then incubated with anti-LtrA primary antibody (Covance) at 25 °C for 1 h. The membrane was then washed, incubated with secondary antibody (HRP conjugated, anti-Rabbit (Advansta)) at 25 °C for 1 h, washed again, and protein was detected using Advansta WesternBright Quantum reagents. Membranes were imaged and analyzed on BioRad Chemi Doc XR System. All samples were normalized to total protein per lane on a Coomassie-stained pre-cast gel.

### GFP assay

The pLNRK-GFP plasmid was generated by removing the intron from pLNRK-RIG via *Pst*I and *Spe*I digestion, followed by the ligation of the green fluorescent protein (GFP) gene that was amplified from pHGSap [69] using NEBuilder (IDT6054 and IDT6055). Individual mutants were cured, as described above, and transformed with pLNRK-GFP by electrotransformation, followed by PCR confirmation (IDT6056 and IDT6057). Plasmid-bearing strains were grown and subcultured 1:10 in 40 mL of GM17 Cam_10_. After 2 h, the cultures were split into two tubes. Half of the cultures were induced for GFP with 10 ng/mL nisin for 4 h, the others remained uninduced, and growth continued. One mL taken every hour was transferred to a 96-deepwell plate (Eppendorf), centrifuged at 3220 x g for 8 min, washed, and serial diluted in 1X PBS (137 mM NaCl, 2.7 mM KCl, 10 mM Na_2_HPO_4_, 1.8 mM KH_2_PO_4_). Volumes of 200 μL were transferred to a 96-well plate (clear flat bottom, Corning) to measure OD_600_ and GFP fluorescence (black flat bottom, Corning) on a plate reader (BioTek Synergy H1). Data were analyzed using BioTek Gen5 and Microsoft Excel.

### Complementation retrotransposition assays

To prepare the complementation vector pGh5:P_nisA_, IS*S1* was digested out of the pG^+^host5:IS*S1 L. lactis/E. coli* shuttle vector [23] using *Hind*III cut sites, resulting in pG^+^host5 (pGh5). The nisin-inducible promoter, P_nisA,_ was PCR-amplified from pLNRK-RIG (IDT6871 and IDT 6872) and sub-cloned into the pGEM-T Vector System I (Promega), transformed into *E. coli* DH5α, and subsequently digested and cloned into the *EcoR*I and *Hind*III sites of pGh5, resulting in “empty” pGh5:P_nisA_ vector. The *rlmH* gene was PCR amplified from *L. lactis* IL1403 gDNA (IDT6924 and IDT6925) and cloned into pGh5:P_nisA_ using NEBuilder, resulting in pGh5:P_nisA_ *rlmH*. Constructs were sequenced (Eton Bio) using M13 primers and transformed into the relevant *L. lactis* IL1403 strains. Wild-type and IS*S1* mutant strains containing pGh5:P_nisA_ or pGh5:P_nisA_ *rlmH* plasmids were grown in GM17 Cam_5_ Erm_0.5_ and retrotransposition assays were performed as described above but at Cam_5_. Statistical significance was assessed with a t-test.

### Ribosome purification, binding assays, and 70S rRNA isolation

Isolation of 70S ribosomes from *L. lactis* IL1403 and the *rlmH*::IS*S1* mutant was as [16] with minor modifications. Briefly, lysate was loaded on a 40% sucrose cushion (40% sucrose, 20 mM Tris-HCl pH 7.5, 500 mM NH_4_Cl, 0.5 mM EDTA, 6 mM β-mercaptoethanol, 20 mM MgCl_2_) and samples were pelleted by ultra-centrifugation at 29,500 rpm for 26 h using a Type 70 Ti rotor (Beckman Coulter). Ribosome pellets were resuspended in 70S resuspension buffer (50 mM NH_4_Cl, 20 mM MgCl_2_, 20 mM Tris-HCl pH 7.5, 0.5 mM EDTA, 6 mM β-mercaptoethanol), and then separated on a 10–40% sucrose gradient. Fractions containing 70S ribosomes were pooled, concentrated, and buffer-exchanged (3X) into final resuspension buffer (20 mM Tris-HCl pH 7.4, 200 mM KCl, 5 mM MgCl_2_). Purified ribosomes were used in ribosome binding assays with labeled in vitro-transcribed intron RNA as described [16] and data were plotted using the Prism 8 (GraphPad) software.

RNA samples for MS analysis were prepared from isolated 70S ribosomes by using PCIA extraction, followed by ethanol precipitation with MS-grade ammonium acetate, and resuspended in MS-grade water. Samples were digested with exonucleases as described below.

### In vivo intron RNA pull-down

Wild-type and *rlmH*::IS*S1* strains containing the pull-down plasmid pLNRK-SA were subcultured 1:20 into 100 mL GM17 Cam_10_, grown to an OD_600_ of 0.5 and induced for 3 h with nisin. Following induction, 50 mL aliquots of cell culture were collected by centrifugation and pellets were stored at − 80 °C. Cell pellets were resuspended in 300 μL CB_500_ buffer (20 mM Tris-HCl pH 8.0, 500 mM NaCl, 0.1 mM EDTA) and lysed by sonication. High Capacity Streptavidin Agarose Resin (Thermo Scientific) was washed 3 times with CB_500_ and then incubated with lysate overnight, nutating at 4 °C. The resin was then washed 10 times with CB_500_, followed by elution with 500 μL of 10 mM biotin in CB_500_. RNA was isolated from eluate with two rounds of PCIA extraction followed by ethanol precipitation. Northern blots were performed on isolated RNA as described above (1 h hybridizations), with identical gels run side-by-side to perform a single blot for each probe (IDT1073 for intron RNA and IDT861 for 16S rRNA). Statistical significance was assessed with a t-test.

### Native intron purification for MS analysis of RNA modifications

Ll.LtrB RNP induction and purification were performed as previously described [5], with the following modifications to obtain RNA for MS. After elution from the chitin column, RNA was isolated by PCIA extraction, followed by ethanol precipitation and DNase digestion. The control in vitro-transcribed intron RNA was synthesized as previously described [16]. All samples were separated on a 1% agarose gel in 0.5X TBE and bands corresponding to intron RNA and 16S rRNA were separately excised. Excised bands were frozen at − 80 °C overnight, then centrifuged at maximum speed for 30 min at 4 °C, and the supernatant was taken and the RNA was concentrated and buffer exchanged 3X into 10 mM ammonium acetate using a 3 kDa MWCO Amicon centrifuge column (pre-rinsed with 10 mM ammonium acetate).

### Digestion and MS analysis

Aliquots of 1.2 μg of RNA (in 30 μL in 10 mM ammonium acetate) were incubated with 1 μL Nuclease P1 (Sigma Aldrich) for 2 h at 37 °C. Ammonium bicarbonate was added to a final concentration of 100 mM, followed by treatment with 1 μL snake-venom phosphodiesterase (Sigma Aldrich) for 2 h at 37 °C to obtain the desired nucleotide monophosphate (NMP) mixtures for MS [70], after which samples were stored at − 20 °C to halt the reaction. Immediately before analysis, the NMP mixtures were diluted to 4 ng/μL in 10 mM LC-MS grade ammonium acetate (Sigma Aldrich) and 10% 2-propanol (FisherScientific). All samples were analyzed on a Thermo Scientific LTQ-Orbitrap Velos instrument [43, 71]. Nanoflow direct-infusion electrospray ionization (nanospray) was performed in negative ion mode using quartz emitters produced in house. Up to 5 μL of samples were loaded into each emitter with a gel loader pipette tip. A stainless-steel wire was inserted in the back-end of the emitter to supply an ionizing voltage that ranged between 0.8 and 1.0 kV. Source temperature and desolvation conditions were adjusted by closely monitoring the incidence of ammonium adducts and water clusters.

The relative abundance of each modification was expressed as Abundance versus Proxy (AvP), which was calculated according to the following equation:

$$ {AvP}_x=\frac{ai_x}{\sum_1^4{cr}_i}\ x\ 100 $$

in which the signal intensity (ai_x_) of each modification was normalized against the sum of the intensities displayed in the same spectrum by the four canonical bases (cr_i_).

Chemically synthesized methylated uridine and pseudouridine nucleosides were purchased from Berry & Associates and used to synthesize the corresponding NMPs, which were further purified as described in [72]. To reveal the unique fragmentation pattern of each isobaric NMP upon fragmentation in the gas phase, tandem mass spectrometry (MS/MS) analysis was carried out in both positive and negative ion mode as previously described [43, 73]. Then, the fragmentation pattern of isolated m/z for methylated uridine and pseudouridine from biological samples was compared to that of the synthetic standards to determine the composition of isobaric modified NMPs in the biological samples.

## Supplementary Information

**Additional File 1: Supplementary Figures and Tables**. This file contains **Supplementary Figures. S1-S18** and **Supplementary Tables S1-S5**.

**Additional File 2:.** IS*S1* insertion mapping. This file contains coordinates and gene information on the IS*S1* mutants generated in this study.

**Additional File 3:.** RTP-up mutants list and summary data. This file lists all verified single-insertion RTP-up mutants, with data on their insertion sites, mutant tier ranking, and COG classification.

## Data Availability

The datasets used and/or analyzed during the current study are available from the corresponding author upon request. Publicly available datasets used in this work are available in the COG database, http://www.ncbi.nlm.nih.gov/COG [29] and STRING database, http://string-db.org/ [32].
