# Supplementary material for: Methylation of rRNA as a host defense against rampant group II intron retrotransposition
Source: Mob DNA. 2021 Mar 7;12:9. doi: 10.1186/s13100-021-00237-z (PMC7938551; doi:10.1186/s13100-021-00237-z)
Supplement: Supplementary file 1 — Additional File 1: Supplementary Figures and Tables. This file contains Supplementary Figures. S1-S18 and Supplementary Tables S1-S5. [file 13100_2021_237_MOESM1_ESM.pdf]

# Methylation of rRNA as a host defense against rampant group II intron retrotransposition

**Authors:** Justin M. Waldern[1], Dorie Smith[1], Carol Lyn Piazza[1], E. Jake Bailey[1], Nicholas J. Schiraldi[2], Reza Nemati[3,4], Dan Fabris[1,3,5], Marlene Belfort[*,1,6], Olga Novikova[*,1,7]

**Affiliations:**

[1]Department of Biological Sciences and RNA Institute, University at Albany, 1400 Washington Avenue, Albany, NY 12222

[2]Academic and Research Computing Center, Information Technology Services, University at Albany, 1400 Washington Avenue, Albany, NY 12222

[3] Department of Chemistry, University at Albany, 1400 Washington Avenue, Albany, NY 12222

[4] Current address: Biogen, 125 Broadway, Cambridge, MA 02142

[5] Current address: Department of Chemistry, University of Connecticut, 55 N. Eagleville Road, Storrs, CT 06268

[6]Department of Biomedical Sciences, School of Public Health, University at Albany, 1400 Washington Avenue, Albany, NY 12222

[7]Current address: Biology Department, SUNY Buffalo State College, 1300 Elmwood Avenue, Buffalo, NY 14222

**Email addresses:**

[*]Corresponding authors:

Olga Novikova, novikoos@buffalostate.edu

Marlene Belfort, mbelfort@albany.edu

Co-authors:

Justin Waldern, jwaldern@albany.edu

Dorie Smith, dsmith7@albany.edu

Carol Lyn Piazza, cpiazza@albany.edu

E. Jake Bailey, elginjbailey@gmail.com

Nicholas J. Schiraldi, nschiraldi@albany.edu

Reza Nemati, reza.nematy@gmail.com

Dan Fabris, dan.fabris@uconn.edu

## Supplementary Figure Legends

**Figure S1.** IS*S1* mutagenesis schematic. The control strain *L. lactis* IL1403 containing the intron donor plasmid pLNRK-RIG was transformed with the pGh4:IS*S1* plasmid. The pGh4:IS*S1* plasmid contains a temperature sensitive origin of replication (Ts), the IS*S1* transposon and a selectable marker for Erythromycin resistance ($erm^R$). Transformants were cultured in fresh media, selecting for the intron donor plasmid, for 2.5 h at a low temperature (28°C) to allow for replicative transposition of the IS*S1* transposon. The temperature was then shifted to 37.5°C for 2.5 h to stimulate the loss of free pGh4:IS*S1* vector plasmid. The culture was then plated to select for IS*S1* integrants on GM17 $Cam_5$ $Erm_2$ plates and colonies were grown in fresh cultures at 37°C, arrayed into 96-well plates. The potential integrants went through a series of growth steps to stimulate vector excision (18 h at 28°C), and then to stimulate the loss of free vector (18 h at 37°C). The putative mutants went through a series of patching, verification with PCR and colony lift hybridization. This entire process was repeated to create an entire library of mutants

stored in 96-well plates, resulting in 11 plates of IS*S1* mutants containing the donor plasmid pLNRK-RIG.

**Figure S2.** Southern blots of random mutants. **A.** Southern blot was performed on a random subset of mutants in order to determine how often the mutagenesis process creates a single-insertion mutant (top). Blue dots indicate multiple IS*S1* insertions, whereas orange indicate that the presence of an IS*S1* insertion could not be resolved, such as through incomplete genomic DNA digestion. Bottom shows the corresponding DNA agarose gels of the restriction digests (*Hind*III) of genomic DNA used for Southern blotting. **B.** Mutant stability. Southern blots following a subset of IS*S1* mutants over multiple generations (G0-G12), showing the stability of IS*S1* mutants. Most mutants show identical banding patterns from G0 to G12, with only one mutant (PL7G11) showing a loss of IS*S1* over time, going from multiple insertions to a single insertion. None of the cultures tested showed more insertions over time.

**Figure S3.** Illumina sequencing schematic. The schematic details the multiplexed Illumina sequencing strategy of the IS*S1* mutant library. Cultures were grown in eleven 96-well plates and then pooled following the Straight Three strategy (Dale et al. 2018), resulting in 16 pools of rows, 24 pools of columns, and 11 pools of plates. Genomic DNA was isolated from these pools for Illumina sequencing. To generate a DNA library for sequencing, IS*S1* junctions were amplified using standard adapters P5A and P7A, which contain Illumina flow-cell binding sites and an adapter targeting the IS*S1* transposon (PCR1). Additional PCR amplification (PCR2) was performed with primers containing index sequences for data analysis.

**Figure S4.** IS*S1* insertion site sequence logo. Representation of the preferred insertion site of IS*S1* within the mutant library, where the size of each letter represents the relative frequency of a given nucleotide.

**Figure S5.** High-throughput RTP assay. **A.** Schematic of the experimental approach for the HTP-RTP assay. The assay was performed in 96-well plates using a liquid handling robot, plating on kanamycin to select for retrotransposition events. Plates were imaged and relative growth was scored into heatmaps using SGATools (Wagih et al. 2013). These heatmaps were used to create boxplots which enabled identification of mutants with consistently elevated retrotransposition compared to the control wild-type IL1403 pLNRK-RIG (white oval). Additional controls included the same strain with pRS01 with an "up" phenotype (Novikova et al. 2014) (pink oval) and a blank well containing only media (black oval). **B.** Full data from the HTP-RTP assay. Plates are shown alongside heatmaps, with boxplots on the right. Controls are boxed with a white outline on the plates and marked with a bracket on the heatmaps. Empty white cutouts in RTP3 are sections of the plate that were contaminated. The top-12 mutants selected for follow up analysis are indicated with white numbers in red boxes.

**Figure S6.** Southern blots of RTP-up mutants. Southern blots probing for IS*S1* for verification of RTP-up mutants. Mutants are labeled by plate (PL), row (letter, e.g. A) and column (number, e.g. 7). Below each blot is the corresponding DNA agarose gel showing the digestion (except for the bottom row of blots). Mutants with multiple insertions (blue dots) or where we were unable to resolve a single IS*S1* band (orange dots) were omitted from further analysis. The top-12 mutants are indicated with white numbers in red boxes, corresponding to Table 2.

**Figure S7.** Inverse PCR of mutants. **A.** Schematic representation of the inverse PCR protocol. Mutant cultures were grown, DNA isolated, partially digested with *Sau*3AI, and self-ligated to produce circular DNA. PCR was then performed outwards from IS*S1* (blue, with primers P1 and P2) to amplify the regions flanking the IS*S1* transposon (green and pink in 1 and 2 respectively). Bands corresponding to PCR products were excised from the gels, subjected to sequencing and mapped back to the IL1403 genome. **B.** Gels showing representative inverse PCR products. The most prominent band (indicated with a black arrowhead) was extracted and sent off for sequencing analysis. When there were multiple major bands, each band was extracted.

**Figure S8.** Verification PCR gel. Gel showing verification PCR results of the top-12 mutants. Primers were designed to flank the host gene, such that an IS*S1* insertion would result in a large product around 1-1.5 kb.

**Figure S9.** STRING protein interaction network. Each node (circles or numbered squares) represents a different protein from the mutant library, where red nodes represent RTP-up mutants. Each edge (line) between nodes represents an interaction between these proteins as identified in the STRING database. Nodes are clustered based on COG category, which is identified by a corresponding letter (Additional File 1: Table S3). COG categories are grouped based on greater functionality (i.e. cellular processing and signaling, information storage and processing, and metabolism). The unlabeled cluster (bottom right) contains proteins that did not have a match in the COG database. Red squares represent the top-12 RTP-up mutants, excluding *oriC*::IS*S1*, which does not encode a protein. The number of the square corresponds to the key of

RTP top-12 mutants (Table 2). The *coiA::*IS*S1* mutant has three insertion sites and therefore has 3 numeric identifiers (in COG category R).

**Figure S10.** Retrotransposition frequency relative to control. Plot showing all data for the retrotransposition frequency of the top-12 mutants, relative to the control wild-type IL1403 harboring pLNRK-RIG. These data without extreme outliers are shown in Figure 4B.

**Figure S11.** Characterization: dot blot hybridization of plasmid DNA. All plasmid copy number data for the top-12 mutants, measured using dot blot hybridization are indicated by white numbers in red boxes, and are labeled with the gene name. Values below each image are the quantification relative to the control, wild-type IL1403 harboring pLNRK-RIG.

**Figure S12.** Characterization: intron RNA quantitation by Northern blots. The top-12 mutants are indicated by white numbers in red boxes, and are labeled with the gene name. From each set, the top images are blots probing for intron, middle images are blots probing for 16S rRNA, and bottom images are the agarose gel used for transfer. Values below each image are the quantification of intron RNA, normalized to 16S rRNA, relative to the control wild-type IL1403 harboring pLNRK-RIG.

**Figure S13.** Characterization: splicing quantitation by primer extension analysis. All intron splicing data for top-12 mutants, measured using primer extension assays are indicated by white numbers in red boxes, and are labeled with the gene name. Values below each image are the splicing efficiency relative to the wild-type IL1403 harboring pLNRK-RIG. Splicing efficiency

is calculated by the amount of spliced intron (SI) divided by the sum of spliced intron plus precursor (P).

**Figure S14.** Characterization: intron-encoded protein level by Western blots. Intron-encoded protein (LtrA) abundance for the top-12 mutants was measured with an antibody specific to the LtrA protein. Below each Western blot is the corresponding SDS-PAGE gel, stained with Coomassie for total protein. Top-12 mutants are indicated by white numbers in red boxes, and are labeled with the gene name. Values below each image are the quantification relative to the wild-type IL1403 harboring pLNRK-RIG. Values are measured as the abundance of LtrA normalized to total protein, relative to control.

**Figure S15.** Induction from the $P_{nisA}$ promoter**.** GFP fluorescence over time for each of the top-12 mutants is shown. Cultures were split and induced (green) or left uninduced (gray) after two hours of growth (T2) and GFP fluorescence was measured each hour. Points are averaged GFP fluorescence, relative to wild-type control, with the shaded area representing standard deviation. Both *rsmE*::IS*S1* and *hisH*::IS*S1* were omitted from this analysis, since the donor plasmid could not be cured and the reporter plasmid could not be transformed, respectively.

**Figure S16.** Tandem MS/MS analysis of methylated uridine/pseudouridine standards. The data were obtained by activating the gas-phase fragmentation of chemically synthesized standards of m3Ψ isobars. All standards shared the same elemental composition and, thus, generated precursor ions at the same 337 m/z. However, the different positions of the substituent methyl group promoted different fragmentation pathways. Dashed arrows depict where a standard

fragmented into a readily identifiable major product ion. In the case of m3Ψ , the process led to the formation of a unique characteristic fragment at 239 m/z, which was absent in all other isobars examined.

**Figure S17.** Preparation of group II intron RNA samples for MS analysis. Intron RNA was purified through gel extraction from a native agarose gel. Depicted is the tandem expression of the intron RNA and the intron-encoded protein (IEP). The intron RNA is expressed as a lariat (red), with the branchpoint adenosine marked by an encircled A. The intron-encoded protein (IEP, gray) is expressed as a fusion with an intein (blue "I") and a chitin binding domain (orange "CBD"). Intron RNPs were affinity purified using a chitin column, with tag removal and elution performed by DTT treatment. RNA was isolated and then run on a native agarose gel, which enabled separation of the intron RNA from the 16S rRNA. Gel-extracted RNA was digested to mononucleotides with RNases and then subjected to MS analysis, which enabled the identification and quantification of RNA modifications from isolated intron RNA, isolated 16S RNA and *in vitro*-transcribed control RNA.

**Figure S18.** Intron RNA pull-down blots. Northern blots of RNA pulled down based on the streptavidin intron construct (pLNRK-SA in Table 1). Blots performed in parallel were probed for 16S rRNA and intron RNA. The top distinct band of the 16S blot was quantified for analysis.

## Supplementary Figures

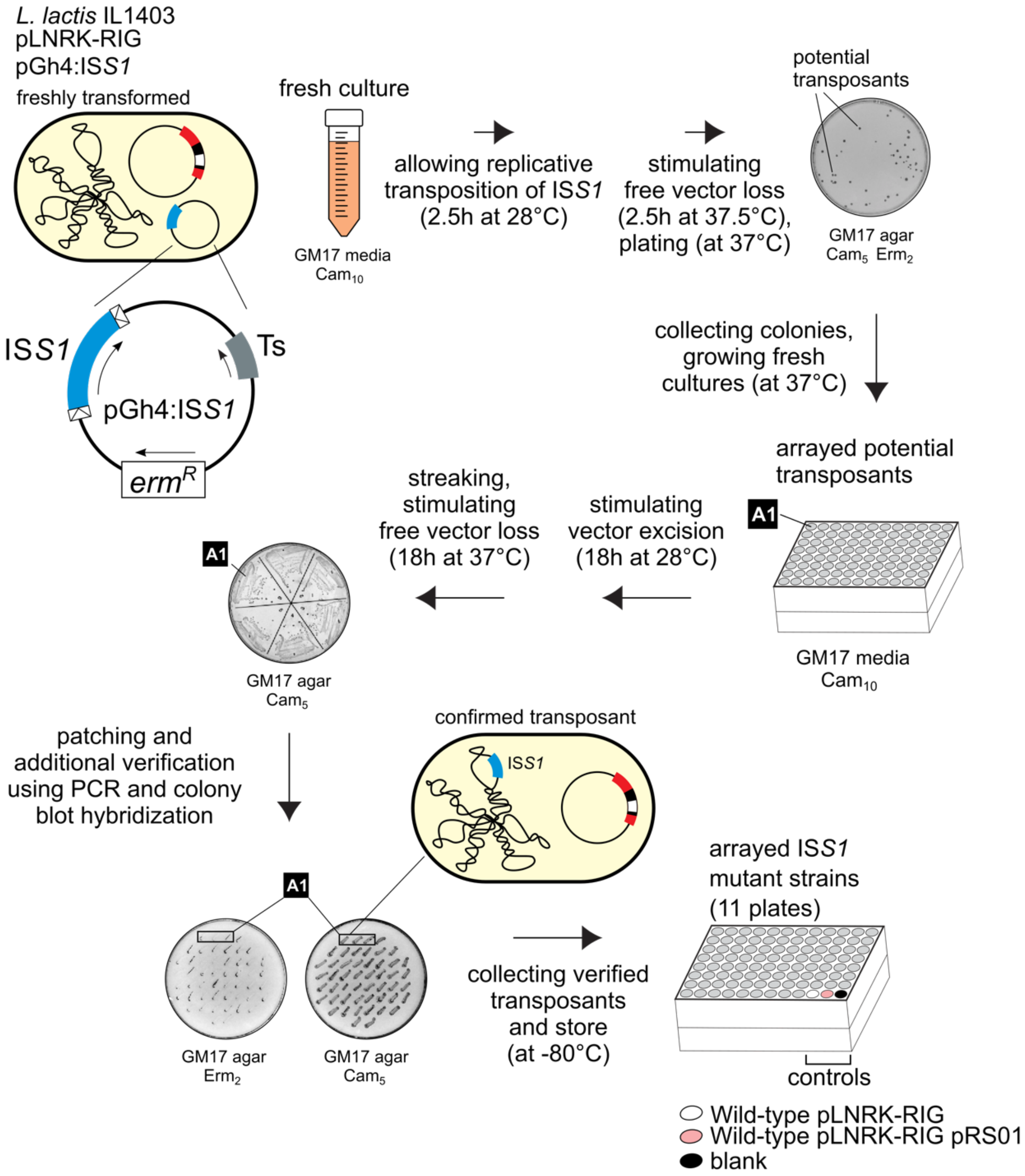


**Figure S1.** IS*S1* mutagenesis schematic

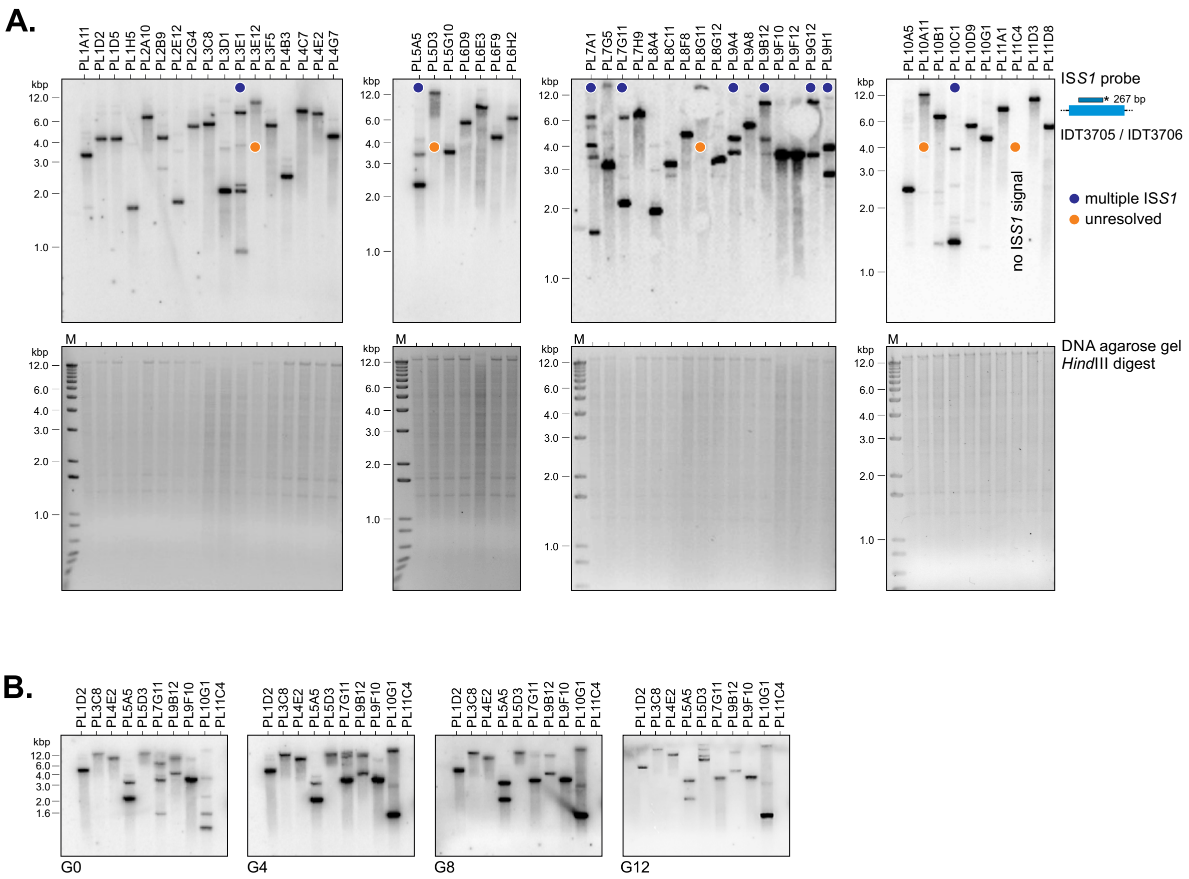


**Figure S2.** Southern blots of random mutants

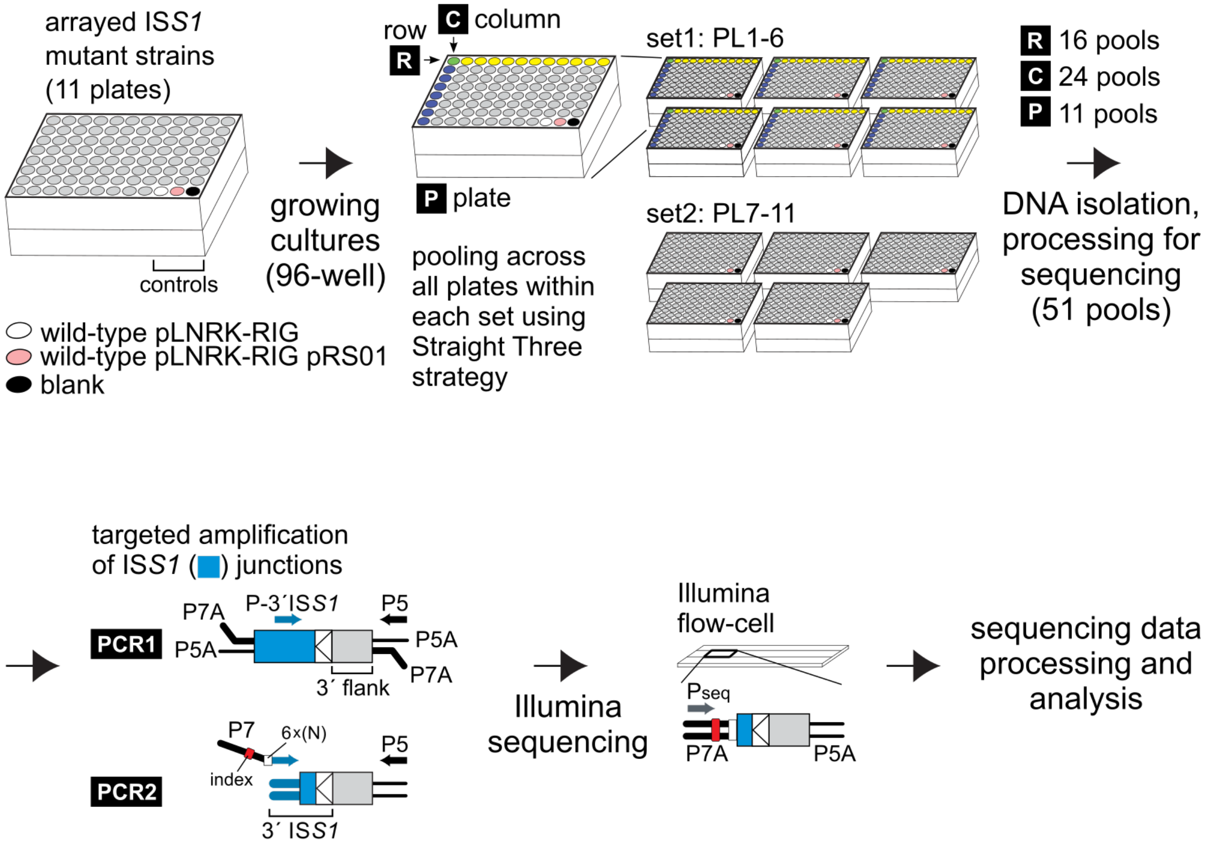


**Figure S3.** Illumina sequencing schematic

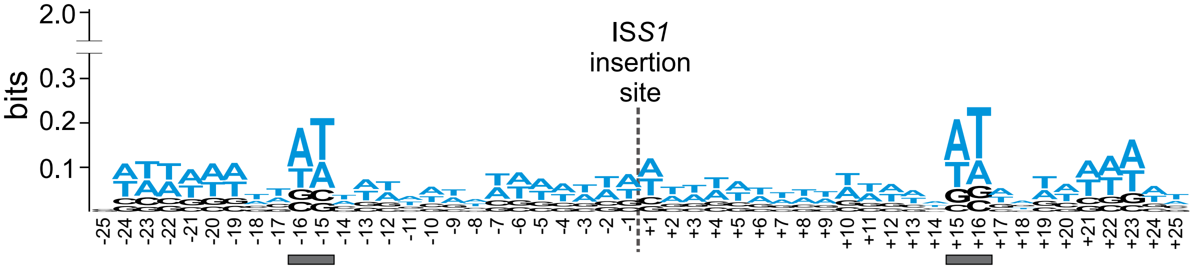


**Figure S4.** IS*S1* insertion site sequence logo

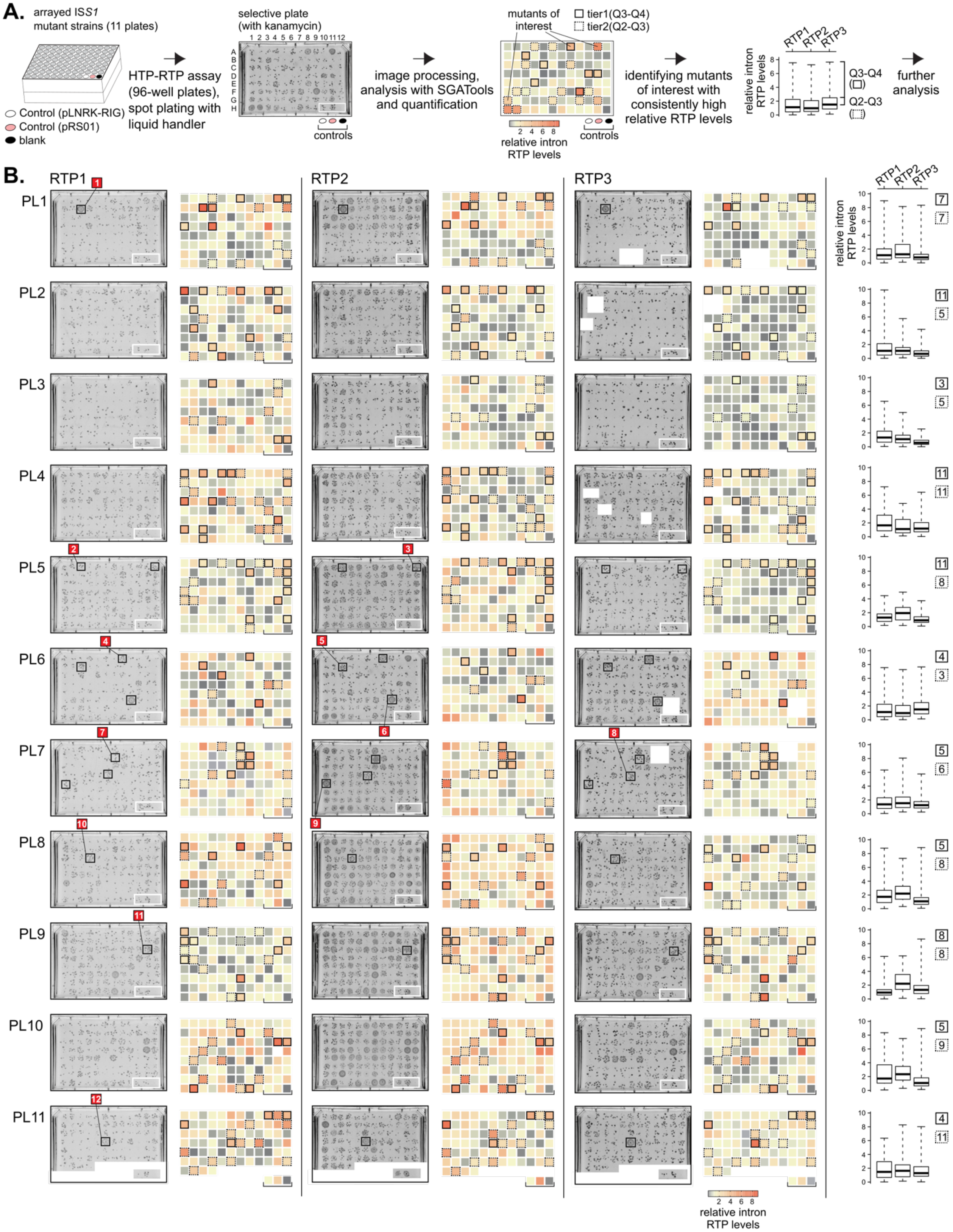


**Figure S5.** High-throughput RTP assay

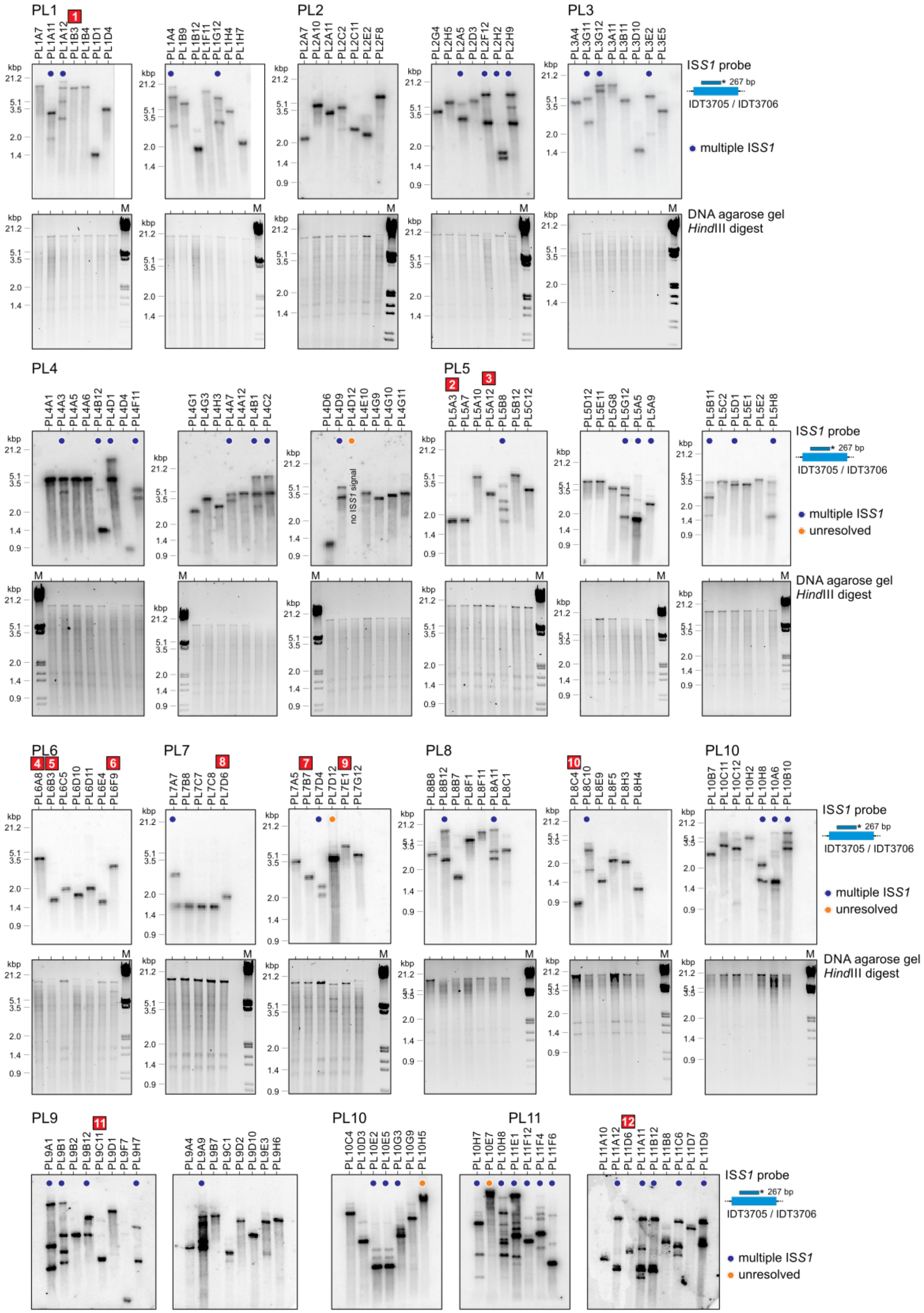


**Figure S6.** Southern blots of RTP-up mutants

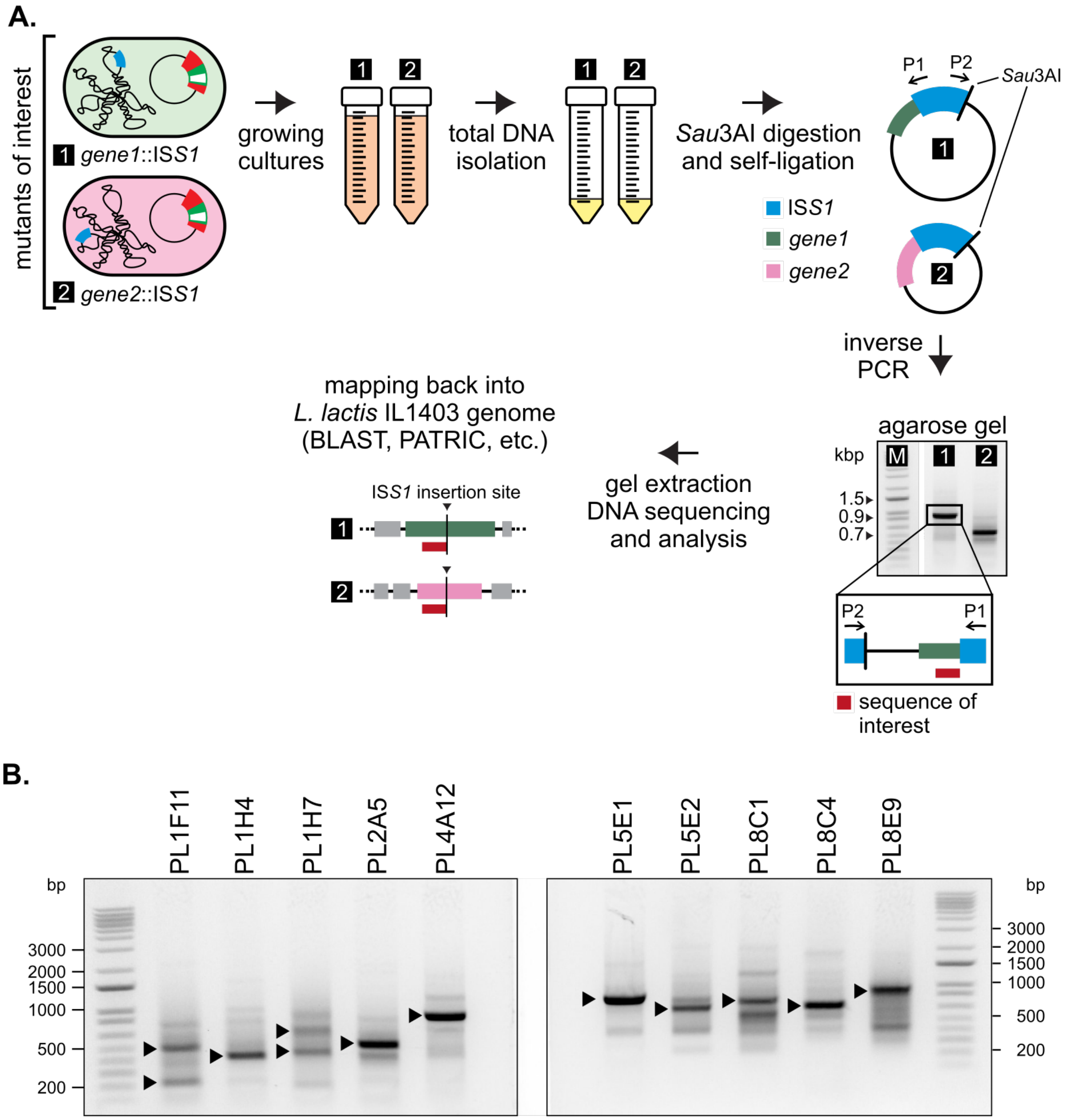


**Figure S7.** Inverse PCR of mutants

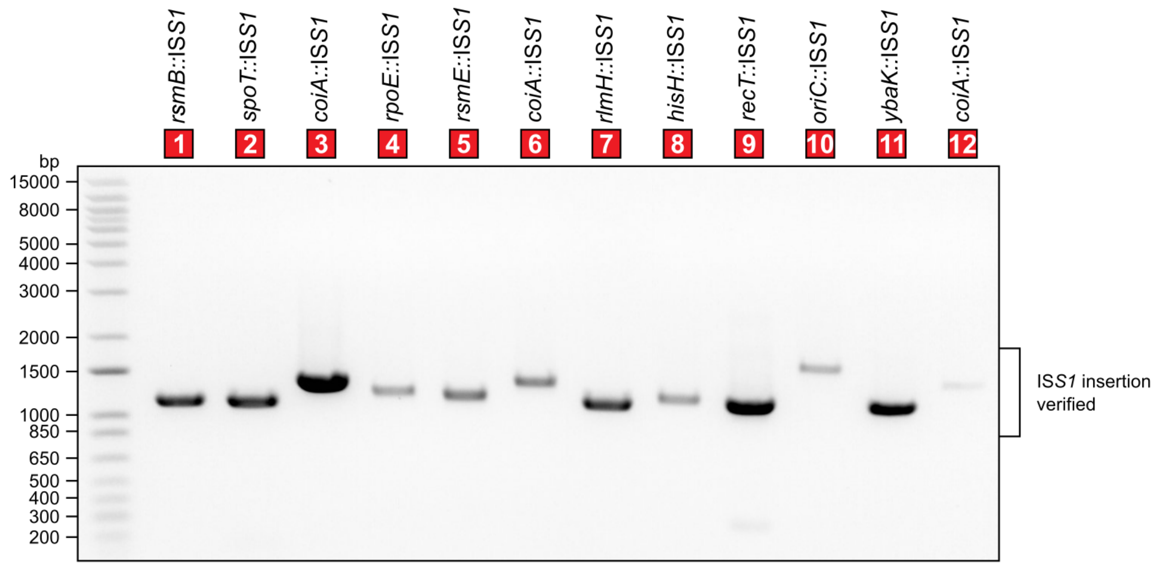


**Figure S8.** Verification PCR gel

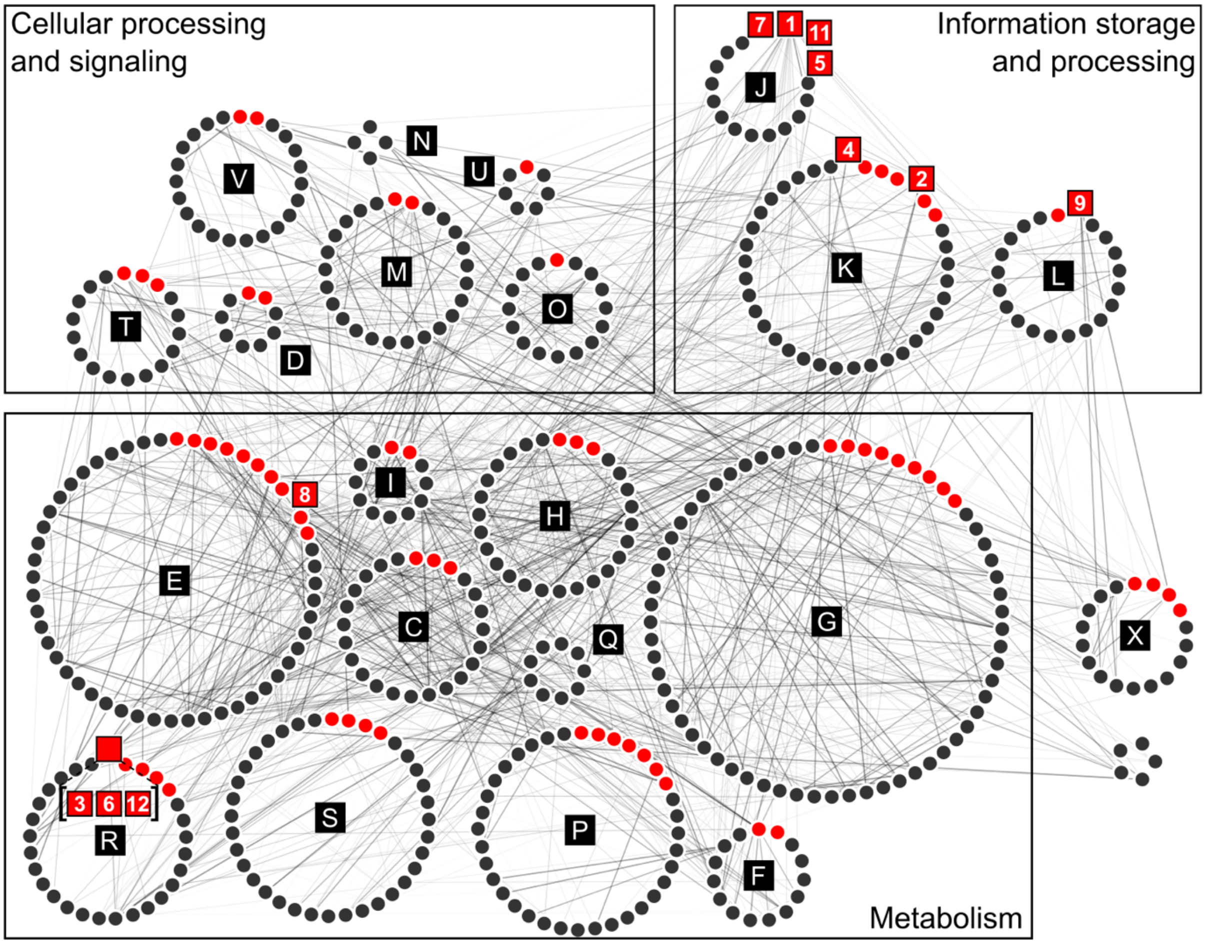


**Figure S9.** STRING protein interaction network

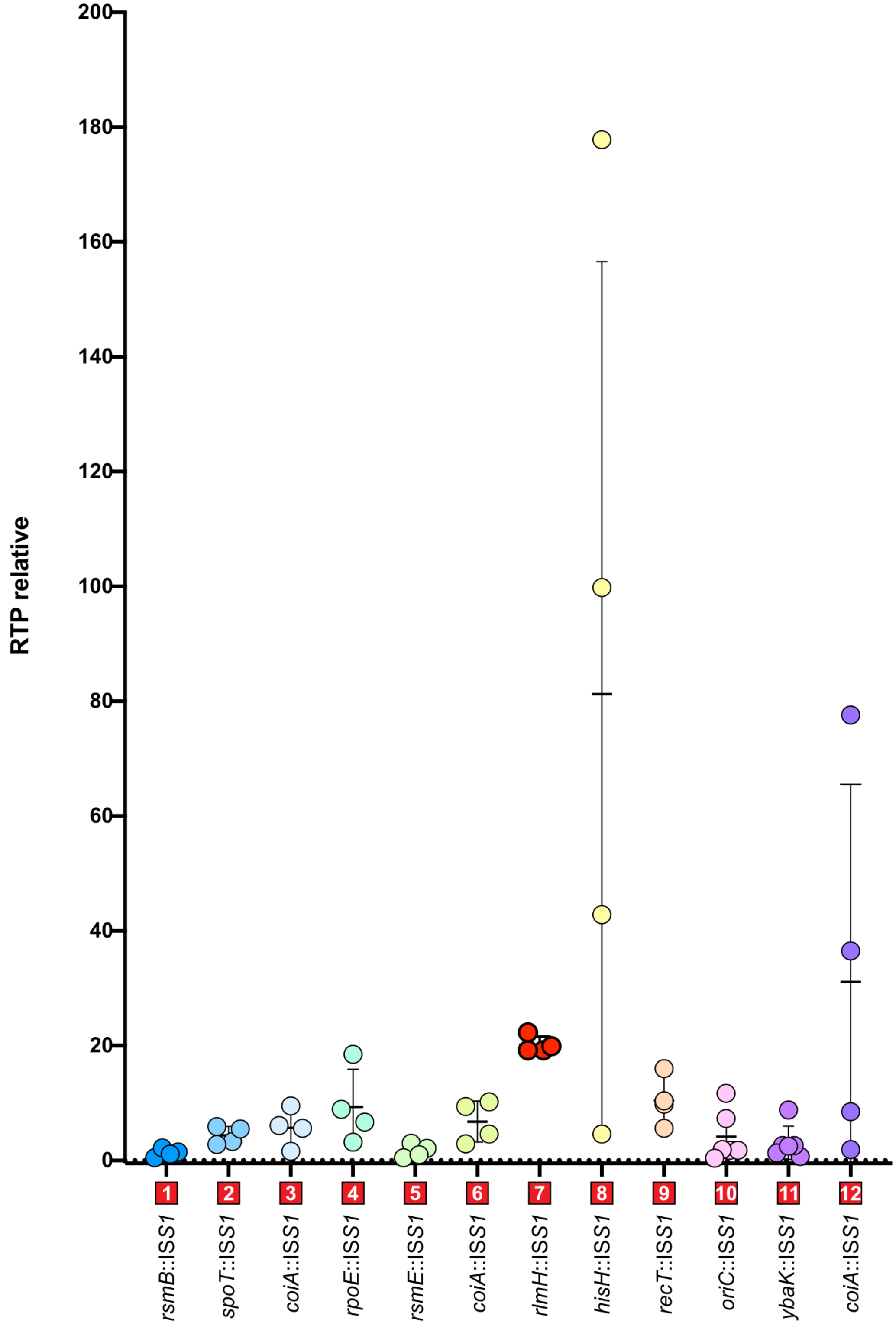


**Figure S10.** Retrotransposition frequency relative to control

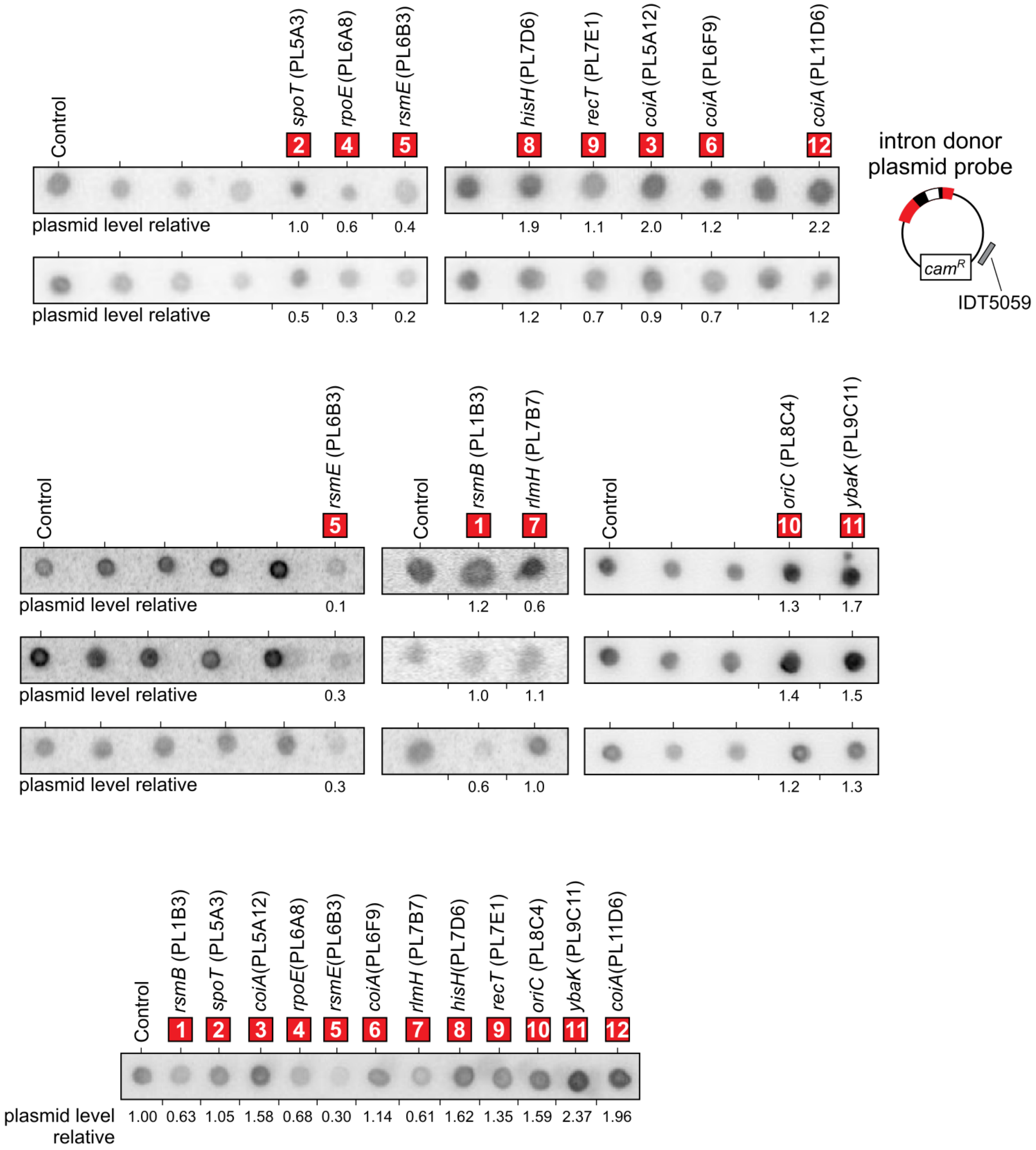


**Figure S11.** Characterization: dot blot hybridization of plasmid DNA

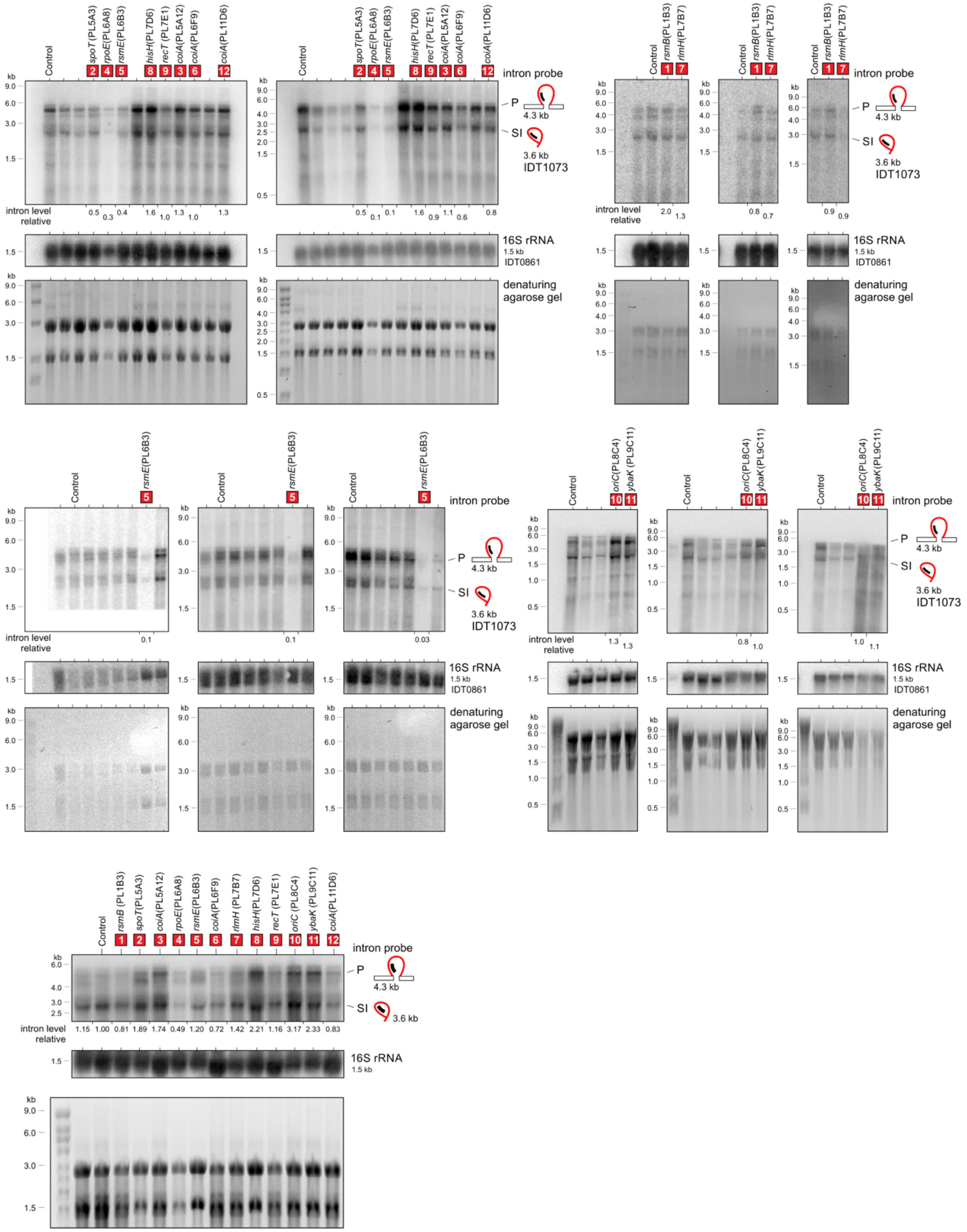

**Figure S12.** Characterization: intron RNA quantitation by Northern blots

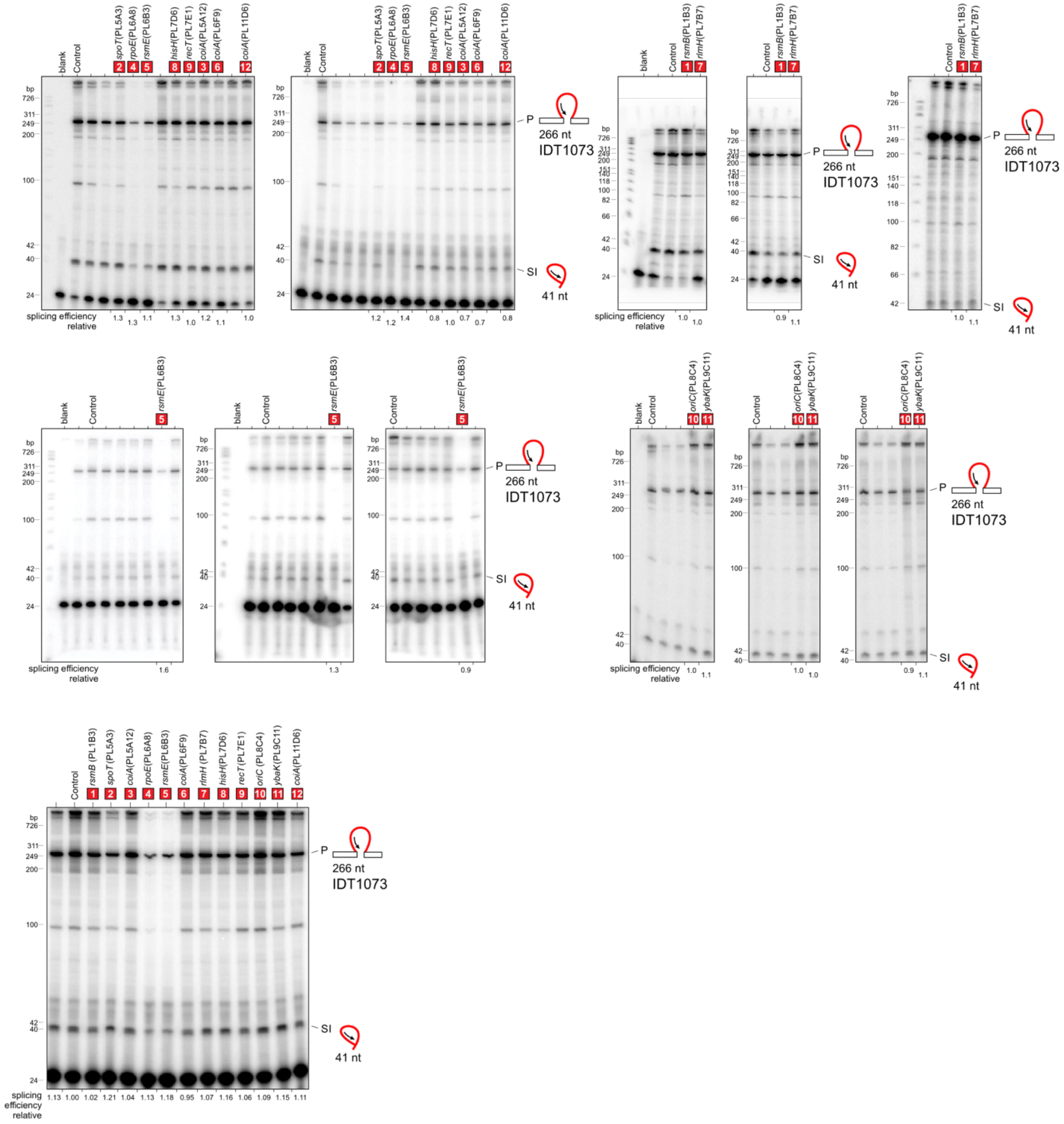


**Figure S13.** Characterization: splicing quantitation by primer extension analysis

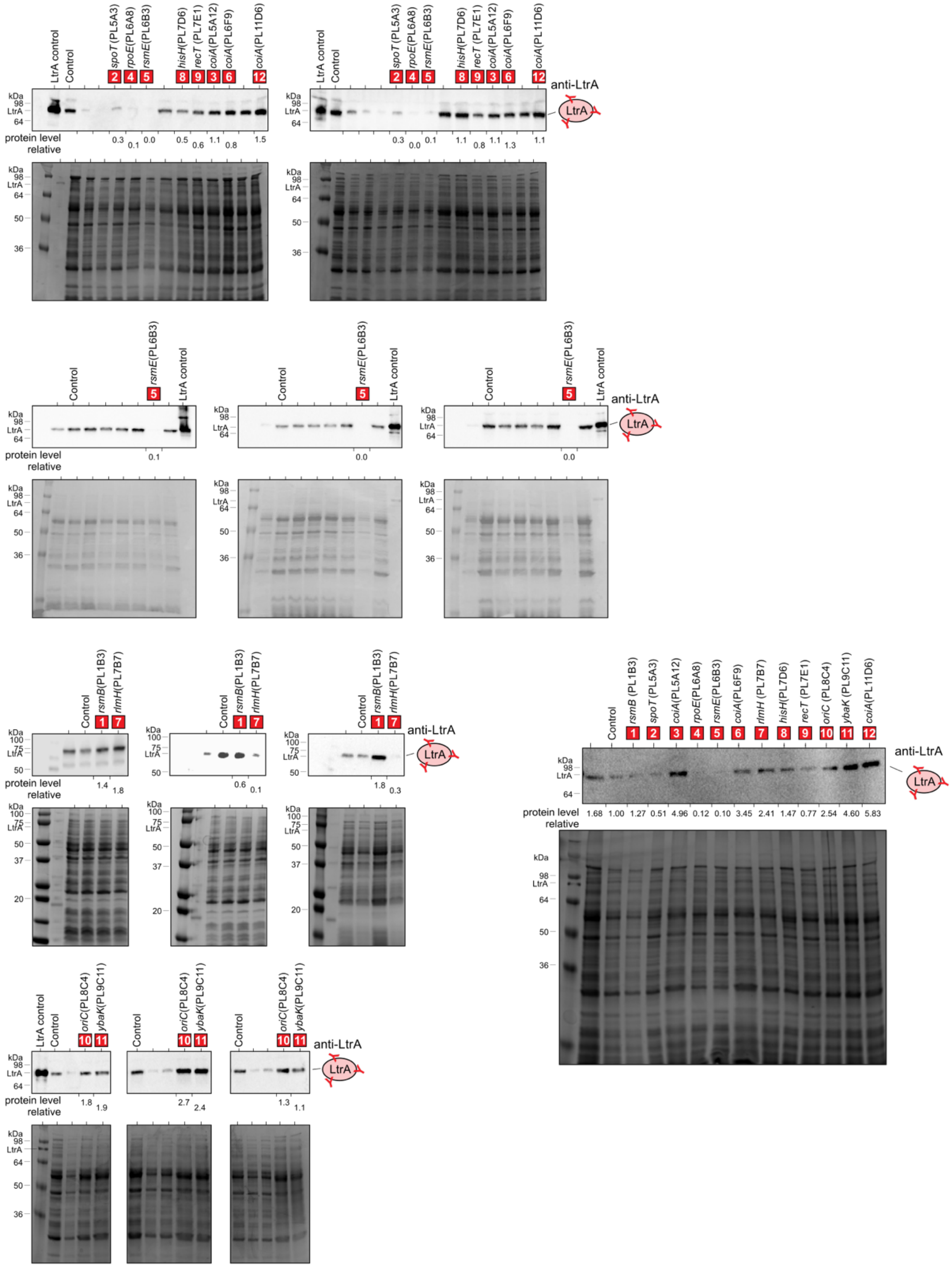


**Figure S14.** Characterization: intron-encoded protein level by Western blots

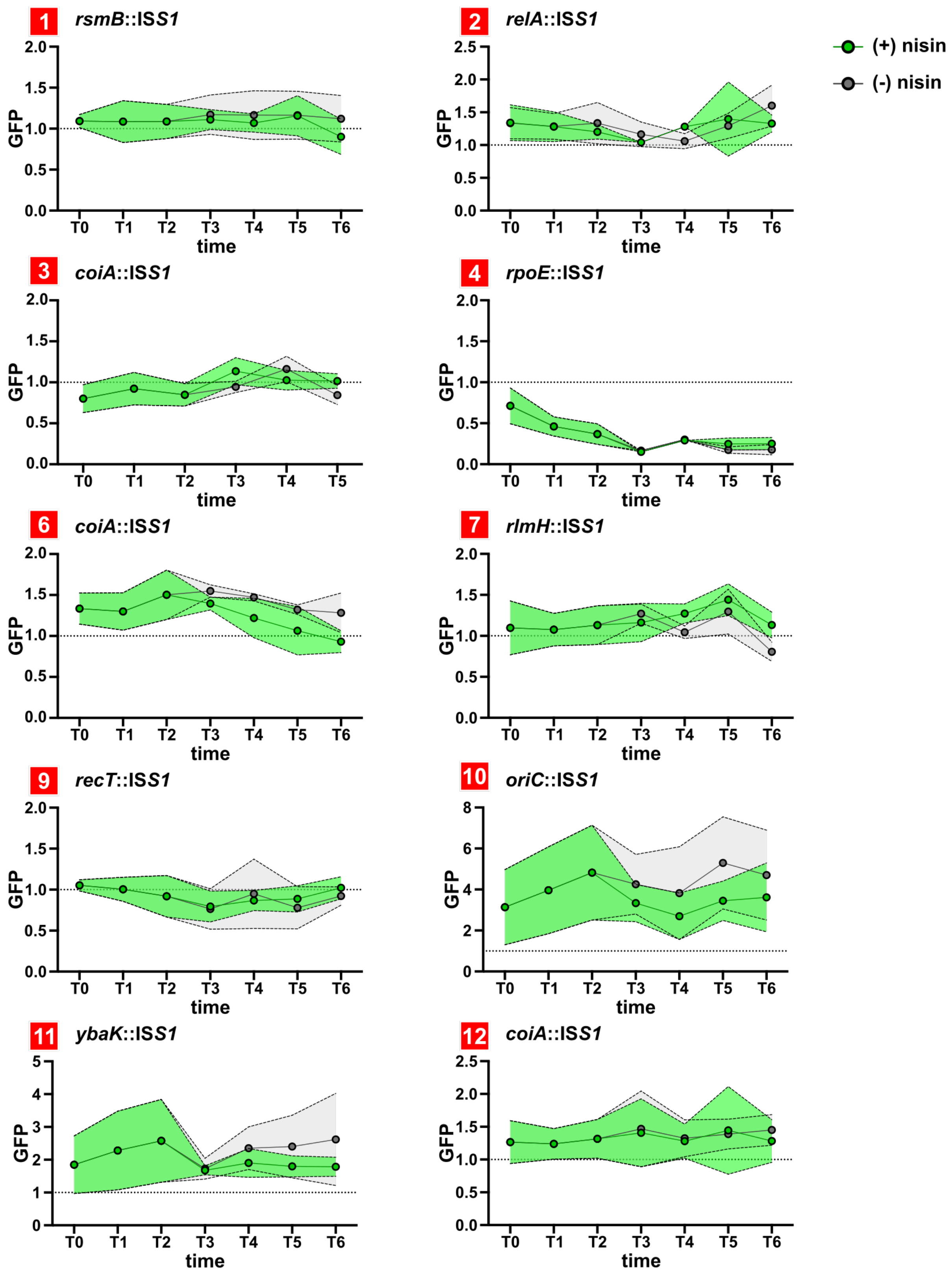


**Figure S15.** Induction from the $P_{nisA}$ promoter

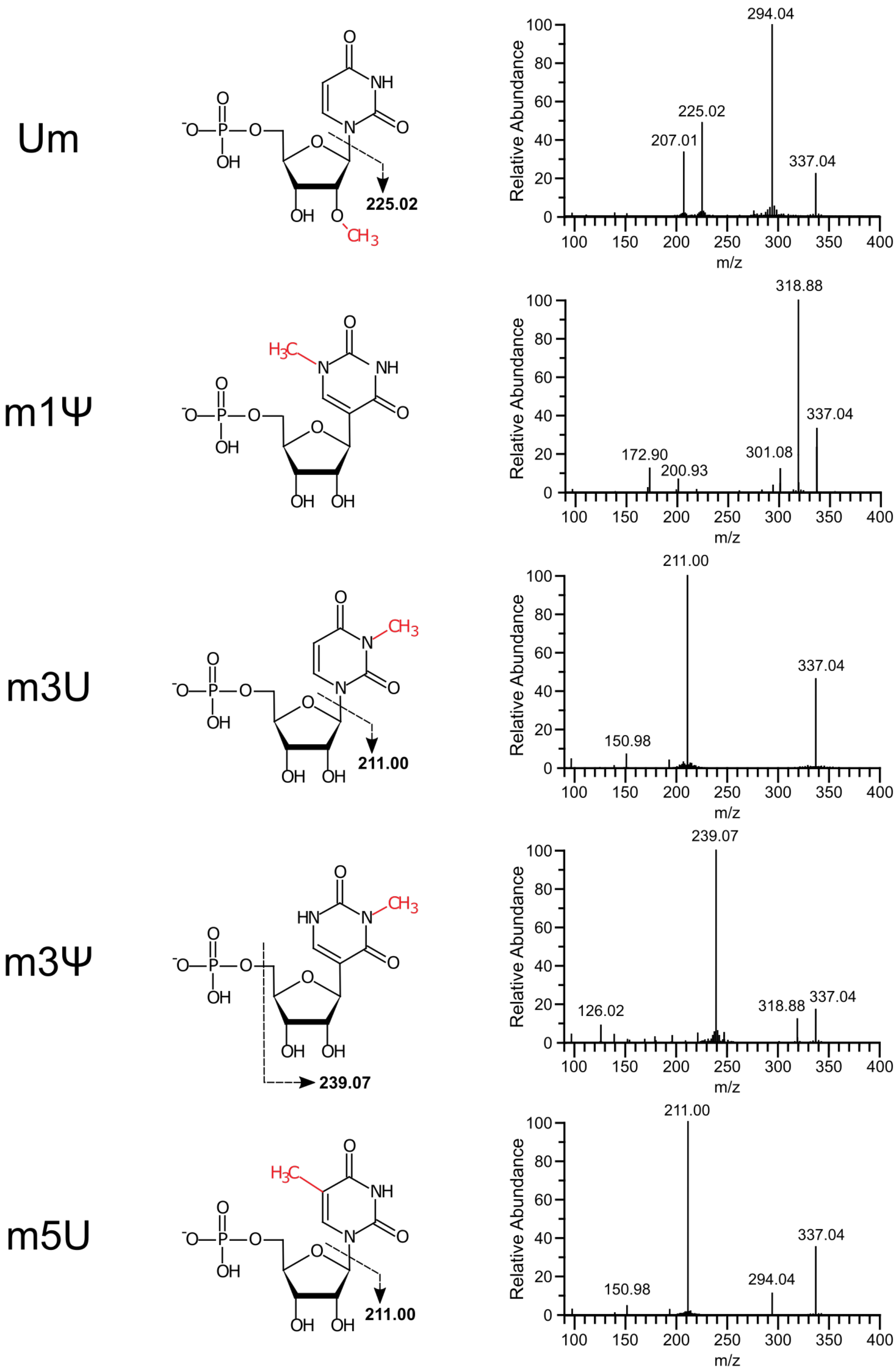


**Figure S16.** Tandem MS/MS analysis of methylated uridine/pseudouridine standards

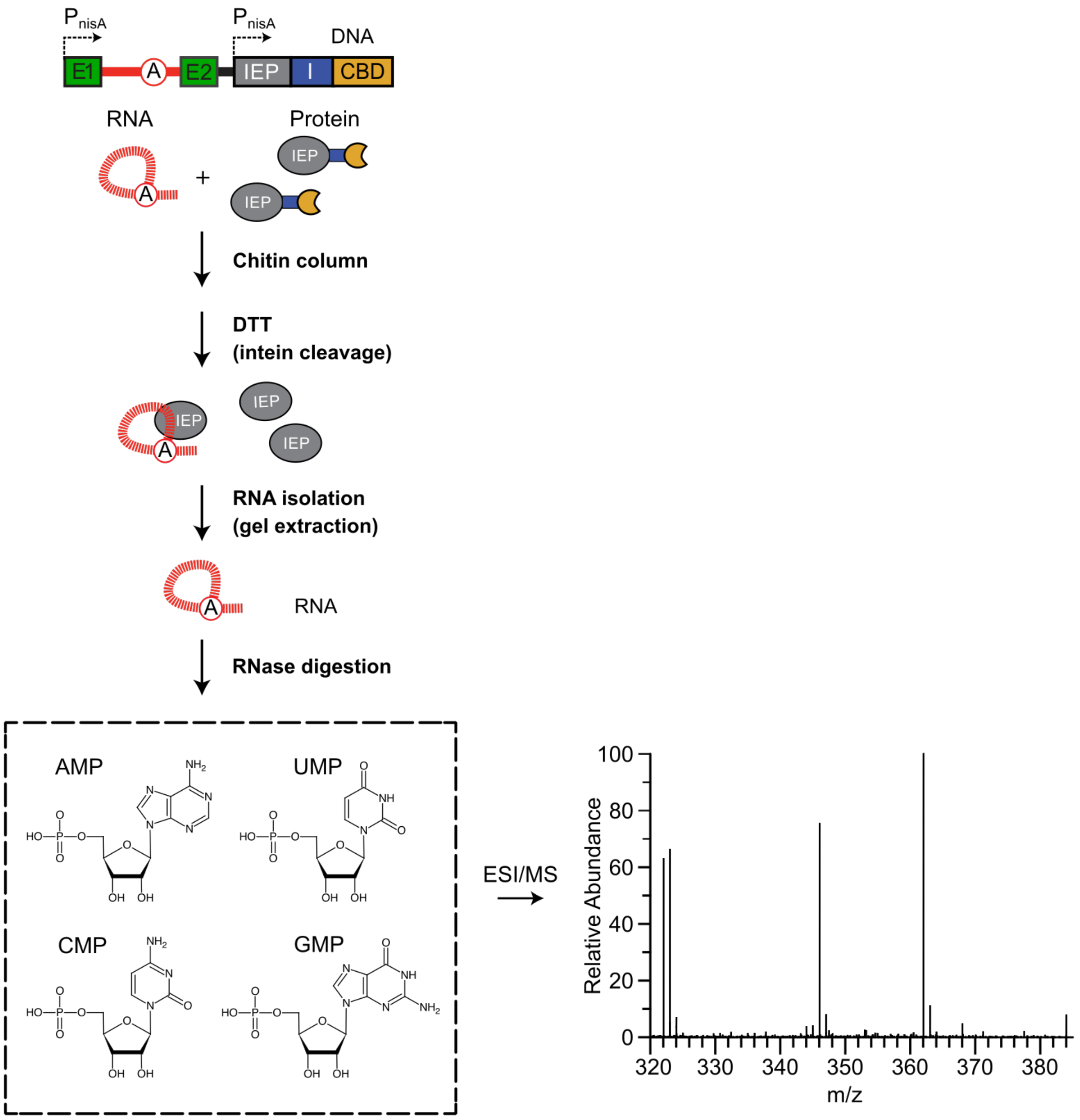


**Figure S17.** Preparation of group II intron RNA samples for MS analysis

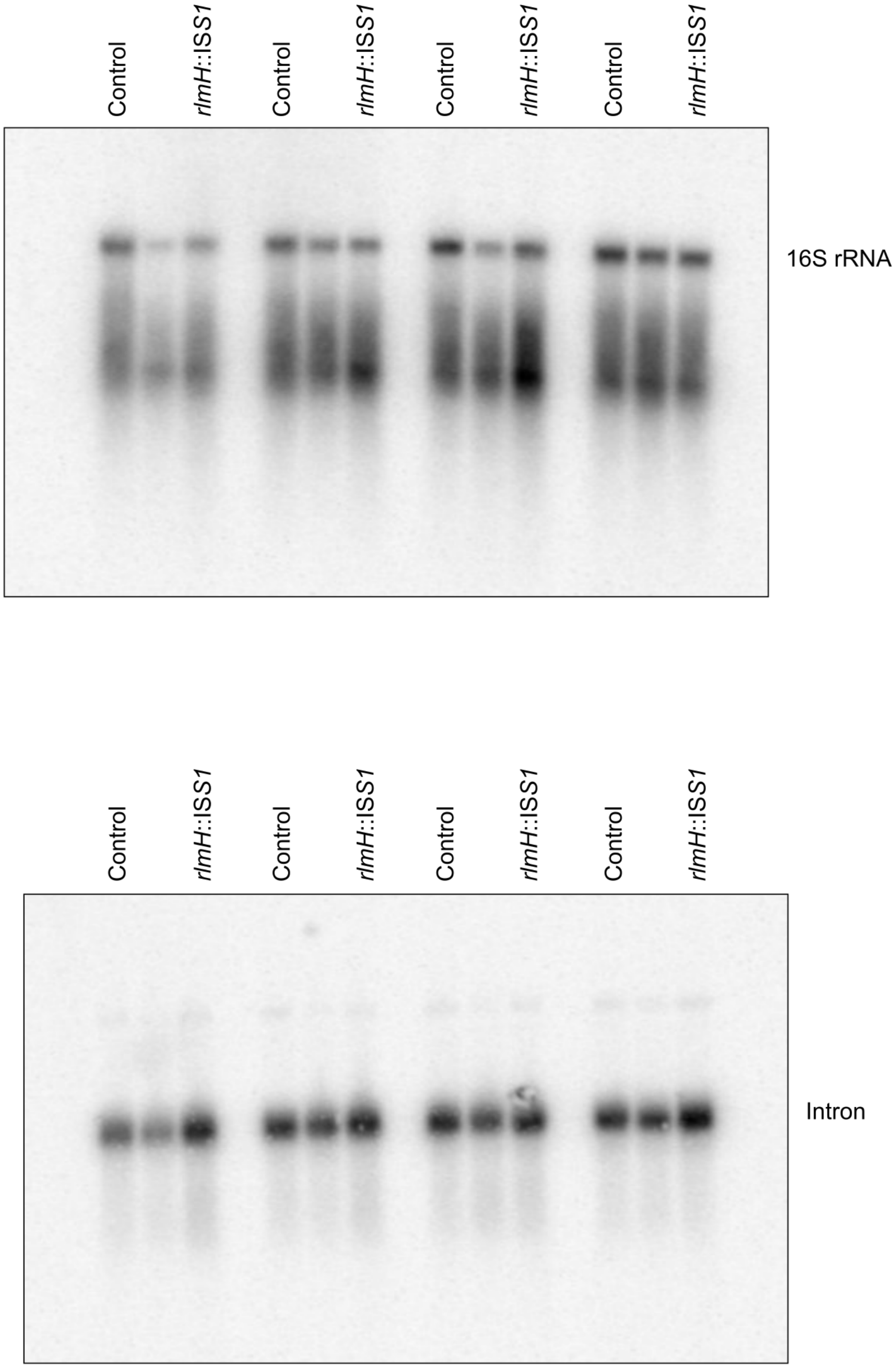


**Figure S18.** Intron RNA pull-down blots

# Supplementary Tables

**Table S1** Southern blot random set summary

| | Mutant Plate ID | Southern Result |
|---|---|---|
| 1 | **PL1A11** | SINGLE |
| 2 | **PL1D2** | MULTIPLE |
| 3 | **PL1D5** | MULTIPLE |
| 4 | **PL1H5** | SINGLE |
| 5 | **PL2A10** | SINGLE |
| 6 | **PL2B9** | SINGLE |
| 7 | **PL2E12** | SINGLE |
| 8 | **PL2G4** | SINGLE |
| 9 | **PL3C8** | SINGLE |
| 10 | **PL3D1** | SINGLE |
| 11 | **PL3E1** | MULTIPLE |
| 12 | **PL3E12** | UNRESOLVED |
| 13 | **PL3F5** | SINGLE |
| 14 | **PL4B3** | SINGLE |
| 15 | **PL4C7** | SINGLE |
| 16 | **PL4E2** | SINGLE |
| 17 | **PL4G7** | SINGLE |
| 18 | **PL5A5** | MULTIPLE |
| 19 | **PL5D3** | UNRESOLVED |
| 20 | **PL5G10** | SINGLE |
| 21 | **PL6D9** | SINGLE |
| 22 | **PL6E3** | SINGLE |
| 23 | **PL6F9** | SINGLE |
| 24 | **PL6H2** | SINGLE |
| 25 | **PL7A1** | MULTIPLE |
| 26 | **PL7G5** | SINGLE |
| 27 | **PL7G11** | MULTIPLE |
| 28 | **PL7H9** | MULTIPLE |
| 29 | **PL8A4** | SINGLE |
| 30 | **PL8C11** | SINGLE |
| 31 | **PL8F8** | SINGLE |
| 32 | **PL8G11** | UNRESOLVED |
| 33 | **PL8G12** | SINGLE |
| 34 | **PL9A4** | SINGLE |
| 35 | **PL9A8** | SINGLE |
| 36 | **PL9B12** | MULTIPLE |
| 37 | **PL9F10** | SINGLE |
| 38 | **PL9F12** | SINGLE |
| 39 | **PL9G12** | MULTIPLE |
| 40 | **PL9H1** | MULTIPLE |
| 41 | **PL10A5** | SINGLE |
| 42 | **PL10A11** | UNRESOLVED |
| 43 | **PL10B1** | SINGLE |
| 44 | **PL10C1** | MULTIPLE |
| 45 | **PL10D9** | SINGLE |
| 46 | **PL10G1** | SINGLE |
| 47 | **PL11A1** | SINGLE |
| 48 | **PL11C4** | NO_SIGNAL |
| 49 | **PL11D3** | SINGLE |
| 50 | **PL11D8** | SINGLE |

**Table S2** COG counts

| COG CATEGORY | GENOME COUNT | GENOME FRACTION (%) | LIBRARY COUNT | LIBRARY FRACTION (%) | RTP-UP MUTANTS COUNT | RTP-UP MUTANTS FRACTION (%) |
|---|---|---|---|---|---|---|
| **J*** | 210 | **8.41** | 19 | **3.10** | 4 | **4.40** |
| **K** | 202 | **8.09** | 43 | **7.01** | 9 | **9.89** |
| **L** | 128 | **5.13** | 24 | **3.92** | 3 | **3.30** |
| **D** | 39 | **1.56** | 10 | **1.63** | 2 | **2.20** |
| **M** | 148 | **5.93** | 35 | **5.71** | 4 | **4.40** |
| **N** | 36 | **1.44** | 7 | **1.14** | 0 | **0.00** |
| **O** | 86 | **3.45** | 16 | **2.61** | 1 | **1.10** |
| **T** | 108 | **4.33** | 26 | **4.24** | 5 | **5.49** |
| **C** | 91 | **3.65** | 26 | **4.24** | 4 | **4.40** |
| **E*** | 209 | **8.37** | 63 | **10.28** | 15 | **16.48** |
| **F*** | 99 | **3.97** | 15 | **2.45** | 2 | **2.20** |
| **G*** | 187 | **7.49** | 68 | **11.09** | 9 | **9.89** |
| **H** | 112 | **4.49** | 31 | **5.06** | 4 | **4.40** |
| **I** | 85 | **3.41** | 20 | **3.26** | 2 | **2.20** |
| **P** | 120 | **4.81** | 36 | **5.87** | 7 | **7.69** |
| **Q** | 40 | **1.60** | 10 | **1.63** | 0 | **0.00** |
| **R** | 234 | **9.38** | 62 | **10.11** | 7 | **7.69** |
| **S** | 177 | **7.09** | 42 | **6.85** | 4 | **4.40** |
| **U** | 25 | **1.00** | 8 | **1.31** | 1 | **1.10** |
| **V*** | 86 | **3.45** | 29 | **4.73** | 4 | **4.40** |
| **W** | 6 | **0.24** | 3 | **0.49** | 0 | **0.00** |
| **X** | 68 | **2.72** | 20 | **3.26** | 4 | **4.40** |
| **Total** | **2496** | **100** | **613** | **100** | **91** | **100** |

COG category counts for genome, library, and RTP-up mutants. Relative abundance, or fraction, is also reported as a percentage. Asterisks represent categories where there is a significant difference. Statistical comparisons were made using a hypergeometric test between the genome and the library, as well as between the library and the RTP-up mutants. Statistical significance ($p < 0.05$) between data sets is shown with a bolded border around the cells reporting the fraction a category represents. Only category E shows a statistically significant difference between both the genome and the library, and between the library and the RTP-up mutants. No comparisons were made between the genome and RTP-up mutants.

**Table S3** COG category key

| COG Category | Function |
|---|---|
| **Information storage and processing** | |
| J | Translation, ribosomal structure and biogenesis |
| K | Transcription |
| L | Replication, recombination and repair |
| **Cellular processing and signaling** | |
| D | Cell cycle control, cell division, chromosome partitioning |
| M | Cell wall/membrane/envelope biogenesis |
| N | Cell motility |
| O | Post-translational modification, protein turnover, and chaperones |
| T | Signal transduction mechanisms |
| **Metabolism** | |
| C | Energy production and conversion |
| E | Amino acid transport and metabolism |
| F | Nucleotide transport and metabolism |
| G | Carbohydrate transport and metabolism |
| H | Coenzyme transport and metabolism |
| I | Lipid transport and metabolism |
| P | Inorganic ion transport and metabolism |
| Q | Secondary metabolites biosynthesis, transport, and catabolism |
| R | General function prediction only |
| S | Function unknown |
| U | Intracellular trafficking, secretion, and vesicular transport |
| V | Defense mechanisms |
| W | Extracellular structures |
| **Other** | |
| X | Mobile elements and transposases |

**Table S4** RTP-up preliminary list

| TOP-12 NUMBER | MUTANT TIER | MUTANT PLATE ID | SOUTHERN | PURGED | HTP-RTP1 | HTP-RTP2 | HTP-RTP3 |
|---|---|---|---|---|---|---|---|
| | Q3_Q4 | PL1A7 | SINGLE | N | 3.09 | 2.92 | 3.57 |
| | Q3_Q4 | PL1A11 | MULTIPLE | Y | 6.18 | 4.68 | 3.27 |
| | Q3_Q4 | PL1A12 | MULTIPLE | Y | 2.30 | 3.09 | 2.55 |
| 1 | Q3_Q4 | PL1B3 | SINGLE | N | 8.99 | 8.16 | 8.35 |
| | Q3_Q4 | PL1B4 | SINGLE | N | 5.91 | 2.95 | 1.48 |
| | Q3_Q4 | PL1D1 | SINGLE | N | 2.47 | 5.15 | 2.39 |
| | Q3_Q4 | PL1D4 | SINGLE | N | 8.23 | 5.60 | 3.12 |
| | Q2_Q3 | PL1A4 | MULTIPLE | Y | 1.71 | 5.76 | 1.42 |
| | Q2_Q3 | PL1B9 | SINGLE | N | 4.33 | 4.56 | 1.14 |
| | Q2_Q3 | PL1B12 | SINGLE | N | 6.36 | 4.39 | 1.16 |
| | Q2_Q3 | PL1F11 | SINGLE | N | 1.40 | 3.02 | 1.71 |
| | Q2_Q3 | PL1G12 | MULTIPLE | Y | 2.08 | 1.79 | 1.42 |
| | Q2_Q3 | PL1H4 | SINGLE | N | 1.18 | 4.91 | 2.02 |
| | Q2_Q3 | PL1H7 | SINGLE | N | 2.03 | 2.79 | |
| | Q3_Q4 | PL2A1 | SINGLE | N | 8.89 | 5.75 | 2.85 |
| | Q3_Q4 | PL2A3 | SINGLE | N | 3.10 | 3.74 | 2.12 |
| | Q3_Q4 | PL2A7 | SINGLE | N | 5.36 | 3.33 | 2.08 |
| | Q3_Q4 | PL2A10 | SINGLE | N | 5.72 | 5.21 | 1.95 |
| | Q3_Q4 | PL2A11 | SINGLE | N | 2.13 | 1.68 | 1.21 |
| | Q3_Q4 | PL2C2 | SINGLE | N | 2.64 | 1.60 | |
| | Q3_Q4 | PL2C11 | SINGLE | N | 5.15 | 2.25 | 2.10 |
| | Q3_Q4 | PL2E2 | SINGLE | N | 4.40 | 2.83 | 1.85 |
| | Q3_Q4 | PL2F8 | SINGLE | N | 3.66 | 2.40 | 1.25 |
| | Q3_Q4 | PL2G4 | SINGLE | N | 3.10 | 3.75 | 1.45 |
| | Q3_Q4 | PL2H5 | SINGLE | N | 5.67 | 2.89 | 4.19 |
| | Q2_Q3 | PL2A5 | MULTIPLE | Y | 1.62 | 3.58 | 1.40 |
| | Q2_Q3 | PL2D3 | SINGLE | N | 2.61 | 3.36 | 0.97 |
| | Q2_Q3 | PL2F12 | MULTIPLE | Y | 1.79 | 1.84 | 1.20 |
| | Q2_Q3 | PL2H2 | MULTIPLE | Y | 3.83 | 2.29 | 0.67 |
| | Q2_Q3 | PL2H9 | MULTIPLE | Y | 2.53 | 1.50 | 1.38 |
| | Q3_Q4 | PL3A4 | SINGLE | N | 4.06 | 1.86 | 1.00 |
| | Q3_Q4 | PL3G11 | MULTIPLE | Y | 3.45 | 2.37 | 1.96 |
| | Q3_Q4 | PL3G12 | MULTIPLE | Y | 3.95 | 2.39 | 1.65 |
| | Q2_Q3 | PL3A11 | SINGLE | N | 1.53 | 1.93 | 1.22 |
| | Q2_Q3 | PL3B11 | SINGLE | N | 2.96 | 1.77 | 0.60 |
| | Q2_Q3 | PL3D10 | SINGLE | N | 3.76 | 1.53 | 0.94 |
| | Q2_Q3 | PL3E2 | MULTIPLE | Y | 2.63 | 1.55 | 1.03 |
| | Q2_Q3 | PL3E5 | SINGLE | N | 4.10 | 1.73 | 0.94 |
| | Q3_Q4 | PL4A1 | SINGLE | N | 3.72 | 4.53 | 3.60 |
| | Q3_Q4 | PL4A3 | MULTIPLE | Y | 4.79 | 3.27 | 2.25 |
| | Q3_Q4 | PL4A5 | SINGLE | Y | 5.09 | 2.74 | 2.27 |
| | Q3_Q4 | PL4A6 | SINGLE | Y | 5.29 | 3.38 | 3.27 |
| | Q3_Q4 | PL4B12 | MULTIPLE | Y | 4.39 | 2.78 | 2.41 |
| | Q3_Q4 | PL4D1 | MULTIPLE | Y | 7.20 | 4.55 | 6.42 |
| | Q3_Q4 | PL4D4 | SINGLE | N | 6.10 | 3.79 | 3.04 |
| | Q3_Q4 | PL4F11 | MULTIPLE | Y | 6.34 | 2.75 | 3.65 |

**Table S4** RTP-up preliminary list (continued)

| TOP-12 NUMBER | MUTANT TIER | MUTANT PLATE ID | SOUTHERN | PURGED | HTP-RTP1 | HTP-RTP2 | HTP-RTP3 |
|---|---|---|---|---|---|---|---|
| | Q3_Q4 | PL4G1 | SINGLE | N | 4.74 | 4.19 | 2.50 |
| | Q3_Q4 | PL4G3 | SINGLE | N | 3.52 | 3.09 | 3.13 |
| | Q3_Q4 | PL4H3 | SINGLE | Y | 3.17 | 3.44 | 2.29 |
| | Q2_Q3 | PL4A7 | MULTIPLE | Y | 3.32 | 2.12 | 3.19 |
| | Q2_Q3 | PL4A12 | SINGLE | N | 2.88 | 3.94 | 2.57 |
| | Q2_Q3 | PL4B1 | MULTIPLE | Y | 1.89 | 4.80 | 3.65 |
| | Q2_Q3 | PL4C2 | MULTIPLE | Y | 3.37 | 2.18 | |
| | Q2_Q3 | PL4D6 | SINGLE | N | 4.34 | 1.66 | 2.44 |
| | Q2_Q3 | PL4D9 | MULTIPLE | Y | 3.38 | 2.60 | 2.07 |
| | Q2_Q3 | PL4D12 | NO_SIGNAL | Y | 3.76 | 2.58 | 1.65 |
| | Q2_Q3 | PL4E10 | SINGLE | Y | 4.43 | 2.77 | 1.50 |
| | Q2_Q3 | PL4G9 | SINGLE | N | 4.86 | 1.50 | 2.79 |
| | Q2_Q3 | PL4G10 | SINGLE | N | 3.81 | 2.18 | 2.76 |
| | Q2_Q3 | PL4G11 | SINGLE | N | 3.19 | 2.55 | 1.83 |
| 2 | Q3_Q4 | PL5A3 | SINGLE | N | 1.95 | 3.17 | 1.66 |
| | Q3_Q4 | PL5A7 | SINGLE | N | 2.41 | 4.61 | 2.26 |
| | Q3_Q4 | PL5A10 | SINGLE | N | 2.33 | 3.71 | 2.40 |
| 3 | Q3_Q4 | PL5A12 | SINGLE | N | 3.41 | 3.82 | 1.41 |
| | Q3_Q4 | PL5B8 | MULTIPLE | Y | 3.80 | 4.94 | 1.98 |
| | Q3_Q4 | PL5B12 | SINGLE | N | 3.34 | 2.99 | 3.70 |
| | Q3_Q4 | PL5C12 | SINGLE | N | 2.13 | 4.74 | 2.14 |
| | Q3_Q4 | PL5D12 | SINGLE | N | 2.23 | 2.85 | 2.45 |
| | Q3_Q4 | PL5E11 | SINGLE | N | 4.43 | 3.10 | 1.51 |
| | Q3_Q4 | PL5G8 | SINGLE | N | 2.70 | 3.21 | 1.39 |
| | Q3_Q4 | PL5G12 | MULTIPLE | Y | 1.96 | 4.41 | 2.21 |
| | Q2_Q3 | PL5A5 | MULTIPLE | Y | 1.60 | 3.83 | 2.12 |
| | Q2_Q3 | PL5A9 | MULTIPLE | Y | 1.36 | 3.46 | 1.72 |
| | Q2_Q3 | PL5B11 | MULTIPLE | Y | 3.52 | 2.38 | 1.88 |
| | Q2_Q3 | PL5C2 | SINGLE | Y | 1.57 | 4.59 | 1.51 |
| | Q2_Q3 | PL5D1 | MULTIPLE | Y | 2.54 | 2.15 | 2.27 |
| | Q2_Q3 | PL5E1 | SINGLE | N | 2.66 | 3.35 | 1.36 |
| | Q2_Q3 | PL5E2 | SINGLE | N | 2.51 | 2.25 | 2.01 |
| | Q2_Q3 | PL5H8 | MULTIPLE | Y | 1.97 | 3.36 | 0.89 |
| 4 | Q3_Q4 | PL6A8 | SINGLE | N | 4.72 | 4.36 | 7.64 |
| 5 | Q3_Q4 | PL6B3 | SINGLE | N | 5.25 | 3.39 | 3.90 |
| | Q3_Q4 | PL6C5 | SINGLE | N | 5.77 | 2.30 | 2.51 |
| 6 | Q3_Q4 | PL6F9 | SINGLE | N | 7.49 | 7.25 | 7.59 |
| | Q2_Q3 | PL6D10 | SINGLE | N | 7.54 | 3.82 | 3.66 |
| | Q2_Q3 | PL6D11 | SINGLE | N | 2.65 | 3.12 | 4.41 |
| | Q2_Q3 | PL6E4 | SINGLE | N | 2.41 | 2.56 | 2.55 |
| | Q3_Q4 | PL7A7 | MULTIPLE | Y | 2.71 | 5.33 | 5.72 |
| | Q3_Q4 | PL7B8 | SINGLE | N | 5.47 | 3.95 | 3.51 |
| | Q3_Q4 | PL7C7 | SINGLE | Y | 4.14 | 3.15 | 3.29 |
| | Q3_Q4 | PL7C8 | SINGLE | Y | 4.40 | 4.34 | 3.80 |

**Table S4** RTP-up preliminary list (continued)

| TOP-12 NUMBER | MUTANT TIER | MUTANT PLATE ID | SOUTHERN | PURGED | HTP-RTP1 | HTP-RTP2 | HTP-RTP3 |
|---|---|---|---|---|---|---|---|
| 8 | Q3_Q4 | PL7D6 | SINGLE | N | 3.88 | 2.41 | 3.06 |
| | Q2_Q3 | PL7A5 | SINGLE | N | 3.70 | 2.00 | 2.40 |
| 7 | Q2_Q3 | PL7B7 | SINGLE | N | 2.10 | 6.47 | 2.13 |
| | Q2_Q3 | PL7D4 | MULTIPLE | Y | 3.67 | 2.23 | 3.11 |
| | Q2_Q3 | PL7D12 | MULTIPLE | Y | 3.45 | 2.38 | 2.24 |
| 9 | Q2_Q3 | PL7E1 | SINGLE | N | 1.62 | 8.02 | 3.63 |
| | Q2_Q3 | PL7G12 | SINGLE | N | 2.27 | 2.49 | 1.32 |
| | Q3_Q4 | PL8B1 | SINGLE | N | 5.71 | 5.12 | 2.05 |
| | Q3_Q4 | PL8B7 | SINGLE | N | 8.77 | 5.93 | 3.44 |
| | Q3_Q4 | PL8B12 | MULTIPLE | Y | 3.72 | 3.53 | 1.90 |
| | Q3_Q4 | PL8F1 | SINGLE | N | 8.14 | 7.29 | 8.85 |
| | Q3_Q4 | PL8F11 | SINGLE | N | 3.96 | 5.48 | 3.15 |
| | Q2_Q3 | PL8A11 | MULTIPLE | Y | 5.41 | 3.50 | 1.48 |
| 10 | Q2_Q3 | PL8C1 | SINGLE | N | 4.81 | 2.22 | 1.74 |
| | Q2_Q3 | PL8C4 | SINGLE | N | 5.52 | 3.25 | 2.63 |
| | Q2_Q3 | PL8C10 | MULTIPLE | Y | 3.63 | 4.26 | 1.29 |
| | Q2_Q3 | PL8E9 | SINGLE | N | 2.48 | 5.66 | 2.46 |
| | Q2_Q3 | PL8F5 | SINGLE | N | 4.69 | 3.39 | 1.58 |
| | Q2_Q3 | PL8H3 | SINGLE | N | 3.24 | 3.72 | 1.59 |
| | Q2_Q3 | PL8H4 | SINGLE | Y | 4.21 | 5.58 | 1.44 |
| | Q3_Q4 | PL9A1 | MULTIPLE | Y | 3.25 | 4.70 | 4.73 |
| | Q3_Q4 | PL9B1 | MULTIPLE | Y | 2.56 | 4.48 | 3.27 |
| | Q3_Q4 | PL9B2 | SINGLE | N | 1.52 | 3.81 | 2.39 |
| | Q3_Q4 | PL9B12 | MULTIPLE | Y | 3.35 | 5.92 | 2.66 |
| 11 | Q3_Q4 | PL9C11 | SINGLE | N | 3.45 | 4.15 | 2.36 |
| | Q3_Q4 | PL9D1 | SINGLE | N | 1.74 | 3.58 | 4.40 |
| | Q3_Q4 | PL9F7 | SINGLE | N | 6.16 | 5.30 | 8.67 |
| | Q3_Q4 | PL9H7 | MULTIPLE | Y | 2.84 | 5.40 | 8.28 |
| | Q2_Q3 | PL9A4 | SINGLE | N | 1.70 | 2.42 | 2.04 |
| | Q2_Q3 | PL9A9 | MULTIPLE | Y | 1.30 | 6.23 | 2.14 |
| | Q2_Q3 | PL9B7 | SINGLE | N | 0.61 | 4.39 | 1.54 |
| | Q2_Q3 | PL9C1 | SINGLE | Y | 1.18 | 3.86 | 1.98 |
| | Q2_Q3 | PL9D2 | SINGLE | N | 1.79 | 2.86 | 2.46 |
| | Q2_Q3 | PL9D10 | SINGLE | N | 2.29 | 3.33 | 5.95 |
| | Q2_Q3 | PL9E3 | SINGLE | N | 1.81 | 2.23 | 1.36 |
| | Q2_Q3 | PL9H6 | SINGLE | N | 1.26 | 5.16 | 2.10 |
| | Q3_Q4 | PL10B7 | SINGLE | N | 5.55 | 5.91 | 1.97 |
| | Q3_Q4 | PL10C11 | SINGLE | N | 8.29 | 7.13 | 4.07 |
| | Q3_Q4 | PL10C12 | SINGLE | N | 4.98 | 3.45 | 3.85 |
| | Q3_Q4 | PL10H2 | SINGLE | N | 7.58 | 6.94 | 2.85 |
| | Q3_Q4 | PL10H8 | MULTIPLE | Y | 4.88 | 5.49 | 5.62 |
| | Q2_Q3 | PL10A6 | MULTIPLE | Y | 3.34 | 5.58 | 4.56 |
| | Q2_Q3 | PL10B10 | MULTIPLE | Y | 5.27 | 3.34 | 4.50 |
| | Q2_Q3 | PL10C4 | SINGLE | N | 3.86 | 4.36 | 1.62 |
| | Q2_Q3 | PL10D3 | SINGLE | N | 4.41 | 2.61 | 2.10 |
| | Q2_Q3 | PL10E2 | MULTIPLE | Y | 3.04 | 6.48 | 1.77 |

**Table S4** RTP-up preliminary list (continued)

| TOP-12 NUMBER | MUTANT TIER | MUTANT PLATE ID | SOUTHERN | PURGED | HTP-RTP1 | HTP-RTP2 | HTP-RTP3 |
|---|---|---|---|---|---|---|---|
| | Q2_Q3 | PL10E5 | MULTIPLE | Y | 5.56 | 3.28 | 5.14 |
| | Q2_Q3 | PL10G3 | MULTIPLE | Y | 6.02 | 7.40 | 1.71 |
| | Q2_Q3 | PL10G9 | SINGLE | N | 3.77 | 2.51 | 1.90 |
| | Q2_Q3 | PL10H7 | MULTIPLE | Y | 2.60 | 7.27 | 2.41 |
| | Q3_Q4 | PL11A10 | SINGLE | N | 4.73 | 4.96 | 2.21 |
| | Q3_Q4 | PL11A12 | MULTIPLE | Y | 4.34 | 4.29 | 2.79 |
| | Q3_Q4 | PL11B1 | SINGLE | N | 4.46 | 8.26 | 5.16 |
| 12 | Q3_Q4 | PL11D6 | SINGLE | N | 4.01 | 4.39 | 8.00 |
| | Q2_Q3 | PL11A11 | MULTIPLE | Y | 5.88 | 2.33 | 3.73 |
| | Q2_Q3 | PL11B8 | SINGLE | N | 2.81 | 2.89 | 2.33 |
| | Q2_Q3 | PL11B12 | MULTIPLE | Y | 5.78 | 2.16 | 2.35 |
| | Q2_Q3 | PL11C6 | MULTIPLE | Y | 2.82 | 5.98 | 2.71 |
| | Q2_Q3 | PL11D7 | SINGLE | N | 3.69 | 2.30 | 2.40 |
| | Q2_Q3 | PL11D9 | MULTIPLE | Y | 6.33 | 2.48 | 1.51 |
| | Q2_Q3 | PL11E1 | MULTIPLE | Y | 6.05 | 5.60 | 1.95 |
| | Q2_Q3 | PL11F4 | MULTIPLE | Y | 3.09 | 1.96 | 2.29 |
| | Q2_Q3 | PL11F6 | MULTIPLE | Y | 2.64 | 3.88 | 3.38 |
| | Q2_Q3 | PL11F12 | MULTIPLE | Y | 2.39 | 2.55 | 2.37 |
| | Q2_Q3 | PL11G2 | SINGLE | N | 3.24 | 4.30 | 2.04 |

**Table S5** Oligonucleotide primers and probes

Abbreviations: S, Sense; A, Antisense; iPCR, inverse PCR; vPCR, verification PCR

| OLIGO_ID | OLIGO_SEQUENCE | DESCRIPTION | ASSAY |
|---|---|---|---|
| IDT374 | AGCTGATCATGTAGTCGACTAATGCTTGAAACCCAGGAC | RIG-specific oligo-S | Hybridization probe preparation |
| IDT375 | AGCTGATCATGTAGTCGACAGTTGCGGATGTACTTCAG | RIG-specific oligo-AS | Hybridization probe preparation |
| IDT3705 | CAGTAAAGTCCTCTATCATCTCTGG | IS*S1*-specific probe-S | Hybridization probe preparation; PCR verification of IS*S1* |
| IDT3706 | CTAAATGCAGAACCAATAGAGGGCGC | IS*S1*-specific probe-AS | Hybridization probe preparation; PCR verification of IS*S1* |
| IDT5688 | /5Phos/GGGAATATTAGCTTAAGAACAAGAAGGATTATAAACCTTG | IS*S1*_3'END-S | PCR1, Illumina library, IS*S1* 3'-flank insertion site sequencing |
| IDT4426 | CAAGCAGAAGACGGCATACGAGAT**TTAGGC**GTGACTGGAGTTCAGACGTGTGCTCTTCCGATCtNNNNNNccttgtatttgattttaaactttgcaacagaacc | p7_IS*S1*_oligo_INDEX1 | PCR2, Illumina library, IS*S1* 3'-flank insertion site sequencing |
| IDT4427 | CAAGCAGAAGACGGCATACGAGAT**TGACCA**GTGACTGGAGTTCAGACGTGTGCTCTTCCGATCtNNNNNNccttgtatttgattttaaactttgcaacagaacc | p7_IS*S1*_oligo_INDEX2 | PCR2, Illumina library, IS*S1* 3'-flank insertion site sequencing |
| IDT4428 | CAAGCAGAAGACGGCATACGAGAT**ACAGTG**GTGACTGGAGTTCAGACGTGTGCTCTTCCGATCtNNNNNNccttgtatttgattttaaactttgcaacagaacc | p7_IS*S1*_oligo_INDEX5 | PCR2, Illumina library, IS*S1* 3'-flank insertion site sequencing |
| IDT4429 | CAAGCAGAAGACGGCATACGAGAT**GCCAAT**GTGACTGGAGTTCAGACGTGTGCTCTTCCGATCtNNNNNNccttgtatttgattttaaactttgcaacagaacc | p7_IS*S1*_oligo_INDEX6 | PCR2, Illumina library, IS*S1* 3'-flank insertion site sequencing |
| IDT4430 | CAAGCAGAAGACGGCATACGAGAT**CAGATC**GTGACTGGAGTTCAGACGTGTGCTCTTCCGATCtNNNNNNccttgtatttgattttaaactttgcaacagaacc | p7_IS*S1*_oligo_INDEX7 | PCR2, Illumina library, IS*S1* 3'-flank insertion site sequencing |
| IDT4431 | CAAGCAGAAGACGGCATACGAGAT**ACTTGA**GTGACTGGAGTTCAGACGTGTGCTCTTCCGATCtNNNNNNccttgtatttgattttaaactttgcaacagaacc | p7_IS*S1*_oligo_INDEX8 | PCR2, Illumina library, IS*S1* 3'-flank insertion site sequencing |
| IDT4432 | CAAGCAGAAGACGGCATACGAGAT**GATCAG**GTGACTGGAGTTCAGACGTGTGCTCTTCCGATCtNNNNNNccttgtatttgattttaaactttgcaacagaacc | p7_IS*S1*_oligo_INDEX9 | PCR2, Illumina library, IS*S1* 3'-flank insertion site sequencing |
| IDT4433 | CAAGCAGAAGACGGCATACGAGAT**TAGCTT**GTGACTGGAGTTCAGACGTGTGCTCTTCCGATCtNNNNNNccttgtatttgattttaaactttgcaacagaacc | p7_IS*S1*_oligo_INDEX10 | PCR2, Illumina library, IS*S1* 3'-flank insertion site sequencing |

**Table S5** Oligonucleotide primers and probes (continued)

| OLIGO_ID | OLIGO_SEQUENCE | DESCRIPTION | ASSAY |
|---|---|---|---|
| IDT4434 | CAAGCAGAAGACGGCATACGAGAT**GGCTAC**GTGACTGGAGTTCAGACGTGTGCTCTTCCGATCtNNNNNNccttgtatttgattttaaactttgcaacagaacc | p7_IS*S1*_oligo_INDEX11 | PCR2, Illumina library, IS*S1* 3'-flank insertion site sequencing |
| IDT4435 | CAAGCAGAAGACGGCATACGAGAT**CTTGTA**GTGACTGGAGTTCAGACGTGTGCTCTTCCGATCtNNNNNNccttgtatttgattttaaactttgcaacagaacc | p7_IS*S1*_oligo_INDEX12 | PCR2, Illumina library, IS*S1* 3'-flank insertion site sequencing |
| IDT4436 | CAAGCAGAAGACGGCATACGAGAT**AGTCAA**GTGACTGGAGTTCAGACGTGTGCTCTTCCGATCtNNNNNNccttgtatttgattttaaactttgcaacagaacc | p7_IS*S1*_oligo_INDEX13 | PCR2, Illumina library, IS*S1* 3'-flank insertion site sequencing |
| IDT4437 | CAAGCAGAAGACGGCATACGAGAT**AGTTCC**GTGACTGGAGTTCAGACGTGTGCTCTTCCGATCtNNNNNNccttgtatttgattttaaactttgcaacagaacc | p7_IS*S1*_oligo_INDEX14 | PCR2, Illumina library, IS*S1* 3'-flank insertion site sequencing |
| IDT4438 | CAAGCAGAAGACGGCATACGAGAT**ATGTCA**GTGACTGGAGTTCAGACGTGTGCTCTTCCGATCtNNNNNNccttgtatttgattttaaactttgcaacagaacc | p7_IS*S1*_oligo_INDEX15 | PCR2, Illumina library, IS*S1* 3'-flank insertion site sequencing |
| IDT4439 | CAAGCAGAAGACGGCATACGAGAT**CCGTCC**GTGACTGGAGTTCAGACGTGTGCTCTTCCGATCtNNNNNNccttgtatttgattttaaactttgcaacagaacc | p7_IS*S1*_oligo_INDEX16 | PCR2, Illumina library, IS*S1* 3'-flank insertion site sequencing |
| IDT4440 | CAAGCAGAAGACGGCATACGAGAT**TAGTTG**GTGACTGGAGTTCAGACGTGTGCTCTTCCGATCtNNNNNNccttgtatttgattttaaactttgcaacagaacc | p7_IS*S1*_oligo_INDEX17 | PCR2, Illumina library, IS*S1* 3'-flank insertion site sequencing |
| IDT4441 | CAAGCAGAAGACGGCATACGAGAT**GTCCGC**GTGACTGGAGTTCAGACGTGTGCTCTTCCGATCtNNNNNNccttgtatttgattttaaactttgcaacagaacc | p7_IS*S1*_oligo_INDEX18 | PCR2, Illumina library, IS*S1* 3'-flank insertion site sequencing |
| IDT4442 | CAAGCAGAAGACGGCATACGAGAT**GTGAAA**GTGACTGGAGTTCAGACGTGTGCTCTTCCGATCtNNNNNNccttgtatttgattttaaactttgcaacagaacc | p7_IS*S1*_oligo_INDEX19 | PCR2, Illumina library, IS*S1* 3'-flank insertion site sequencing |
| IDT4443 | CAAGCAGAAGACGGCATACGAGAT**GTGGCC**GTGACTGGAGTTCAGACGTGTGCTCTTCCGATCtNNNNNNccttgtatttgattttaaactttgcaacagaacc | p7_IS*S1*_oligo_INDEX20 | PCR2, Illumina library, IS*S1* 3'-flank insertion site sequencing |
| IDT4444 | CAAGCAGAAGACGGCATACGAGAT**GTTTCG**GTGACTGGAGTTCAGACGTGTGCTCTTCCGATCtNNNNNNccttgtatttgattttaaactttgcaacagaacc | p7_IS*S1*_oligo_INDEX21 | PCR2, Illumina library, IS*S1* 3'-flank insertion site sequencing |
| IDT4445 | CAAGCAGAAGACGGCATACGAGAT**CGTACG**GTGACTGGAGTTCAGACGTGTGCTCTTCCGATCtNNNNNNccttgtatttgattttaaactttgcaacagaacc | p7_IS*S1*_oligo_INDEX22 | PCR2, Illumina library, IS*S1* 3'-flank insertion site sequencing |

**Table S5** Oligonucleotide primers and probes (continued)

| OLIGO_ID | OLIGO_SEQUENCE | DESCRIPTION | ASSAY |
|---|---|---|---|
| IDT4446 | CAAGCAGAAGACGGCATACGAGAT**GAGTGG**GTGACTGGAGTTCAGACGTGTGCTCTTCCGATCtNNNNNNccttgtatttgattttaaactttgcaacagaacc | p7_IS*S1*_oligo_INDEX23 | PCR2, Illumina library, IS*S1* 3'-flank insertion site sequencing |
| IDT4447 | CAAGCAGAAGACGGCATACGAGAT**ACTGAT**GTGACTGGAGTTCAGACGTGTGCTCTTCCGATCtNNNNNNccttgtatttgattttaaactttgcaacagaacc | p7_IS*S1*_oligo_INDEX24 | PCR2, Illumina library, IS*S1* 3'-flank insertion site sequencing |
| IDT4448 | CAAGCAGAAGACGGCATACGAGAT**ATTCCT**GTGACTGGAGTTCAGACGTGTGCTCTTCCGATCtNNNNNNccttgtatttgattttaaactttgcaacagaacc | p7_IS*S1*_oligo_INDEX25 | PCR2, Illumina library, IS*S1* 3'-flank insertion site sequencing |
| IDT4449 | CAAGCAGAAGACGGCATACGAGAT**CTTTTG**GTGACTGGAGTTCAGACGTGTGCTCTTCCGATCtNNNNNNccttgtatttgattttaaactttgcaacagaacc | p7_IS*S1*_oligo_INDEX26 | PCR2, Illumina library, IS*S1* 3'-flank insertion site sequencing |
| IDT3858 | GTTCATTGATATATCCTCGCTGTC | 5'-IS*S1*-AS | iPCR, Sequencing, IS*S1* 5'-flank insertion site identification |
| IDT3859 | CCTGCGTTACAATCTAAGCTATCGTG | 5'-IS*S1*-S | iPCR, Sequencing, IS*S1* 5'-flank insertion site identification |
| IDT3860 | CCGTGACAATTACTCTTGGTTGACC | 3'-IS*S1*-AS | iPCR, Sequencing, IS*S1* 3'-flank insertion site identification |
| IDT3861 | GCCTCAACCACGATTAAGGGCATG | 3'-IS*S1*-S | iPCR, Sequencing, IS*S1* 3'-flank insertion site identification |
| IDT5419 | TCACTTCGTTGATAGACTTCCT | rlmH(PL7B7)-S | vPCR |
| IDT5420 | AAAGAAGCAGAGGCATTGAAA | rlmH(PL7B7)-AS | vPCR |
| IDT5421 | GCGACCACTTTGATGCAAT | recT(PL7E1)-S | vPCR |
| IDT5422 | GCTTTCTGTAGCAGGTTCATTC | recT(PL7E1)-AS | vPCR |
| IDT5467 | CATAACCTTCTTCAACGGCTTTAC | rmaC(PL1B12)-S | vPCR |
| IDT5468 | CATTGCTGAAACCGAAGAATCAA | rmaC(PL1B12)-AS | vPCR |
| IDT5606 | TACGATGGCTCCAGAATTTCC | chiA(PL9A4)-S | vPCR |
| IDT5607 | GATCCTGTTACGAGACGTTGAG | chiA(PL9A4)-AS | vPCR |
| IDT5612 | CTGTCCTATGACGGACAAGTTT | pstB(PL9B7)-S | vPCR |
| IDT5613 | TCTCGGGCCTCTGACAATA | pstB(PL9B7)-AS | vPCR |
| IDT5616 | TAATAGTGGTAGCGGGAGTAGG | xynD(PL9F7)-S | vPCR |
| IDT5617 | TGCAACAATCTGGTTCACTTTG | xynD(PL9F7)-AS | vPCR |
| IDT5618 | TCTTCCACTCTAACGTTCTCTT | yajF(PL9E3)-S | vPCR |
| IDT5619 | TTTCTCCCTTAGTTTGCGTTTA | yajF(PL9E3)-AS | vPCR |
| IDT5658 | GGTTTATGCCTGACCGTTTCT | ribB(PL2H5)-S | vPCR |
| IDT5659 | CCATCAATCGTAATCGAGCCTT | ribB(PL2H5)-AS | vPCR |

**Table S5** Oligonucleotide primers and probes (continued)

| OLIGO_ID | OLIGO_SEQUENCE | DESCRIPTION | ASSAY |
|---|---|---|---|
| IDT5668 | ATTGTGGCAACAGCACAG | yfeA(PL8B1)-S | vPCR |
| IDT5669 | CAAACAATTGCACCGTATTCTT | yfeA(PL8B1)-AS | vPCR |
| IDT6099 | GAAGTTAATTTATAAGGTGGGTTGGG | yabB/yabC(PL9D1)-S | vPCR |
| IDT6100 | AGTTCCATTGTGTTCCTCCTTTA | yabB/yabC(PL9D1)-AS | vPCR |
| IDT6101 | GAAGCGTTTAAAGTTCCAGTTACA | pi234(PL9D10)-S | vPCR |
| IDT6102 | CATGCCAATATCCAGCTCTAGG | pi234(PL9D10)-AS | vPCR |
| IDT6109 | GGCTTTGACGGTGTTGAAAT | yrfB(PL2A1)-S | vPCR |
| IDT6110 | AGGAGAAATACGGTAACCAATGA | yrfB(PL2A1)-AS | vPCR |
| IDT6111 | CAACAATCGCATCACCACTTG | yleE(PL2A3)-S | vPCR |
| IDT6112 | CTTCACCATTTAAGCCAACAGTATC | yleE(PL2A3)-AS | vPCR |
| IDT6113 | TGAAGAGGCCAAACGTCAA | yneD(PL2A7)-S | vPCR |
| IDT6114 | GCCAAAGGGTTTAACTTCCATC | yneD(PL2A7)-AS | vPCR |
| IDT6115 | TTTCGCCAGCGTCTTGATTA | gpo(PL2C2)-S | vPCR |
| IDT6116 | GCGAGTGGAGCAATTTCTTTG | gpo(PL2C2)-AS | vPCR |
| IDT6117 | GGTTTGCTACGGTCGTATACTT | ytdA/tuf(PL2C11)-S | vPCR |
| IDT6118 | GACGCCTAAATCGTCCATCAT | ytdA/tuf(PL2C11)-AS | vPCR |
| IDT6119 | CATAAGCTCCACCAAGCAATC | thrA(PL2E2)-S | vPCR |
| IDT6120 | TCAACCATTTAGCCCAGTTAGA | thrA(PL2E2)-AS | vPCR |
| IDT6121 | ACATGTGCCAAGGTCCAATTA | feoB(PL2G4)-S | vPCR |
| IDT6122 | GGTTGCTCCTAGTGCTTACTTT | feoB(PL2G4)-AS | vPCR |
| IDT6350 | GCCCACCTAAGCATGATAGAC | llrA(PL1A7)-S | vPCR |
| IDT6351 | GATGTTGTGACTCGTGAACATTTAG | llrA(PL1A7)-AS | vPCR |
| IDT6354 | GCTTTGCAGACAATTCGCTTTAT | yajH/yajA(PL1B4)-S | vPCR |
| IDT6355 | AATGAGTAGTAACCAGCGTTTCA | yajH/yajA(PL1B4)-AS | vPCR |
| IDT6356 | TGTTGTTACGACCATCACCTAC | arcB(PL1D4)-S | vPCR |
| IDT6357 | CCTTGGTGCTCATCCAGAAT | arcB(PL1D4)-AS | vPCR |
| IDT6358 | GAGAGCCTTCGTGGAATTGT | pepN(PL1D1)-S | vPCR |
| IDT6359 | AGGTAGAACCAAGAGAAAGCAATA | pepN(PL1D1)-AS | vPCR |
| IDT6360 | TACCAAGGAAGCCTTTGATTCT | ylbB(PL1B9)-S | vPCR |
| IDT6361 | CCCAAGATTTCCTCGCTCTT | ylbB(PL1B9)-AS | vPCR |
| IDT6364 | CCGCATGATGTGGTGTATCA | trpB(PL2A10)-S | vPCR |
| IDT6365 | TGTAATGGGCTCAGTTCTTGG | trpB(PL2A10)-AS | vPCR |
| IDT6366 | ACGACTCCAGACTTCTCCAA | yphJ(PL2A11)-S | vPCR |
| IDT6367 | GGAATTTGAACCTGTGACAGATAAAG | yphJ(PL2A11)-AS | vPCR |
| IDT6368 | AGGATTCGATACGCTGATTCAT | butB(PL2D3)-S | vPCR |
| IDT6369 | TTGAGCTGTTTCTTCGTTGTAATC | butB(PL2D3)-AS | vPCR |

**Table S5** Oligonucleotide primers and probes (continued)

| OLIGO_ID | OLIGO_SEQUENCE | DESCRIPTION | ASSAY |
|---|---|---|---|
| IDT6370 | CAGTTCCTGAATAGGCTCCTTC | yudK(PL3A4)-S | vPCR |
| IDT6371 | TTGACTCTTGTATCTTGGCTCTATC | yudK(PL3A4)-AS | vPCR |
| IDT6372 | CGAGTGGTTTGGATCCACTTAT | yjjC(PL3A11)-S | vPCR |
| IDT6373 | TGGCTTCCAGCTTCTTTACC | yjjC(PL3A11)-AS | vPCR |
| IDT6376 | GCCCTCTTAAGAATGGCTCTT | yreD(PL3D10)-S | vPCR |
| IDT6377 | GTGTATTGGTTTGCCTTGATTCT | yreD(PL3D10)-AS | vPCR |
| IDT6380 | GATTTGCGGAGTTTGCTGAC | hsdS(PL3E5)-S | vPCR |
| IDT6381 | GCTCGACTTTCCAGATGTGATA | hsdS(PL3E5)-AS | vPCR |
| IDT6387 | GGTAATGACGGTGTCTGAGTTA | yqaB(PL4D4)-AS | vPCR |
| IDT6388 | GAATGATGCTTCATGCTGGTAAA | zitP(PL4G1)-S | vPCR |
| IDT6389 | CTGGCTTGATTGCCTCTTACTA | zitP(PL4G1)-AS | vPCR |
| IDT6390 | ACAGGTGATCGGGTTGAAATTA | spoT(PL5A3)-S | vPCR |
| IDT6391 | GCATACAAAGCTTCCGCATTAC | spoT(PL5A3)-AS | vPCR |
| IDT6392 | AGCGCTTTACTATCTGCTTTCT | fbp(PL5A7)-S | vPCR |
| IDT6393 | CAGTCGTCGAATCCACTTCAT | fbp(PL5A7)-AS | vPCR |
| IDT6394 | GCCGTCGTCATGCTCATTAAA | yqfD(PL5A10)-S | vPCR |
| IDT6395 | TGGGATAGGAATTGCGTTTGG | yqfD(PL5A10)-AS | vPCR |
| IDT6396 | TGCTCAATAAGTCGTCCTTCC | coiA(PL5A12)-S | vPCR |
| IDT6397 | GCCTCACTTTGCTCATAAATCTC | coiA(PL5A12)-AS | vPCR |
| IDT6398 | GATAGTCCGGCATCAACAAGA | kinC(PL5B12)-S | vPCR |
| IDT6399 | TCCATCAGTGACCAAGGAATG | kinC(PL5B12)-AS | vPCR |
| IDT6400 | CGCATTGAACTGACGTGAATC | yffB(PL5C12)-S | vPCR |
| IDT6401 | TACGCTCTACTCCAGGGAAA | yffB(PL5C12)-AS | vPCR |
| IDT6402 | AACCTACGCCAGAGTCATTTC | yafE(PL5D12)-S | vPCR |
| IDT6403 | TCAGTGCCTGCACGTTATT | yafE(PL5D12)-AS | vPCR |
| IDT6404 | GACAGGAATGCCAGCAAATAAC | yejI(PL5E11)-S | vPCR |
| IDT6405 | GGAGCAAAGTCGGCAGATAA | yejI(PL5E11)-AS | vPCR |
| IDT6406 | GCTCAACGGCACTGAAATAAA | pi144(PL5G8)-S | vPCR |
| IDT6407 | ACCAGTTGCACGACCTAAA | pi144(PL5G8)-AS | vPCR |
| IDT6408 | TGAAGTTGCTCATGCTTTATTGG | rpoE(PL6A8)-S | vPCR |
| IDT6409 | TGTGCCTTCTTCATCTTCTGG | rpoE(PL6A8)-AS | vPCR |
| IDT6410 | CGGAACTGGGCGAAGAATTTA | rsmE(PL6B3)-S | vPCR |
| IDT6411 | CCAAATGGCTGTAGCTCCTAAT | rsmE(PL6B3)-AS | vPCR |
| IDT6412 | GCAACAGAACTGGTTGGAAAG | dhaL(PL6D10)-S | vPCR |
| IDT6413 | AGTGACAAGCCTGCCATATC | dhaL(PL6D10)-AS | vPCR |
| IDT6414 | GCGGTACTTGCACCTATCTT | ycfA(PL6D11)-S | vPCR |

**Table S5** Oligonucleotide primers and probes (continued)

| OLIGO_ID | OLIGO_SEQUENCE | DESCRIPTION | ASSAY |
|---|---|---|---|
| IDT6415 | AAACTAACTGTTTGGCCTGTTG | ycfA(PL6D11)-AS | vPCR |
| IDT6416 | GGGCAGATCAGACTCCAAATAA | ysdB(PL6E4)-S | vPCR |
| IDT6417 | GAGCAGATGCCCGGATAAA | ysdB(PL6E4)-AS | vPCR |
| IDT6418 | CCGACGAATAAATTCCGCTAGT | coiA(PL6F9)-S | vPCR |
| IDT6419 | TGAATTTAAGCAGAGGCCAGAT | coiA(PL6F9)-AS | vPCR |
| IDT6420 | CCAGAAGGAGCAAGCGAAA | celA(PL7B8)-S | vPCR |
| IDT6421 | CGAACATGAACATCTGCAAACC | celA(PL7B8)-AS | vPCR |
| IDT6422 | AGTGTACAGGCCGCATTT | hisH(PL7D6)-S | vPCR |
| IDT6423 | AGTCCAAGACCTTGTCTTTCTT | hisH(PL7D6)-AS | vPCR |
| IDT6424 | AAAGTCGGAACGAAGAGGAG | ogt(PL7A5)-S | vPCR |
| IDT6425 | TCTAAATTCCGTTACTTCTGGTTTC | ogt(PL7A5)-AS | vPCR |
| IDT6426 | CTCCATTGATTCGCTTGTTGTT | yugA/yugB(PL7G12)-S | vPCR |
| IDT6427 | ATGCCAGAGTCCTAATGAAAGG | yugA/yugB(PL7G12)-AS | vPCR |
| IDT6430 | ACGGAATGATGTCAGGAATGT | glnP(PL8F1)-S | vPCR |
| IDT6431 | GCCCTTGTTTAATGGCGTATTT | glnP(PL8F1)-AS | vPCR |
| IDT6438 | GCACCATCGCCACCTTTA | ftsQ(PL10B7)-S | vPCR |
| IDT6439 | GACGGAAATCAGGTTCGAGTT | ftsQ(PL10B7)-AS | vPCR |
| IDT6440 | AGAAGATGGTCGCCAAGATAAA | aroF/ptsH(PL10C11)-S | vPCR |
| IDT6441 | TGAATGGTTGCTAGACTTTGATTTC | aroF/ptsH(PL10C11)-AS | vPCR |
| IDT6442 | GTACTTCCGTACAACTCCAGTC | optB(PL10C12)-S | vPCR |
| IDT6443 | GAAAGAGCGATTGTTGGCATAA | optB(PL10C12)-AS | vPCR |
| IDT6444 | TAGGGAGCGTTGAAAGACATC | yojC(PL10H2)-S | vPCR |
| IDT6445 | GCGAGGAGGGAAGCAAA | yojC(PL10H2)-AS | vPCR |
| IDT6446 | TGTCATGTTCTTTCCTCTTTCT | ysaD(PL11A10)-S | vPCR |
| IDT6447 | AGAAGTCAGTGATAATTCAGGA | ysaD(PL11A10)-AS | vPCR |
| IDT6450 | TTCCCAAGCAGAACCAGAAA | dexA(PL11B1)-S | vPCR |
| IDT6451 | CAATCGCCAATGGATGATAATGG | dexA(PL11B1)-AS | vPCR |
| IDT6452 | AGTTAGCTCTGCTTCCCATTC | coiA(PL11D6)-S | vPCR |
| IDT6453 | GGAATTTATTCGTCGGCAACTTTA | coiA(PL11D6)-AS | vPCR |
| IDT6472 | ATGGAGTGGCCTCAATCTTC | yajA(PL1F11)-S | vPCR |
| IDT6473 | ATGAGTAGTAACCAGCGTTTCA | yajA(PL1F11)-AS | vPCR |
| IDT6474 | TGCCATTCAACAAGCCAAAG | ftsY/prsA(PL1H4)-S | vPCR |
| IDT6475 | GGGCTAATTCGTGGTTAGAAGA | ftsY/prsA(PL1H4)-AS | vPCR |
| IDT6476 | GGTGGCTTTCGTAATCGTAAAC | celA(PL1H7)-S | vPCR |
| IDT6477 | GAGCCGCTTGATACATCATTTC | celA(PL1H7)-AS | vPCR |
| IDT6482 | CAACATGGCGAGGAAATGATAC | tagX(PL4G9)-S | vPCR |

**Table S5** Oligonucleotide primers and probes (continued)

| OLIGO_ID | OLIGO_SEQUENCE | DESCRIPTION | ASSAY |
|---|---|---|---|
| IDT6483 | ATCCTGATCCCAAGCATAGAAA | tagX(PL4G9)-AS | vPCR |
| IDT6484 | TAGAAGCCTCACCTATGCAATATC | ymbA(PL4G10)-S | vPCR |
| IDT6485 | AACCCTGTATACAATAGGAAGACTG | ymbA(PL4G10)-AS | vPCR |
| IDT6486 | CCAGAGGGACTCATTCAGTAGA | ylfC(PL4G11)-S | vPCR |
| IDT6487 | GGGTAGCTTTAACTACACTCTATTCTT | ylfC(PL4G11)-AS | vPCR |
| IDT6488 | AAGCACCAAGTGGCATATAGAC | yahI(PL4D6)-S | vPCR |
| IDT6489 | GGATTTGAGGCAGCAGAACT | yahI(PL4D6)-AS | vPCR |
| IDT6490 | TGACGGAAGATATGGCTTGATAG | ps201(PL5E2)-S | vPCR |
| IDT6491 | CAGTAGATTTGAGTGACCGAGAA | ps201(PL5E2)-AS | vPCR |
| IDT6492 | TCTGGAAATAAAGTGACTGTGGA | ywaI(PL8C1)-S | vPCR |
| IDT6493 | CTACGCGTTGAGGCTAAAGA | ywaI(PL8C1)-AS | vPCR |
| IDT6494 | CATACGGCTAAAGTCCAAACCT | gltB(PL8E9)-S | vPCR |
| IDT6495 | GCAATGCTAGGTGCTGAAGA | gltB(PL8E9)-AS | vPCR |
| IDT6496 | GCTATGGAGCTTAGACGATTTGG | ydcF(PL8F5)-S | vPCR |
| IDT6497 | TGCAAACAATTGGCTTGTCAAA | ydcF(PL8F5)-AS | vPCR |
| IDT6498 | GTGGAAGTCGGGAGTCATTTAT | cadA(PL9D2)-S | vPCR |
| IDT6499 | ACTGTGATTCTGCCGCTATC | cadA(PL9D2)-AS | vPCR |
| IDT6500 | AATGCTGCGGTTCCTTGA | carB(PL9H6)-S | vPCR |
| IDT6501 | ACACAGAAGGCTTAGCAGAAA | carB(PL9H6)-AS | vPCR |
| IDT6502 | GTAAGCTGTCCGGGCATAA | pi1231(PL9B2)-S | vPCR |
| IDT6503 | CACGTTCTCCGACTCATCAA | pi1231(PL9B2)-AS | vPCR |
| IDT6510 | ACTGCCAAGAAATACGTCAAATG | yliE(PL10C4)-S | vPCR |
| IDT6511 | TGCGCCAATCACTGATCTT | yliE(PL10C4)-AS | vPCR |
| IDT6512 | GGATGCCCAATGGGAGAAA | yrjC(PL10D3)-S | vPCR |
| IDT6513 | CCGTTTAACATAATCATTGGCTTCT | yrjC(PL10D3)-AS | vPCR |
| IDT6514 | ATCAATCAGGAACAATCCGTGT | arcC3(PL10G9)-S | vPCR |
| IDT6515 | AGCTCTAGGAGTTGTACCAGTTA | arcC3(PL10G9)-AS | vPCR |
| IDT6516 | GAGGTTTAAGGATGAACAAGAAGATG | gltS(PL11D7)-S | vPCR |
| IDT6517 | CATCAGAACCCACGACTGTATT | gltS(PL11D7)-AS | vPCR |
| IDT6520 | GCAGGCCCACCATTGATA | oppA(PL11G2)-S | vPCR |
| IDT6521 | GTGGCTCTAACCAGAACTCAA | oppA(PL11G2)-AS | vPCR |
| IDT6535 | CGGAAGTTTATGGCGCTATTT | ydhC/ydhD(PL4A12)-S | vPCR |
| IDT6536 | AACCTTTCCACGATTTCTCTTAAC | ydhC/ydhD(PL4A12)-AS | vPCR |
| IDT6537 | CCCTCTCCTGTATTCGCTTTATT | oriC(PL8C4)-S | vPCR |
| IDT6538 | AGGGATGCCATATTACTCCTTTAC | oriC(PL8C4)-AS | vPCR |
| IDT6539 | CCAGGCTGAGGATGTTCTTT | tra904(PL8H3)-S | vPCR |

**Table S5** Oligonucleotide primers and probes (continued)

| OLIGO_ID | OLIGO_SEQUENCE | DESCRIPTION | ASSAY |
|---|---|---|---|
| IDT6540 | CTTCGCTTTCAAGACCCAATTT | tra904(PL8H3)-AS | vPCR |
| IDT6541 | CTAAATATGGCTCGGACAGGTAG | ilvC(PL6C5)-S | vPCR |
| IDT6542 | AGAGCGTTGCTCATCAGATAAT | ilvC(PL6C5)-AS | vPCR |
| IDT6549 | GACAGCTCTGTCAGTGATT | yfdA(PL4G3)-S | vPCR |
| IDT6550 | TCCTTTGCTACTTCCAACG | yfdA(PL4G3)-AS | vPCR |
| IDT6559 | TCTGCCCAACGCGTATTT | yujF(PL8B7)-S | vPCR |
| IDT6560 | GAAGACGGATTGACGCTATGT | yujF(PL8B7)-AS | vPCR |
| IDT6561 | CCATTGGTGGCTTTAACTCTAATG | yrgH(PL8F11)-S | vPCR |
| IDT6562 | GCAGAAGATGACGAGCTTGA | yrgH(PL8F11)-AS | vPCR |
| IDT6563 | GCCAGCTGAACAAACGATAAAG | ybaK(PL9C11)-S | vPCR |
| IDT6564 | CTGGCCCTGTCATTGGTATT | ybaK(PL9C11)-AS | vPCR |
| IDT6567 | GGTCAATTTAACAACGACCCAAA | ypcB/ypcC(PL11B8)-S | vPCR |
| IDT6568 | ACCAACACAAGGAGCAGATAC | ypcB/ypcC(PL11B8)-AS | vPCR |
| IDT6573 | CTCTCTCCTGGGAAGTTTGTTT | ymgF/ymgG(PL3B11)-S | vPCR |
| IDT6574 | GGCCTCTATTGTGAGGCTTT | ymgF/ymgG(PL3B11)-AS | vPCR |
| IDT6837 | AGTTGGGACCTTATCCATGAATAA | rsmB(PL1B3)-S | vPCR |
| IDT6838 | TGACGAAAGTCATTGACAGTCAT | rsmB(PL1B3)-AS | vPCR |
| IDT5059 | AAAAAAACGCGTCGAATCGCCAACGTTTTCG | pLNRK-specific probe | Northern blotting |
| IDT1073 | GTACCTTAAACTACTTGACTTAACACC | Intron-specific probe | Northern blotting |
| IDT861 | CGAGCTGACGACAACCATGCACCACC | 16S rRNA-specific probe | Northern blotting |
| IDT5546 | ATTACAAAAGTAACGAATCGCCAACGTTTTCG | pLNRK-specific primer-S | Curing-PCR verification for loss of pLNRK plasmid |
| IDT5547 | TGTTCTTGGATATCCTTCTTCAACTAACGGGGC | pLNRK-specific primer-AS | Curing-PCR verification for loss of pLNRK plasmid |
| IDT6054 | cactcaccatgggtactgcaATGAGCAAAGGAGAAGAAC | NEBuilder_pLNRK_GFP-S | Making pLNRK-GFP |
| IDT6055 | aaagcttgagctctctagaaTTATTTGTAGAGCTCATCC | NEBuilder_pLNRK_GFP-AS | Making pLNRK-GFP |
| IDT6056 | CACTCACCATGGGTACTGCACTGCAGATGAGCAAAGGAGAAGAAC | GFP specific primer-S | PCR confirmation of GFP transformants |
| IDT6057 | AAAGCTTGAGCTCTCTAGAAACTAGTTTATTTGTAGAGCTCATCC | GFP specific primer-AS | PCR confirmation of GFP transformants |
| IDT6871 | GGAG**GAATTC**ATATTATTGTCGATAACGCGAGC | PnisA-S for pGh5 (*Eco*RI) | Complementation |
| IDT6872 | GGG**AAGCTT**TGTCATCAGGAGAAAATGATTGG | PnisA-AS for pGh5 (*Hin*dIII) | Complementation |
| IDT6924 | ctatagggcgaattgggtacTTACTTATGATAAGCCGAACCTCG | rlmH - pGh5:PnisA-S (kpnI) | Complementation |
| IDT6925 | ctcgaggggggggcccggtacATGAAGATTAAATTAGTAGTTGTTGGAAAAC | rlmH - pGh5:PnisA-AS (kpnI) | Complementation |
